# Quality of malaria case management at outpatient health facilities in Angola

**DOI:** 10.1186/1475-2875-8-275

**Published:** 2009-12-02

**Authors:** Alexander K Rowe, Gabriel F Ponce de León, Jules Mihigo, Ana Carolina FS Santelli, Nathan P Miller, Pedro Van-Dúnem

**Affiliations:** 1 Malaria Branch, Division of Parasitic Diseases (DPD), Centers for Disease Control and Prevention (CDC), Atlanta, USA; 2 Malaria Branch, DPD, CDC, Luanda, Angola; 3 National Malaria Program Office, Secretariat for Surveillance in Health, Ministry of Health, Brasilia, Brazil; 4 The MENTOR Initiative, Huambo, Angola; 5 Angola National Malaria Control Program, Ministry of Health, Luanda, Angola

## Abstract

**Background:**

Angola's malaria case-management policy recommends treatment with artemether-lumefantrine (AL). In 2006, AL implementation began in Huambo Province, which involved training health workers (HWs), supervision, delivering AL to health facilities, and improving malaria testing with microscopy and rapid diagnostic tests (RDTs). Implementation was complicated by a policy that was sometimes ambiguous.

**Methods:**

Fourteen months after implementation began, a cross-sectional survey was conducted in 33 outpatient facilities in Huambo Province to assess their readiness to manage malaria and the quality of malaria case-management for patients of all ages. Consultations were observed, patients were interviewed and re-examined, and HWs were interviewed.

**Results:**

Ninety-three HWs and 177 consultations were evaluated, although many sampled consultations were missed. All facilities had AL in-stock and at least one HW trained to use AL and RDTs. However, anti-malarial stock-outs in the previous three months were common, clinical supervision was infrequent, and HWs had important knowledge gaps. Except for fever history, clinical assessments were often incomplete. Although testing was recommended for all patients with suspected malaria, only 30.7% of such patients were tested. Correct testing was significantly associated with caseloads < 25 patients/day (odds ratio: 18.4; p < 0.0001) and elevated patient temperature (odds ratio: 2.5 per 1°C increase; p = 0.007). Testing was more common among AL-trained HWs, but the association was borderline significant (p = 0.072). When the malaria test was negative, HWs often diagnosed patients with malaria (57.8%) and prescribed anti-malarials (60.0%). Sixty-six percent of malaria-related diagnoses were correct, 20.1% were minor errors, and 13.9% were major (potentially life-threatening) errors. Only 49.0% of malaria treatments were correct, 5.4% were minor errors, and 45.6% were major errors. HWs almost always dosed AL correctly and gave accurate dosing instructions to patients; however, other aspects of counseling needed improvement.

**Conclusion:**

By late-2007, substantial progress had been made to implement the malaria case-management policy in a setting with weak infrastructure. However, policy ambiguities, under-use of malaria testing, and distrust of negative test results led to many incorrect malaria diagnoses and treatments. In 2009, Angola published a policy that clarified many issues. As problems identified in this survey are not unique to Angola, better strategies for improving HW performance are urgently needed.

## Background

Malaria is a major cause of morbidity and mortality in sub-Saharan Africa, and prompt treatment with an effective anti-malarial is a pillar of malaria control. For decades, as most health facilities in Africa did not perform diagnostic testing, uncomplicated malaria was typically managed with a syndromic approach in which all patients with febrile illness were treated with chloroquine. Since 2000, in the face of increasing resistance to chloroquine and other commonly-used anti-malarials, 45 African countries changed their case-management policies to recommend artemisinin-based combination therapy (ACT) as the first-line treatment for uncomplicated malaria [1,2]. To reduce unnecessary ACT use, malaria diagnostic testing with microscopy or rapid diagnostic tests (RDTs) is often recommended.

Despite substantial investments by donors, African governments, and other partners to support these new diagnostic and treatment policies, remarkably little is known about how well the policies have been implemented. Published studies of health worker adherence to ACT policies have only been done in three low-income countries (Kenya, Uganda, and Zambia) [3-9]; and use of testing was evaluated in only two (Kenya and Zambia) [5-7]. Results from these studies are concerning. The proportion of patients needing ACT who received it was often low (range: 26-64%), as was the proportion of patients needing testing who were tested (range: 27-56%). Notably, one study from an upper-middle income country (South Africa, where malaria transmission is very limited and health facilities in malaria endemic areas are staffed with personnel from the malaria control programme to perform testing) [1] found that all patients with a febrile illness were tested and all test-positive patients were treated with anti-malarials.

Although malaria transmission, malaria control programs, and levels of socioeconomic development vary widely across Africa, evaluations of real-world implementation of ACT in specific settings can reveal programmatic strengths, weaknesses, and lessons that might benefit other parts of the continent. This report presents such an evaluation in Huambo Province, Angola, where the scale-up of ACT and diagnostic testing was supported by the U.S. President's Malaria Initiative [10]. First, a narrative is provided that describes how the ACT policy was implemented. Second, a survey is presented that assessed health facility and health worker readiness to manage malaria cases and the quality of malaria case management.

### ACT implementation

Angola's ACT policy was adopted in 2004, although as early as 2003 ACT had been supplied and short trainings conducted in some health facilities in Huambo. In February 2006, the National Malaria Control Programme (NMCP) began disseminating the policy with a five-day training course for 20 trainers from across Angola, including one from Huambo. While many trainings were held over the next several years, it is notable that the ACT policy document was only finalized in 2008 and published in 2009 [11]. Furthermore, in 2007 (when the survey described in this report was being planned), the draft policy document was somewhat ambiguous on malaria diagnosis [12]. The document defined a malaria case as anyone with fever and a positive test for *Plasmodium*. It then had a "clinical case" section that listed signs and symptoms of uncomplicated malaria without further explanation. The document stated that malaria cannot be ruled out in patients with a negative test and no other cause of fever and that the World Health Organization (WHO) recommends that malaria can be diagnosed among children < 5 years old (under-5s) based on clinical findings without laboratory testing. While these statements provided general guidance on diagnosing malaria, they left some questions. For example: 1) should under-5s with fever be tested?; 2) how should non-fever signs and symptoms be used to decide which patients should be tested?; and 3) in patients with fever and a negative test, which non-malaria causes of fever should be ruled out before treating with an anti-malarial? (Note that the recently published policy clarifies many of these issues--see details below [11].)

In Huambo Province, the recommended ACT was artemether-lumefantrine (AL). In 2007, in response to the ambiguous policy, local malaria control staff developed training materials that were based on the draft NMCP policy document, as well as WHO guidelines when the NMCP policy was unclear. However, local staff did not want to be seen as making their own policy. Therefore, although the training materials provided some additional precision on what health workers should do during consultations, they were still not a comprehensive case-management guideline. From August-September 2006, the training materials were used in 3-day courses for health workers from 16 health facilities. From January-November 2007, 570 staff throughout the province were trained. Courses typically lasted three days, involved clinicians, pharmacists and laboratory technicians, and included instruction on RDT use. Participants were expected to share the information with others in their facilities (i.e., informal, cascade training), although there was a multi-year plan to train additional health workers with the formal three-day course. A multi-year plan was needed because the public sector employs unusually large numbers of health workers (after Angola's civil war, many nurses from the opposition faction were integrated into the health system). Scale-up activities were conducted with substantial external assistance, including staff from the NMCP, WHO, The MENTOR Initiative (a non-governmental organization [13]), the U.S. Agency for International Development, and the U.S. Centers for Disease Control and Prevention.

In September 2007 (a time when the availability of malaria testing was still quite limited in Angola), the NMCP Director modified the ACT policy to be in line with WHO guidelines [14]: under-5s with suspected malaria in areas with moderate or high transmission did not need testing and could be presumptively treated for malaria. The change was announced during a meeting, but not incorporated into a published policy until 2009 [10]. Interestingly, one year after the September 2007 announcement, partners sometimes still had different understandings of what presumptive treatment meant (treat all under-5s with suspected malaria versus only treat if no other cause of fever was found). The recently published policy specifies that under-5s with a febrile illness from areas with moderate or high malaria transmission should be treated for malaria without testing [11]. Other issues clarified by the published policy include the definition of a confirmed malaria case, how patients should be managed if malaria testing is not available, and the possibility that patients with cerebral malaria can have a negative test.

AL and RDTs were usually first supplied with the trainings. Subsequently, the pharmaceutical management plan called for monthly deliveries to health facilities. In practice, the time period varied. For several months before the survey described in this report, commodities were not supplied because of transportation difficulties from the central warehouse in Luanda, Angola's capital. However, commodities were delivered shortly before the survey.

Regarding other ACT implementation activities before the survey, AL-related supervision and microscopy training were just beginning. Regarding user fees, the policy calls for free ACT and RDT testing at public facilities.

## Methods

### Study setting

Huambo Province, in the highlands of central Angola, has meso-endemic, stable malaria transmission [15]. Transmission peaks from November-April, with *Plasmodium falciparum *causing > 90% of infections. The population (2.3 million) is impoverished, and agriculture is a primary source of economic activity [16]. About half (45%) live in rural areas; and there is one urban center, the city of Huambo. During Angola's long civil war (1975-2002), Huambo was particularly hard hit. Much of the health infrastructure was left severely damaged or destroyed, and many land mines remain.

### Study design and inclusion criteria

We conducted a cross-sectional cluster survey from October-November, 2007. A cluster was defined as all patient consultations performed in an eligible health facility during regular working hours (Monday-Friday, 8 am-3 pm) during the survey period. Facilities were eligible if they were a public or private facility in Huambo Province that provided outpatient care and where the ACT policy had been implemented (i.e., at least one health worker trained on the policy and ACT delivered anytime in the past). Fifty-seven eligible facilities were identified (eight hospitals, 48 health centers, and one health post); all were government-run. Health workers were eligible if they performed outpatient consultations with sick patients at an eligible facility. Inclusion criteria for outpatient consultations were any initial consultation for patients seeking care for any illness at eligible facilities during regular working hours. Initial consultation meant the patient's first visit to the facility for the current illness.

### Sample size and sampling

To minimize costs, a sample size of 30 health facilities was chosen, which is the smallest number of clusters that many experts advise for cluster surveys [17]. Assuming 10% of facilities would be permanently closed or inaccessible, the total sample size was 33 facilities. The list was ordered by facility type (hospitals, health centers, and health posts) and municipality (i.e., district), and 33 facilities were selected with systematic sampling. Systematic sampling was used to choose dates for each facility visit.

Survey teams attempted to include all initial consultations using the "follow-the-patient" approach (see below). However, in high-volume clinics, not all consultations could be included. Surveyors reviewed patient registers and averaged patients in the preceding 5 weekdays to estimate daily caseload. Estimated caseload determined the sampling fraction of patients: if < 21 patients were expected, the sampling fraction was 100% (include all initial consultations); if 22-42 patients were expected, the sampling fraction was 50% (every other initial consultation), etc. If not all consultations could be included, patients were selected with systematic sampling (e.g., select every third patient, with the first chosen randomly).

### Data collection

Two teams collected data, each consisting of three surveyors (all medical professionals), a laboratory technician, and a driver. Survey organizers closely supervised data collection. For eight days, surveyors practiced survey procedures in a classroom setting and in health facilities not in the sample. Concordance testing assessed observation skills (with role-playing and videos of consultations) and re-examination skills (with hospitalized patients), and training continued until concordance (i.e., percent agreement between surveyors and a gold standard determined by survey organizers) was at least 90%; concordance was typically about 96%.

Data were collected with standardized forms written in Portuguese. Questions for patients were translated into Umbundu, the local language and back-translated to verify accuracy. Interviews were conducted in the language that subjects were most comfortable speaking.

Dates of survey visits were not announced in advance to health facility staff. During visits, surveyors arrived before 8 am. Teams met with facility directors and health workers performing consultations to introduce themselves, describe the visit's purpose, and request verbal consent from health workers.

As ill patients arrived at facilities, drivers identified patients coming for an initial consultation and gave these patients a card with a sequential identification number. The cards were used to sample patients and confirm the total number of initial consultations that day. Surveyors met selected patients and requested verbal consent. Surveyors silently observed consultations with a checklist to record health worker practices. After the consultation, surveyors asked health workers for the patient's diagnoses and prescribed treatments. Surveyors then followed patients through all other parts of the facility visit (e.g., laboratory, pharmacy) and silently recorded malaria-related treatment instructions and counseling messages. When patients were ready to leave the facility, surveyors conducted an interview and focused re-examination in a room out of view of the health facility staff to avoid influencing routine practices. Dispensed medications were recorded. If surveyors identified a patient who had not been adequately treated for malaria or any other serious illness, they provided treatment free of charge.

Non-pregnant patients ≥ 5 years old with suspected malaria (defined below) were tested. Survey team laboratory technicians drew several drops of blood by finger stick with a single-use sterile lancet to make a blood smear and perform a RDT (Paracheck^®^, Orchid Diagnostics, Mumbai, India). Blood smears were air dried in the field; after the survey, they were stained with Giemsa and read by an expert microscopist. The RDT was read in the field after 15-25 minutes to ensure that parasitemic patients were treated with an anti-malarial. The "old" (pre-September 2007) NMCP guidelines recommended testing for all patients with suspected malaria, however the survey did not test under-5s and pregnant women with suspected malaria. The reasons were to minimize unnecessary patient discomfort and the time patients spent with surveyors. Due to a misunderstanding, it was thought the policy stated that these two patient groups should be treated with an anti-malarial regardless of the test result. Therefore, as testing did not seem to affect the treatment, these patients were not tested.

At the end of the visit, surveyors interviewed health workers to obtain information on training, supervision, and knowledge of malaria case management. Surveyors also assessed the facility's equipment and drug stocks.

### Definitions

**Figure 1:**
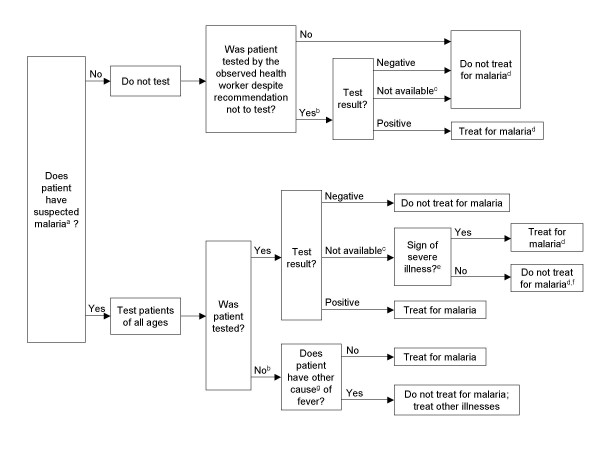
**Algorithm used to analyse the quality of malaria diagnosis and treatment, as it was applied in outpatient health facilities, Huambo Province, Angola**. ^a ^Defined as either fever (history of fever or axillary temperature > 37.5°C), or at least 3 of the following: headache, joint pain, chills, sweating, anaemia (palmor pallor), cough (applies to children only), anorexia, fatigue, vomiting, or diarrhoea. ^b ^Error (health worker's decision did not follow policy documents and training materials). ^c ^Result not available on the day of the consultation, patient asked to return the next day (this only occurred for 2 patients). ^d ^This part of the algorithm was not explicitly included in policy documents or training materials; however, the decision could be logically inferred from policy documents or training materials. ^e ^Defined as cerebral dysfunction, cerebral malaria, disseminated intravascular coagulopathy, haemoglobinuria, hepatic dysfunction, hyperthermia, pulmonary oedema, renal insufficiency, severe anaemia, or shock. For details, see Figure 2. ^f ^Do not treat for malaria now; wait until result is ready and treat only if test is positive. ^g ^Defined as dysentery, hepatitis, influenza-like illness, measles, otitis, pneumonia, or urinary tract infection. For details, see Figure 2.

Defining the standard for assessing the quality of malaria testing, diagnosis, and treatment was challenging because: 1) policy and training materials sometimes lacked precision; 2) the survey protocol did not include testing for all patients who should have been tested; and 3) the NMCP policy changed just before the survey. Therefore, using NMCP guidelines, an analysis algorithm was developed with enough precision to analyze case-management quality in our sample of consultations (Figure 1). The algorithm was based on the "old" pre-September 2007 policy (test all patients with suspected malaria) as implemented in Huambo Province because the "new" policy (announced in September 2007, which recommended presumptive treatment for under-5s) was just starting in Huambo--only three health workers had been trained in the new policy, and they only saw four patients in the analysis. The algorithm incorporated case-management principles actually conveyed to health workers during training and provided disease classifications for the somewhat ambiguous instances in which patients that should have been tested were not and vice-versa.

The analysis algorithm was used to make a "gold standard" determination of the patient's diagnosis and treatment. Case-management quality was assessed by comparing the observed health worker's diagnosis and treatment with the gold standard. The analysis algorithm did not use survey laboratory results; instead, it used information that should have been available to health workers (e.g., results of microscopy and RDTs ordered by health workers). This approach is best for evaluating adherence to a guideline, as it prevents classifying health worker practices as erroneous if laboratory results from the health facility did not match survey laboratory results. Survey laboratory results were used in separate analyses. The analysis algorithm used clinical signs and symptoms from patient interviews and re-examinations performed by surveyors.

**Figure 2:**
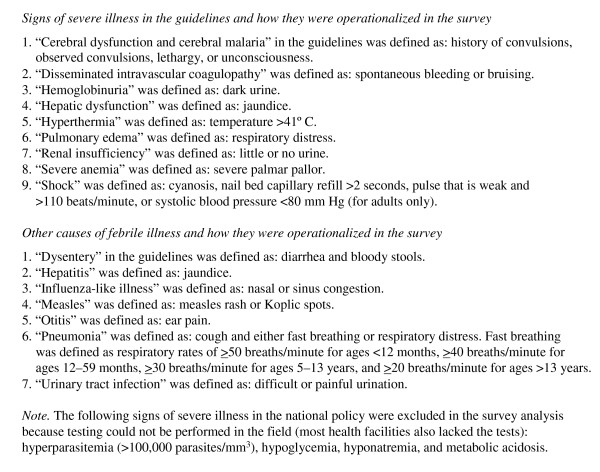
**Definitions used in the survey analysis**.

As per the Huambo training materials, suspected malaria was defined as either fever (by history or measured axillary temperature > 37.5°C) or at least three of the following: headache, joint pain, chills, sweating, anaemia, cough (for children only), anorexia, fatigue, vomiting, or diarrhoea. Uncomplicated malaria was defined as malaria (determined by the analysis algorithm) without signs of severe illness (Figure 2), and complicated malaria was defined as malaria with at least one sign of severe illness. Definitions of non-malaria causes of fever used by the algorithm are shown in Figure 2.

**Figure 3:**
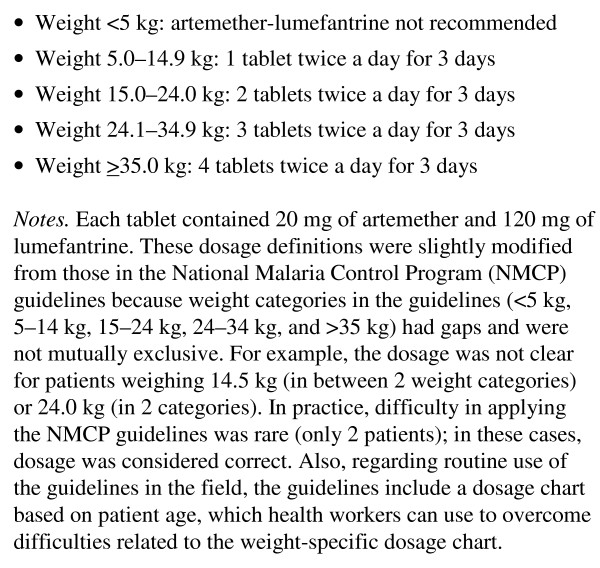
**Dosage for artemether-lumefantrine used in the survey analysis**.

Definitions of treatment quality were based on surveyor-measured patient weights and the NMCP's anti-malarial dosing guidelines [12] (Figure 3). Malaria treatment quality was categorized as: 1) recommended (health worker's prescribed treatment exactly matched the analysis algorithm, including drug type, dosage, and treatment duration), 2) adequate (treatments were not recommended, but still considered life-saving), or 3) inadequate (neither recommended nor adequate). These three categories correspond to correct treatment, minor errors, and major errors, respectively [18].

### Analysis

Data were double-entered with SPSS Data Entry version 1.0 (SPSS Inc., Chicago, Illinois) and validated against paper questionnaires. Analyses were conducted with SAS version 9.1 (SAS Institute, Inc., Cary, North Carolina). Weights (for patient-level indicators only) were the product of two components: (1/patient selection probability) × a non-response adjustment (number of eligible patients selected for enrollment [i.e., patients enrolled and included in the analysis + selected patients missed by surveyors + refusals + withdrawals + patients lost to follow-up]/patients included in the analysis).

Descriptive analyses of patient and health worker-level indicators were performed with the SURVEYFREQ procedure, which uses the Taylor expansion method to account for cluster sampling and unequal analysis weights. Health worker indicators were not weighted, as selection probabilities were equal. Patient-level indicators were weighted, unless otherwise noted. As the sample size of health facilities (n = 33) was a relatively large proportion of all facilities in the sampling frame (N = 57), confidence intervals (CIs) for health facility-level indicators were adjusted with the finite population correction factor--i.e., for a proportion, *p*, the standard error = {square-root [*p*(1 - *p*)/n]} × {square-root [(N - n)/(N - 1)]}. If *p *= 0% or 100% (and thus the estimated standard error would be zero), CIs were estimated using the exact binomial method.

Logistic regression modeling was performed using the SURVEYLOGISTIC procedure, which also uses the Taylor expansion method to address clustering and weighting. All models were weighted. Variables with a univariate p-value < 0.15 were included in a multivariate model (the full model). Reduced models were tested by removing variables with a multivariate p-value > 0.10. Variables with a multivariate p-value > 0.10 were not removed if odds ratios of other variables changed by > 20%. To assess confounding by excluded variables (univariate p-value ≥ 0.15), we added them to the reduced model one at a time and retained them if odds ratios of other variables changed by > 20%. Hypothesis testing and CI estimation were done with an alpha of 0.05.

## Results

### Enrollment

Survey teams visited all 33 sampled health facilities as scheduled. In these facilities, 100 health workers performed outpatient consultations; 93 (93.0%) were interviewed, and seven left before interviews could be administered. No health worker refused to participate. Of the 93 workers interviewed, 64 treated at least one included patient; and of the seven workers missed, six treated at least one included patient. Thus, patient-level indicators of case-management quality reflected the practices of 70 health workers (64 interviewed, six missed).

Altogether, 778 patients came for initial consultations; 389 of whom were selected for inclusion. Of these 389 patients, 177 were included, 170 were missed (i.e., no surveyor was available to begin the "follow the patient" procedure), 38 refused, three withdrew after enrollment, and one patient left the facility before completing all survey steps. Although many selected patients were not surveyed (i.e., 170 missed + 38 refusals + three withdrawals + one lost, or 212 patients), most (170/212, or 80.2%) were missed patients. These missed patients were unlikely to have been much different from included patients, as they were essentially missing at random. If missed patients are excluded, the "participation rate" (i.e., the proportion of patients approached for consent who were included) was 80.8% (177 included/[177 + 38 refusals + three withdrawals + one lost]).

### Health facility characteristics

Of the 33 facilities, five (15.2%) were hospitals and 28 (84.8%) were health centers. A very large number of health workers (3564) was estimated in the 57 eligible facilities (all health workers assigned to the facilities, including all departments and shifts). A median of five nurses per facility had AL training. All facilities had at least one nurse trained to use AL and RDTs.

Among the 33 sampled facilities, 1,103 patients sought care on the day of the survey visit: 778 (70.5%) initial consultations and 325 (29.5%) follow-up visits. The median numbers of total and initial consultations/facility/day were 26 (range: 12-119) and 18 (range: 5-77), respectively.

Nearly all facilities had thermometers and scales; but only half had AL algorithms (Table 1). All performed malaria testing. All facilities had AL in stock, and most (72.7%) had all four AL blister packs. However, two-thirds of facilities had AL stock-outs in the preceding three months, and most lacked quinine.

**Table 1:** Availability of equipment, staff, and medicines needed to manage malaria and other febrile illnesses in outpatient health facilities, Huambo Province, Angola

Characteristic	Health facilities with the characteristic (N = 33)	
	
	No.	% (95% CI)
Thermometer	28	84.8 (76.8-92.9)
Functional scale for weighing children	32	97.0 (93.1-100)
Booklet or chart with AL treatment algorithms for children and adults	16	48.5 (37.3-59.6)
Staff trained to perform microscopy	19	57.6 (46.5-68.6)
Functional microscope, according to the laboratory technician	19	57.6 (46.5-68.6)
Staff trained to perform RDTs	29	87.9 (80.6-95.2)
At least 25 valid (not expired) RDTs in stock	31	93.9 (88.6-99.3)
Malaria testing, by microscopy or RDT^a^	33	100 (89.4-100)
Malaria testing, by both microscopy and RDTs^b^	13	39.4 (28.5-50.3)
Mosquito bed nets for distribution to patients^c^	0	0 (0-10.9)
		
*Medicines in stock on the day of the survey visit*		
AL blister packs for patients 5-14 kg	26	78.8 (69.7-87.9)
AL blister packs for patients 15-24 kg	33	100 (89.4-100)
AL blister packs for patients 25-34 kg	33	100 (89.4-100)
AL blister packs for patients ≥ 35 kg	30	90.9 (84.5-97.3)
All four AL blister packs	24	72.7 (62.8-82.7)
Amodiaquine tablets	29	87.9 (80.6-95.2)
Quinine tablets	12	36.4 (25.6-47.1)
Quinine or quinidine (injectable)	18	54.4 (43.4-65.7)
Oral antibiotic (amoxicillin, ampicillin, cotrimoxazole, or erythromycin)	31	93.9 (88.6-99.3)
Iron tablets	27	81.8 (73.2-90.4)
		
*Medicines in stock every day for the 3 months before the survey visit*		
AL blister packs for patients 5-14 kg	7	21.2 (12.1-30.3)
AL blister packs for patients 15-24 kg	12	36.4 (25.6-47.1)
AL blister packs for patients 25-34 kg	12	36.4 (25.6-47.1)
AL blister packs for patients ≥ 35 kg	10	30.3 (20.0-40.6)
All four AL blister packs	4	12.1 (4.8-19.4)

AL = artemether-lumefantrine; CI = confidence interval; RDT = rapid diagnostic test for malaria.

^a ^Malaria testing was considered available if, on the day of the survey visit, the health facility had either: 1) a microscopist and a functional microscope, or 2) staff trained to perform RDTs and at least 25 valid (not expired) RDTs.

^b ^On the day of the survey visit, the health facility had both of the following: 1) a microscopist and a functional microscope, and 2) a staff person trained to perform RDTs and at least 25 valid (not expired) RDTs.

^c ^N = 32 health facilities, one value missing.

### Health worker characteristics

Among the 93 interviewed health workers, the median age was 36 years (range: 21-70), and 51.6% (48/93) were female. Nearly all (91/93, or 97.8%) were nurses; two (2.2%) were physicians. Nurses had 2-5 years of pre-service medical training. Sixty percent of interviewed health workers had formal AL training, and two-thirds had informal AL training (Table 2). Most formal training courses (89.3%) covered RDT use, training duration was usually three days (range: 1-15), and most (75.0%) occurred in 2007.

**Table 2:** Health worker training and supervision in outpatient health facilities, Huambo Province, Angola

Characteristic	Health workers with the characteristic (N = 93)	
	
	No.	% (95% CI)
Health worker received at least one formal training on AL use (i.e., an organized course in a classroom or clinical setting that lasts at least a half day)	56	60.2 (47.7-72.8)
Health worker received informal training on AL use (i.e., a short, e.g., 1 hour, impromptu educational session provided by a supervisor or peer)	60	64.5 (53.1-75.9)
Health worker received formal or informal training on AL use	72	77.4 (66.6-88.2)
Health worker received at least 1 supervision visit in past 6 months (any supervision, even if unrelated to malaria)	68	73.1 (63.9-82.4)
Health worker received at least 1 supervision visit in past 6 months in which the supervisor observed and provided feedback on a consultation	32	34.4 (25.3-43.5)
Health worker supervised at least once in past 6 months on AL use	47	50.5 (40.4-60.7)

AL = artemether-lumefantrine; CI = confidence interval.

Three-quarters of health workers were supervised in the preceding six months (median = 1 visit, range: 0-5), but only one-third reported that supervision included observation and feedback on a consultation. Half of workers reported supervision on AL use; and of these, almost half (20/47, or 42.6%) reported never having received supervision with observation and feedback on a consultation. These results reflected the plan to have AL-related supervision in the first year of AL scale-up focus on pharmaceutical management.

Health worker caseloads (all consultation types combined) ranged from 1-44 patients/day (median = 13), and 16.3% (15/92 [1 missing]) had high caseloads of ≥ 25 patients/day. A knowledge assessment revealed that no health worker knew the complete description of which patients should be tested for malaria--even though workers were told they could consult reference or training materials during the interview. However, two-thirds (59/90, or 65.6%) of health workers knew fever was a criterion for testing.

Responses to case scenarios of hypothetical patients revealed several patterns. First, in three scenarios describing adults with fever and a negative test (RDT or microscopy), most health workers (72.0-81.7%) seemed to ignore the test and gave an incorrect diagnosis of malaria or suspected malaria. For nearly all (96-100%) case scenario patients that health workers diagnosed with malaria or suspected malaria, workers said they would treat with an anti-malarial. Second, in a scenario of an adult with fever, convulsions, and a positive blood smear, only two-thirds of workers (63/93, or 67.7%) correctly diagnosed severe malaria; but correctly diagnosed cases were usually (53/63, or 84.1%) treated with an injectable anti-malarial, and nearly all (87/93, or 93.5%) were referred for hospitalization. Third, in three scenarios of patients with symptoms suggestive of malaria, most workers (76.3-87.1%) correctly responded that they would test the patient. Finally, summarizing knowledge across all seven scenarios, the median percentage of questions correctly answered per health worker was 56.3% (range: 31.3-87.5%).

### Patient characteristics: demographics, consultation attributes, and illnesses

Patient ages ranged from 0-80 years (median = 8), and 72 (45.0%) were under-5s. Ninety-nine (55.9%) patients were female, and five (2.2%) reported being pregnant. Half (53.9%) of patients were seen by health workers with formal AL training, and 75.3% of patients were seen by workers with any AL training (formal or informal). Only 30.8% of patients sought care on the day of illness onset or the next day.

The chief complaint of half (49.0%) of patients was fever or malaria, and 119 (70.5%) had a febrile illness (fever by history or temperature > 37.5°C). Among these 119 patients, only 69.5% gave a chief complaint of fever or malaria (85.8% for under-5s and 54.9% for patients ≥ 5 years old). These results show why health workers must ask about fever and measure temperatures for all patients; solely relying on chief complaints could lead to many missed cases of febrile illness.

As defined in Huambo, 136 (77.8%) patients had suspected malaria (fever or three non-fever symptoms), half (48.0%) of whom had signs of a non-malaria illness--most commonly, respiratory infections (Table 3). Of 62 under-5s with suspected malaria, 58 (93.5% [unweighted]) would have been detected by fever history or measured temperature > 37.5°C alone. That is, if suspected malaria had been defined as "fever history or temperature > 37.5°C", the inclusion of "three non-fever symptoms" added little benefit (only four more patients, an additional 6.9% [4/58, unweighted]). For older patients, the corresponding benefit was somewhat larger: an additional 21.3% (13/61, unweighted).

**Table 3:** Patient characteristics in outpatient health facilities, Huambo Province, Angola

Characteristic	No. and weighted percentage of patients	
	
	n/N	% (95% CI)
*Patient had suspected malaria^a^*		
Among all patients	136/177	77.8 (69.5-86.2)
Among patients < 5 years old	62/72	79.5 (65.3-93.7)
Among patients ≥ 5 years old	74/105	76.5 (66.1-86.9)
Patient (all ages) with suspected malaria had symptoms of a non-malaria cause of the febrile illness (e.g., a respiratory infection)	63/136	48.0 (36.8-59.3)
		
*Malaria diagnoses according to the analysis algorithm (Figure 1)*		
Complicated malaria	1/177	0.8 (0-2.4)
Uncomplicated malaria	58/177	35.0 (26.0-44.1)
Not malaria	118/177	64.2 (55.1-73.3)
		
Patient was tested for malaria with either microscopy or a rapid diagnostic test, whether or not indicated by the policy	64/177	28.5 (17.9-39.2)
		
*Appropriateness of malaria testing*		
Patient with suspected malaria (i.e., test needed) was tested^b^	58/136	30.7 (17.9-43.6)
Patient without suspected malaria (i.e., no test needed) was not tested	35/41	79.2 (60.5-97.9)
Overall adherence to the testing policy (all ages)^c^	93/177	41.5 (30.2-52.7)
Overall adherence to the testing policy (age < 5 years old)^c^	36/72	40.0 (25.0-55.0)
Overall adherence to the testing policy (age ≥ 5 years old)^c^	57/105	42.7 (27.7-57.6)

CI = confidence interval.

^a ^Defined as either fever (history of fever or axillary temperature > 37.5°C), or at least 3 of the following: headache, joint pain, chills, sweating, anaemia (palmor pallor), cough (applies to children only), anorexia, fatigue, vomiting, or diarrhoea.

^b ^Microscopy and malaria rapid diagnostic tests were considered as equally appropriate tests.

^c ^Patients needing testing got tested, and patients not needing testing did not get tested.

According to the analysis algorithm, one patient (0.8%) had complicated malaria, 58 (35.0%) had uncomplicated malaria, and 118 (64.2%) had no malaria (Table 3). Proportions were similar for under-5s and older patients. Of the 59 malaria cases, 17 had a positive malaria test (based on testing performed by the observed health facility staff--not by surveyors); and the other 42 had suspected malaria, were not tested, and had no other (non-malaria) cause of fever identified (see Figure 1, footnote 7).

### Patient characteristics: quality of clinical assessment and use of diagnostic testing

Clinical assessments were evaluated by estimating the proportion of patients for whom health workers had determined if a given sign or symptom was present. "Determined" meant the worker was exposed to the information by any means (e.g., spontaneously offered by the patient, provided by the patient in response to a question, or obviously evident). This approach avoids penalizing health workers who do not ask for symptoms when it is not necessary. Fever history was determined for 87.6% of patients. However, temperatures were measured in only 25.9% of consultations, and assessment quality was poor for all other symptoms needed to identify suspected malaria. Symptom-specific proportions ranged from 1.8% (fatigue) to 39.2% (headache). A sub-analysis for patients without fever showed similarly poor quality (details available on-line) (see Additional file 1).

The pre-September 2007 policy recommended testing all patients with suspected malaria with microscopy or RDTs. Thus, 136 (77.8%) patients needed testing (Table 3). Only 30.7% of patients needing testing were tested, 79.2% of patients not needing testing were not tested (i.e., little over-testing), and overall adherence to the policy was 41.5%. Results did not vary by age.

Univariate statistical modeling identified several factors that were positively associated with malaria testing among patients needing testing: increasing supervision on AL use, lower caseload (< 25 versus ≥ 25 patients), higher patient temperature, and facility type (health centers versus hospitals) (details available on-line) (see Additional file 1). Two factors not associated with testing had p-values low enough (p < 0.15) to retain in the multivariable model: any AL training (formal or informal) and health worker age. The following were not associated with testing and not retained in the model: formal AL training, days of AL training, health worker knowledge score and sex, chief complaint of fever or malaria, and patient age and sex.

Multivariable modeling identified two factors significantly associated with testing (Table 4). First, the odds of testing among patients (needing testing) seen by health workers with caseloads < 25 patients/day were 18-fold greater than for those seen by workers with higher caseloads. Based on unadjusted results, the proportion of patients tested by health workers with lower and higher caseloads were 49.0% and 7.5%, respectively--a large difference of 41.5 percentage points. Second, the odds of testing increased by 2.5-fold for each increase in patient temperature by 1°C. Based on predicted probabilities from the reduced model, for each 1°C increase in temperature (in the 37-39°C range), the proportion of patients tested increased by about 13-22 percentage points. The multivariable model also revealed that the association between testing and any AL training (formal or informal) was of borderline statistical significance (odds ratio = 5.4; p = 0.072). Based on unadjusted results, the proportion of patients tested by health workers with any AL training and no AL training were 38.1% and 17.2%, respectively--a moderate difference of 20.9 percentage points.

**Table 4:** Predictors^a ^of correct malaria testing among patients with suspected malaria^b ^in outpatient health facilities, Huambo Province, Angola

Attribute	No. of patients tested/no. of patients who needed testing (weighted %)	Multivariate odds ratio (95% confidence interval)	p-value
Health worker's caseload (all patients) on the day of the survey visit			
0-24 patients	53/103 (49.0)	18.4 (6.8-49.6)	< 0.0001
25-43 patients	3/27 (7.5)	reference	
			
Patient's temperature measured by surveyor (N = 135; 1 missing value)			
39.0-39.9°C	9/11 (80.5%)	odds ratio per 1°C	0.0073
38.0-38.9°C	3/9 (29.9%)	increase in measured	
37.0-37.9°C	19/37 (30.2%)	temperature:	
36.0-36.9°C	25/70 (26.2%)	2.5 (1.3-5.0)	
35.0-35.9°C	1/8 (6.0%)		
			
Health worker training on case-management policy recommending AL and diagnostic testing			
Any training (formal or informal training)	48/99 (38.1%)	5.4 (0.9-33.5)	0.072
Not trained	9/32 (17.2%)	reference	

AL = artemether-lumefantrine.

^a ^This table presents results from the reduced model, which only included variables with multivariable p-values < 0.10. The full model, which included all variables with a univariate p-value < 0.15, also included health facility type (hospital versus health center), health worker supervised on AL use in the past 6 months, and health worker's age. In the full model, none of these factors had statistically significant associations with malaria testing (p-values ranged from 0.12 to 0.99). Both the full and reduced models were based on analyses of 129 patients because of missing values of predictors. The r-squared value for the full and reduced models were 94.9% and 94.4%, respectively.

^b ^In this analysis, all the patients needed testing by either microscopy or a rapid diagnostic test.

### Patient characteristics: results of malaria testing and quality of diagnosis

Among the 69 non-pregnant patients ≥ 5 years old with suspected malaria tested by surveyors with microscopy, 2 (3.4%; 95% CI: 0-8.5) were parasitemic with *P. falciparum*. Health workers tested 64 patients, 62 of whom had results available the same day and were included in subsequent analyses. Of these 62 patients, 50 were tested only with RDTs, 9 with smears only, and 3 with both an RDT and smear. Seventeen (27.4%) of these 62 patients tested positive, and results were similar for under-5s (8/30, or 26.7%) and patients ≥ 5 years old (9/32, or 28.1%).

Among the 27 patients tested by surveyors with microscopy (the gold standard for evaluating diagnostics) and by health workers (with RDT or microscopy), the sensitivity of health worker testing was 2/2, and the specificity was 19/25 (76.0%, unweighted). Results for health worker RDTs only were similar (sensitivity = 2/2, specificity= 17/22, or 77.3%, unweighted).

With the analysis algorithm as the standard, 66.1% of health workers' malaria-related diagnoses were correct, 20.1% were minor errors, and 13.9% were major (potentially life-threatening) errors (Table 5). With survey microscopy as the standard: a) for the one microscopy-positive case of uncomplicated malaria, the health worker's diagnosis was uncomplicated malaria (correct); b) for the one microscopy-positive case of complicated malaria, the worker's diagnosis was uncomplicated malaria (major error); and c) for the 70 microscopy-negative cases, workers correctly diagnosed no malaria in 34 cases (48.6%, unweighted) and incorrectly over-diagnosed malaria in 36 cases (51.4%, unweighted).

**Table 5:** Quality of malaria diagnosis in outpatient health facilities, Huambo Province, Angola

Characteristic and patient sub-group		No. and weighted percentage of patients		
	
		n	%	(95% CI)
*Health worker's malaria-related diagnosis for the 1 patient with a gold standard diagnosis^a ^of complicated malaria*				
Uncomplicated malaria (major error)		1	100	(NC)
			
*Health workers' malaria-related diagnoses for the 58 patients with a gold standard diagnosis^a ^of uncomplicated malaria*				
Complicated malaria (minor error)		2	2.6	(0-6.4)
Uncomplicated malaria (correct)		32	60.1	(44.5-75.7)
No malaria^b ^(major error)		24	37.4	(21.6-53.1)
			
*Health workers' malaria-related diagnoses for the 118 patients with a gold standard diagnosis^a ^of no malaria*				
Uncomplicated malaria (minor error)		40	29.9	(17.6-42.2)
No malaria (correct)		78	70.1	(57.8-82.4)
			
*Overall quality of health workers' malaria-related diagnoses among all 177 patients*				
Correct (health worker diagnoses of malaria and no malaria matched gold standard diagnoses)		110	66.1	(58.3-73.8)
Minor error (health worker incorrectly "over-diagnosed" uncomplicated malaria as complicated malaria, or over-diagnosed no malaria as uncomplicated malaria)		42	20.1	(11.4-28.7)
Major error (health worker incorrectly "under-diagnosed" complicated malaria as uncomplicated malaria, or under-diagnosed uncomplicated malaria as no malaria)		25	13.9	(8.0-19.7)

CI = confidence interval; NC = not calculated.

^a ^The "gold standard" malaria diagnosis (against which health worker diagnoses were compared) was defined by applying the analysis algorithm (Figure 1) to patient clinical signs and symptoms (assessed by surveyors, but information that should have been available to observed health workers) and laboratory data available to observed health workers (i.e., not the survey team's laboratory results).

^b ^Health workers' diagnoses of the 24 patients were gastrointestinal illnesses (n = 10; e.g., gastritis, intestinal parasites, and dysentery), respiratory illnesses (n = 5; e.g., bronchitis), skin problems (n = 2; e.g., scabies and skin boils), and other (n = 7; e.g., dental caries, trauma, chicken pox, and malnutrition).

### Laboratory assessment

Twenty-two staff from a convenience sample of 20 facilities were assessed as they performed RDTs. All the correct steps (one drop of blood and six drops of buffer solution) were followed in only half (11/22) of observations. The most common error was using too little buffer solution (two to five drops). The amount of blood was usually correct. Most staff knew that one should wait 15 minutes or a little longer, although one-third might have thought that waiting times were < 15 minutes or were unsure. Additionally, in some facilities, test results were reported in batches instead of reporting individual results as they were ready. This practice caused unnecessarily long waiting times (≥ 1 hour) for some patients (RDT results should be available in < 30 minutes).

### Patient characteristics: quality of treatment and counseling

Among all 177 patients, 61.4% of prescribed malaria-related treatments were correct, 22.3% were minor errors, and 16.3% were major (potentially life-threatening) errors (Table 6). The most common errors were prescribing no anti-malarials for patients with uncomplicated malaria and prescribing AL for patients without malaria. Errors such as under-dosing AL and using ineffective anti-malarials were uncommon.

**Table 6:** Quality of malaria^a ^treatment in outpatient health facilities, Huambo Province, Angola

Characteristic and patient sub-group		No. and weighted percentage of patients		
	
		n	%	(95% CI)
*Quality of malaria treatment among all 177 patients^b^*				
Correct (recommended treatment)		105	61.4	(52.0-70.7)
Minor error		42	22.3	(12.3-32.3)
Major error		30	16.3	(10.2-22.4)
			
*Quality of malaria treatment among the 59 patients with malaria^b^*				
Correct (recommended treatment)		27	49.0	(33.5-64.5)
Minor error^c^		2	5.4	(0-13.2)
Major error^d^		30	45.6	(28.2-63.1)
			
*Quality of malaria treatment for the 59 malaria cases (quality in terms of antimalarials obtained by patients and patient recall of treatment instructions)*				
Patient left the health facility with the recommended anti-malarial and knowledge of how to administer the drug at home		17	27.1	(14.8-39.4)
Patient left the health facility with an adequate (but not recommended) anti-malarial and knowledge of how to administer the drug at home		2	5.4	(0-13.2)
Patient left the health facility without at least one of the following: an effective anti-malarial or adequate knowledge of how to administer the drug at home		40	67.5	(53.6-81.5)
			
*Quality of malaria treatment among the 118 patients without malaria^b^*				
Correct (recommended treatment)		78	68.3	(54.6-82.0)
Minor error		40	31.7	(18.0-45.4)

AL = artemether-lumefantrine; CI = confidence interval.

^a ^The "gold standard" malaria diagnosis (against which health worker treatments were compared) was defined by applying the analysis algorithm (Figure 1) to patient clinical signs and symptoms (assessed by surveyors, but information that should have been available to observed health workers) and laboratory data available to observed health workers (i.e., not the survey team's laboratory results).

^b ^Quality in terms of anti-malarials prescribed by health workers. No error means that patients received recommended treatment in exact accordance with guidelines (malaria cases treated with the recommended anti-malarial with the recommended dosage, and non-malaria cases received no anti-malarial treatment). Minor error means that malaria cases received non-recommended, but still life-saving, anti-malarial treatment (either an overdose of a recommended anti-malarial, or an adequate dose of a non-recommended anti-malarial); and non-malaria cases received unnecessary anti-malarial treatment that was unlikely to cause serious harm. Major error means that malaria cases did not receive life-saving treatment (no anti-malarial, an ineffective anti-malarial, or an under-dosed anti-malarial).

^c ^The 2 minor errors were: 1 case of uncomplicated malaria treated with correctly dosed quinine, and 1 case of uncomplicated malaria in a child < 5 kg treated with AL (with a dosage appropriate for a child 5-14 kg).

^d ^The 30 major errors were: 26 cases of uncomplicated malaria not treated with anti-malarials, 2 cases of uncomplicated malaria treated with ineffective anti-malarials (1 treated with amodiaquine, 1 treated with sulfadoxine-pyrimethamine), 1 case of uncomplicated malaria treated with underdosed AL, and 1 case of complicated malaria treated with underdosed AL.

Among the 59 patients with malaria, the quality of prescribed treatments was lower: only 49.0% were correct, 5.4% were minor errors, and 45.6% were major errors (Table 6). In an analysis of treatment quality from the patient's perspective (i.e., patient left the facility with the anti-malarial in hand and demonstrated knowledge on administering it at home), only 27.1% of patients received recommended care, 5.4% received adequate (but not recommended) care, and 67.5% received inadequate care.

An analysis of 62 patients prescribed AL (whether or not it was indicated) revealed that health workers almost always dosed AL correctly, but the quality of counseling was mixed (Table 7). Nearly all patients received the correct dose (95.1%) and complete dosing instructions (88.2%). However, only 60.9% of patients could repeat all the dosing instructions. Furthermore, few patients were given the first dose during the consultation (10.7%) or advised to take the medicine with food (31.3%).

**Table 7:** Use of AL: frequency of prescription, and appropriateness of dosing and counseling (whether or not AL was indicated, according to guidelines) in outpatient health facilities, Huambo Province, Angola

Characteristic and patient sub-group		No. and weighted percentage of patients		
	
		n	%	(95% CI)
AL prescribed (whether or not indicated, according to guidelines) among all 177 patients		62^a^	35.5	(24.9-46.2)
			
*Quality of dosing for the 62 patients who received AL*				
Correctly dosed		59	95.1	(89.2-100)
Underdosed		2	3.9	(51.4-88.4)
Overdosed		0	0	(NC)
AL not recommended (weight < 5 kg)		1	1.0	(0-3.1)
			
First AL dose given during consultation (for the 62 patients who received AL)		9	10.7	(1.2-20.3)
			
*Quality of counseling for the 62 patients who received AL*				
HW gave complete dosing instructions (definition of a dose, no. of doses/day, and treatment duration)^b^		55	88.2	(78.3-98.2)
HW advised to take the medicine with food		17	31.3	(12.3-50.2)
Patient could repeat all dosing instructions given by the HW (even if HW's dosage was incorrect)^c^		43	60.9	(44.2-77.6)
HW advised to take the medicine with milk or fat-containing food		4	4.9	(0-10.8)
HW advised to return for a follow-up visit		8	14.4	(0.4-28.3)
HW advised to sleep under a bed net to prevent malaria		0	0	(NC)
HW advised to return to the health facility if the patient becomes seriously ill		5	5.8	(0-11.8)
HW advised to complete all the treatment (take all medicines)		41	69.9	(51.4-88.4)

AL = artemether-lumefantrine; CI = confidence interval; HW = health worker; NC = not calculated.

^a ^Of these 62 patients, 59 actually had AL in hand; for the other 3 patients, AL had been prescribed but the medicines were not given.

^b ^Dosing instructions were considered complete even if the dosage was incorrect, although in nearly all cases (53 of the 55 patients) the dosage was correct.

^c ^Patient had to correctly repeat the definition of a dose, the number of doses per day, and the treatment duration. The response "*ate que termine*" (until all the medicines are done) was considered a correct response for treatment duration. The denominator of this statistic was 60 (2 missing values).

### Graphical pathway analysis

**Figure 4:**
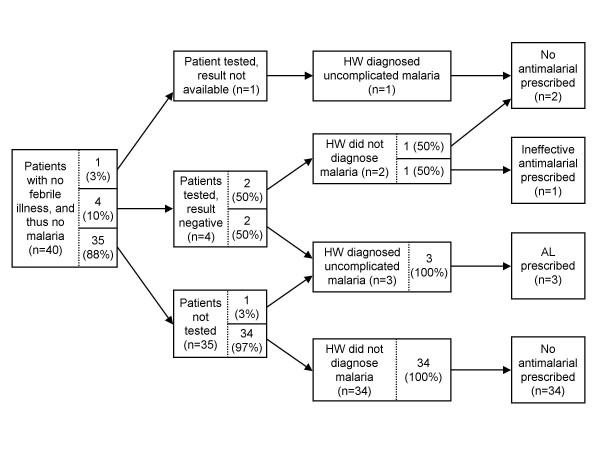
**Graphical pathway analysis of the case-management process for 40 patients without suspected malaria**. AL = artemether-lumefantrine; HW = health worker.

A graphical pathway analysis was performed to link results of individual steps of the case-management process and identify strengths and weaknesses of health worker practices. For simplicity, percentages are unweighted. Among the 40 patients without febrile illness/suspected malaria (and thus no malaria), we found that a large majority of patients (35/40, or 87.5%) were not tested for malaria, nearly all (34/35, or 97.1%) of these untested patients were not diagnosed with malaria; and of the 34 patients without a malaria diagnosis, none were treated with an anti-malarial (Figure 4, steps along the bottom of the figure). In other words, most patients were managed correctly at all points in the case-management process.

**Figure 5:**
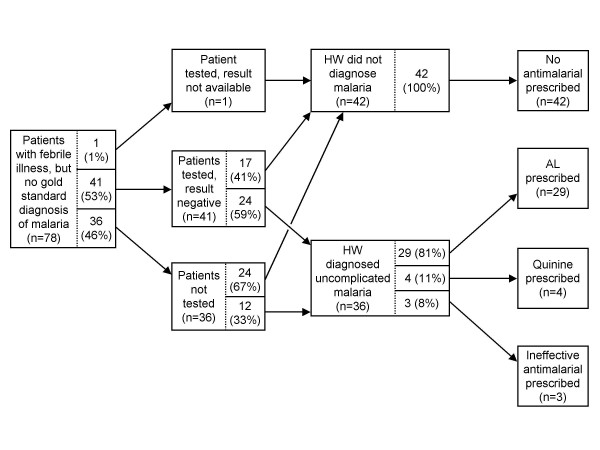
**Graphical pathway analysis of the case-management process for 78 patients with suspected malaria but no gold standard malaria diagnosis**. AL = artemether-lumefantrine; HW = health worker.

Among the 78 patients with febrile illness/suspected malaria but no gold standard malaria diagnosis, many (36/78, or 46.2%) were not tested, even though they should have been (Figure 5). Among the 41 patients with a negative test result, over half (24/41, or 58.5%) were diagnosed with malaria. Nearly all (33/36, or 91.7%) patients diagnosed with malaria were prescribed an effective anti-malarial; and of the 42 patients without a malaria diagnosis, none were treated with anti-malarials.

**Figure 6:**
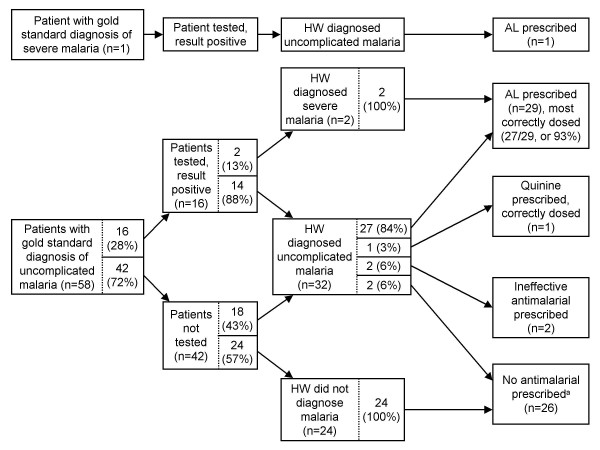
**Graphical pathway analysis of the case-management process for 59 patients with a gold standard malaria diagnosis**. AL = artemether-lumefantrine; HW = health worker. ^a ^None of these 26 patients had been tested for malaria, although all were seen at health facilities in which testing was available.

Among the 59 patients with febrile illness/suspected malaria and a gold standard malaria diagnosis, most (42/58, or 72.4%) were not tested, even though they should have been (Figure 6). Of 26 patients who did not receive anti-malarials (major error), none had been tested. All 17 patients with a positive malaria test were diagnosed with malaria. A large majority of patients (31/35, or 88.6%) diagnosed with malaria were prescribed effective anti-malarials; and of the 24 patients without a malaria diagnosis, none were treated with anti-malarials.

In summary, many patients who should have been tested were not, which led to many incorrect diagnoses. Health workers did not trust their negative test results: over half of test-negative patients were diagnosed with malaria (26/45, or 57.8%) and treated with anti-malarials (27/45, or 60.0%). Results were similar for microscopy and RDTs, but distrust was greater when the patient was ≥ 5 years old. Malaria was diagnosed in 73.9% (17/23) of test-negative patients ≥ 5 years old (versus 40.9% [9/22] for under-5s), and anti-malarials were prescribed for 73.9% (17/23) of test-negative patients ≥ 5 years old (versus 45.5% [10/22] for under-5s). Health workers did trust positive test results, as all test-positive patients were diagnosed with malaria. Prescribed treatments closely matched health worker diagnoses: among 73 patients whom workers diagnosed with uncomplicated malaria, a large majority (83.6%) received AL (usually correctly dosed); and among 102 patients without a diagnosis of malaria, nearly all (97.3%) received no anti-malarial treatment.

### Design effects and intraclass correlation coefficients

Design effects and intraclass correlation coefficients (*ρ*) were examined for 24 key patient-level indicators of case-management quality related to patient assessment, diagnosis, treatment, and counseling. Weighted design effects ranged from about 1.0 (no correlation) to 3.4 (moderate correlation); the median was 1.7. Weighted values of *ρ *ranged from just under zero to about 1.0; the median was 0.3. Design effects for the two indicators of correct malaria treatment were close to one (i.e., 1.1 and 1.6), which reflects relatively little correlation.

As heterogeneous analysis weights tend to increase design effects and decrease precision [19], an unweighted analysis was performed to quantify the effect of heterogeneous weights (final weights ranged from 1.7-40.6). As expected, unweighted design effects and *ρ *were usually lower than values from the weighted analysis. An examination of the ratio of weighted design effects to unweighted design effects revealed a median ratio of 1.48. In other words, heterogeneous weights typically increased design effects by 48%, which widened CIs by 22% (i.e., square-root [1.48] = 1.22) (details available on-line) (see Additional file 1).

## Discussion

This survey evaluated about 14 months of activities to scale-up Angola's ACT policy in a province with weak infrastructure that was the focus of a well-funded malaria control initiative. As the survey occurred in the middle of a multi-year implementation plan and as training and supervision have continued since the survey, the current situation is probably better than what is reflected in this report. However, the programmatic strengths and weaknesses we identified were notable, and the results reveal problems that might exist today. Moreover, as the setting is typical of many parts of the developing world, the broader lessons might be relevant to other low-income countries.

The first main finding was related to the fact that when a policy is ambiguous, overly complex, communicated imprecisely, or changed frequently, confusion can ripple throughout the health system. In Angola, in 2007, parts of the NMCP policy lacked clarity. In Huambo, local staff responded by adding details in training materials to fill gaps. However, despite these good intentions, the result was that nearly everyone involved in scale-up activities had a different interpretation of the guidelines. Additionally, the complex definition of suspected malaria likely contributed to the finding that no health worker knew the full definition. These observations underscore the importance of developing policies and training materials that are clearly written and as simple as possible. Donors and other development partners should insist that these fundamental programmatic building blocks be in place before large-scale implementation activities are undertaken. Furthermore, the statement that patients might have malaria even with a negative test, which appears in policy statements from Angola and other countries [5,20], should be replaced with more specific guidance. At the international level, experts can assist by helping to answer the question: precisely who should be treated when a test is negative? Without specific guidance, health workers are justified in ignoring negative test results; and if instances exist when test results can be ignored, some workers might conclude that testing has limited value and can be skipped.

The second finding was that many case-management elements were in place, although some additional strengthening was needed. All facilities had AL in-stock, the ability to perform malaria testing, and AL-trained health workers. Although scale-up was still ongoing, aspects of health facility preparedness requiring improvement during the survey were: oral and injectable quinine were frequently not in-stock, few facilities had all AL blister packs continuously in the previous three months, one-quarter of patients were seen by health workers without AL training, and supervision on AL use was uncommon and often did not involve observation and feedback. Some of these deficiencies have been observed elsewhere [9]. Additionally, the health worker knowledge assessment revealed two major gaps: no one knew the full definition of whom to test (although most could identify which patients needed testing in case scenarios), and most did not trust negative test results. These findings illustrate the importance of basic program management, in terms of stocking essential medicines, scheduling trained health workers to perform consultations, and providing clinical supervision as training occurs. Although it might not seem directly linked to lowering malaria mortality, strengthening managerial capacity should be a priority of today's well-funded disease control initiatives.

**Figure 7:**
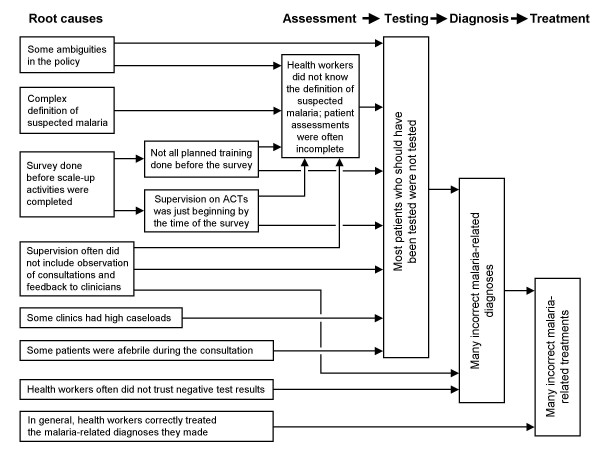
**Causal diagram of the case-management process in outpatient health facilities, Huambo Province, Angola**.

The third main finding was related to case-management quality. As many indicators are needed for a comprehensive description, we constructed a causal diagram to display the key practices and show the causal chain of observed problems (Figure 7). Clearly, this diagram is simplistic, as it omits many fundamental environmental factors of potential importance, such as low salaries, poor motivation, weak infrastructure, and perceived patient expectations. As illustrated at the top of Figure 7, the case-management process begins with patient assessment. Except for fever history, assessments were often incomplete. Other studies have found similar deficiencies [20,21].

Regarding malaria testing, the main problem was substantial under-use. We explored testing practices in-depth because testing and trusting the result was the key to making a correct diagnosis and correct diagnosis was the key to prescribing appropriate treatments. Interestingly, the factors significantly associated with testing were caseload and patient temperature (root causes on the left of Figure 7). Similar analyses from Tanzania [22] and Zambia [21] in the pre-ACT era found a variety of factors positively associated with testing: patient age ≥ 15 years, fever, fever history, and longer travel time to clinic. Testing was less likely if patients had a skin problem or single-etiology illness (e.g., respiratory infection alone). These results illustrate the importance of non-intervention environmental factors [23] and could be used to guide future quality improvement activities (e.g., changing schedules to reduce high caseloads, and teaching health workers to test all patients with fever history, not just those with elevated temperatures). AL training had a borderline association with testing; but even ignoring statistical significance, the absolute proportion of patients tested by AL-trained workers was low (38.1%). Supervision on AL was not associated with testing, perhaps because it focused on pharmaceutical management rather than clinical practice. It was notable that health worker knowledge was not associated with correct testing, which might explain the modest effect of training. The lack of association between health worker knowledge and practice has been observed in other settings [24-26], and this phenomenon should make program managers consider other quality improvement strategies besides training--such as incentives, job aids, and quality management principles to name a few [23].

The patterns of diagnosis and treatment quality were similar to those of testing. Only half to two-thirds of patients were correctly diagnosed and treated. The two most common diagnostic errors were missing malaria cases and over-diagnosing malaria among patients without it. Over-diagnosis can be partly explained by workers' apparent distrust of negative test results, which echoes results of the knowledge assessment. Erroneous diagnoses translated into inappropriate treatments because, in general, workers correctly treated the diagnoses they made. Additionally, health workers rarely administered the first dose of AL during the consultation (perhaps because no food was available), and many aspects of counseling needed improvement--problems found in other settings [4,9]. An analysis from the patient's perspective, which was perhaps the most relevant quality indicator (i.e., patients needing treatment left the facility with the correct medicine and knowledge on administering it at home), found that only about one-third of patients received adequate (i.e., life-saving) care.

These results point to what might be the most important lesson of our evaluation and other similar studies [3-9]: commonly-used scale-up strategies such as providing commodities, cascade training, job aids, and a little supervision of uncertain quality, seem to have only a moderate effect. Moreover, implementation of these strategies is time-consuming. For malaria control initiatives to achieve better results faster, new approaches are needed.

There were several important positive findings related to treatment. For example, it was reassuring that nearly all patients that health workers diagnosed with malaria were prescribed AL at the correct dose and provided with accurate dosing instructions. Also, there was little use of ineffective and non-recommended anti-malarials--a problem observed elsewhere [3,6,27].

### Methodological lessons

This survey provided several methodological lessons. First, it was challenging to develop the definition of the gold standard against which case-management quality should be evaluated. Should the definition be based on a draft policy document; verbal explanations of NMCP officials; or what was taught in training courses? Ultimately, two parallel analyses were performed to take the perspective of what had been taught to health workers (presented in this report), and then, as the policy was evolving, what NMCP officials wanted the policy to be (details available on-line) (see Additional file 1).

The second lesson concerned the "follow the patient" sampling method. This method allows surveyors to observe all parts of the patient's clinic visit and might reduce loss after enrollment. However, clearly, many patients were missed because of an insufficient number of surveyors and because health workers did not always see patients in the order they arrived at the facility (e.g., if patient 1 was selected for inclusion but not immediately seen by a health worker, a surveyor had to wait for patient 1's consultation, during which time other sampled patients might be seen by health workers). Also, the method led to widely varying analysis weights, which reduced precision. Another approach, which is probably better, is to have surveyors at fixed stations (e.g., observers spend the entire visit observing consultations). The disadvantage of the fixed-station method is that a small survey team might miss consultations in facilities with several consultation rooms. The solution is to either randomly select consultation rooms (and record selection probabilities, so results can be weighted) or determine in advance the number of consultation rooms in each sampled facility and create a small pool of extra surveyors to work at the larger facilities.

Finally, this survey demonstrated the positive bias that can occur when quality is evaluated only by measuring how well prescriptions match health worker diagnoses. With this approach, quality seemed excellent. Most (83.6%) patients whom workers diagnosed with malaria received AL, and nearly all (97.3%) patients whom workers did not diagnose with malaria were not prescribed anti-malarials. In contrast, when quality was assessed with a gold standard based on expert re-examination, results were much lower.

### Limitations

The survey has several important limitations. First, it only included facilities in which ACT implementation activities had occurred. Therefore, results cannot be generalized to facilities where many patients might seek care--especially government-run health posts and private-sector facilities. Second, the response rate was low because surveyors missed eligible patients, although it seems unlikely that missing these patients introduced a large bias. Third, directly observing consultations could have caused health workers to be more careful than usual [28,29], thus overestimating case-management quality somewhat. Fourth, some patient groups with suspected malaria were not tested. This issue, along with the survey period occurring before the peak malaria season (the rains began late), led to a patient sample size that was too small to evaluate the performance characteristics of microscopy and RDTs adequately. Moreover, by missing the peak malaria season, the parasite prevalence might not be a reliable estimate for characterizing the level of malaria transmission intensity in the province. Lastly, there was some ambiguity in the gold standard diagnosis and treatment for cases in which health workers did not test patients who should have been tested or vice-versa, although the survey's general conclusions were unlikely to have been affected.

## Conclusion

Huambo Province is a challenging place to scale-up a new case-management policy that recommends a new drug, emphasizes diagnostic testing, and introduces a new test. As of late-2007, the scale-up process was well underway, and the survey had important positive findings. However, key gaps were found. In particular, the ambiguous policy, under-use of malaria testing, and distrust of negative test results led to many incorrect malaria diagnoses and treatments. In 2009, Angola published a policy that clarified many issues. As problems identified in the survey are not unique to Angola, better strategies for improving health worker adherence to guidelines are urgently needed. Surveys that assess case-management quality with observations of consultations can provide crucial information for developing local recommendations and guiding scale-up elsewhere.

## Abbreviations

ACT: artemisinin-based combination therapy; AL: artemether-lumefantrine (i.e., Coartem®); CI: confidence interval; NMCP: National Malaria Control Programme; RDT: rapid diagnostic test; under-5: under 5 years old; and WHO: World Health Organization.

## Competing interests

The authors declare that they have no competing interests.

## Authors' contributions

AKR, GFP, JM, ACFSS, and NPM designed the survey; AKR, GFP, JM, ACFSS, NPM, and PVD supervised data collection in the field; AKR and GFP conducted the analysis; AKR drafted the manuscript, which all authors edited and approved.

## Supplementary Material

Additional file 1 **Full survey report**. The complete report of the health facility survey in Huambo Province, Angola, 2007. Click here for file
