# Supplementary material for: Quality of malaria case management at outpatient health facilities in Angola
Source: Malar J. 2009 Dec 2;8:275. doi: 10.1186/1475-2875-8-275 (PMC2795764; doi:10.1186/1475-2875-8-275)
Supplement: Additional file 1 — Full survey report. The complete report of the health facility survey in Huambo Province, Angola, 2007. [file 1475-2875-8-275-S1.PDF]

A survey to evaluate the quality of malaria case management in outpatient health facilities, Huambo Province, Angola, October–November, 2007

Final report

Alexander K. Rowe
Malaria Branch, Division of Parasitic Diseases, Centers for Disease Control and Prevention (CDC), Atlanta, United States

Gabriel F. Ponce de León
Malaria Branch, Division of Parasitic Diseases, CDC, Atlanta, United States

Jules Mihigo
Malaria Branch, Division of Parasitic Diseases, CDC, Luanda, Angola

Ana Carolina F. S. Santelli
National Malaria Program Office, Secretariat for Surveillance in Health, Ministry of Health, Brasilia, Brazil

Nathan P. Miller
The MENTOR Initiative, Huambo, Angola

Pedro Van-Dunem
Angola National Malaria Control Program, Ministry of Health, Luanda, Angola

ACKNOWLEDGEMENTS

We thank Alexandre Macedo de Oliveira (CDC) for his assistance in developing the protocol, Filomeno Fortes (Angola National Malaria Control Program) for his general support of our survey, the staff of the Essential Health Services Project of Chemonics International Incorporated for administrative and logistical support, the staff of the MENTOR Initiative for additional administrative and logistical support, and Alicia Swann (CDC) for her careful and detailed assistance with data management. We give special thanks to Zephirin Meya Mpambu (World Health Organization) for his excellent assistance with developing the protocol, training surveyors, supervising data collection, and advising on the analysis plan.

FUNDING

This survey was funded by the United States President's Malaria Initiative.

TABLE OF CONTENTS

	Page
Abbreviations ……………………………………………………………………………..	4
Executive summary ……………………………………………………………………	5
Introduction ……………………………………………………………………………	9
Objectives ……………………………………………………………………………...	11
Methods ………………………………………………………………………………...	11
Results …………………………………………………………………………………	19
Discussion ……………………………………………………………………………...	32
References ……………………………………………………………………………...	41
Text boxes ……………………………………………………………………………...	44
Tables …………………………………………………………………………………..	47
Figures …………………………………………………………………………………	102
Annexes ………………………………………………………………………………...	111

ABBREVIATIONS

%-points	Percentage-points
ACT	Artemisinin-based combination therapy
AL	Artemether-lumefantrine (i.e., Coartem®)
CDC	Centers for Disease Control and Prevention
CI	Confidence interval
IMCI	Integrated Management of Childhood Illness
IQR	25–75% interquartile range
IRLT	Interview, re-examination, and laboratory testing
NMCP	National Malaria Control Program
OR	Odds ratio
PMI	United States President's Malaria Initiative
RDT	Rapid diagnostic test
USAID	United States Agency for International Development
Under-5	Under 5 years old
WHO	World Health Organization

EXECUTIVE SUMMARY

Introduction

Angola's malaria case-management policy recommends treating uncomplicated malaria with artemether-lumefantrine (AL). In Huambo Province, in central Angola, large-scale implementation of the case-management policy began in August 2006 and included training health workers, delivering AL to health facilities, and improving malaria diagnostic testing (microscopy and rapid diagnostic tests [RDTs]), as well as some supervision and community education. Scale-up efforts were largely supported by the U.S. President's Malaria Initiative. Before September 2007, the national policy recommended that all patients with suspected malaria should be tested and that patients with a positive test should be treated with an antimalarial. In September 2007, the Director of the National Malaria Control Program announced a new policy that children under 5 years old with suspected malaria do not need testing and should be treated presumptively with an antimalarial.

The survey was conducted from October–November, 2007, about 14 months after implementation activities began. By the time of the survey, however, scale-up activities were still underway. Since the survey, a substantial amount of additional training, supervision, and other activities have been conducted. The timing of the survey allowed analyses that could both look back and evaluate how well the old policy had been implemented, and look forward to identify gaps and develop practical guidance on how the new policy could be implemented efficiently.

Objectives

Assess the readiness of health facilities to manage malaria, the quality of malaria case-management, and the quality of malaria laboratory testing.

Methods

We conducted a cross-sectional cluster survey in a statistically representative sample of outpatient health facilities in Huambo Province where the AL policy had been implemented. Surveyors spent one day at each of 33 selected facilities. Surveyors observed consultations, interviewed and re-examined patients, and performed a blood smear and RDT for non-pregnant patients ≥5 years old with suspected malaria. Also, health workers were interviewed, and an assessment was conducted to determine the availability of drugs and equipment. We measured indicators of AL availability, health worker training, supervision, health worker knowledge, use of malaria diagnostics, the quality of malaria diagnosis and treatment, and AL-related counseling. Unless stated otherwise, case-management quality was evaluated by comparing health worker assessments, diagnoses, and prescribed treatments against a "gold standard" that was based on surveyor interviews and re-examinations, and laboratory testing performed by health workers (i.e., the gold standard was based on information that should have been available to health workers). Separate gold standards were created to reflect the "old" policy (pre-September 2007) and "new" policy (announced in September 2007). Even though the survey was conducted after the new policy was announced, only three health workers had been trained in the new policy before survey; thus, virtually all surveyed health workers would have been expected

to follow the old policy. All analyses accounted for the cluster sample design; and unless otherwise stated, patient-level analyses were weighted to adjust for selection probabilities and non-response.

Results

Surveyors visited all 33 selected health facilities, and enrolled 93 health workers and 177 patients. Although many sampled patients were not included because surveyors were busy observing other patients, the loss of these patients was unlikely to have introduced a large bias. All health facilities had AL in stock, although two-thirds had stock-outs in the preceding 3 months. All facilities had ≥1 health worker with training on AL and RDTs (median of 5 trained nurses per health facility), however only 53.9% of patients were seen by a trained health worker. Half of health workers had supervision on AL use in the preceding 6 months.

An assessment of health workers' knowledge of the case-management policy showed that no health worker could repeat the complete definition of which patients needed testing, although 65.6% of health workers correctly responded that fever was a criterion. In response to three case-management scenarios, 76.3–87.1% of health workers correctly stated when malaria testing was needed. In three scenarios of adult patients with a febrile illness and a negative malaria test result (RDT or microscopy), 72.0–81.7% of health workers incorrectly diagnosed malaria, which suggested that health workers did not trust negative test results. In a scenario of severe febrile illness and a positive blood smear, 97.8% of health workers correctly diagnosed malaria.

We found that the assessment of fever history was generally very good, but the assessment quality was poor for all other symptoms needed for the case definition of suspected malaria. Malaria testing was greatly under-used, as only 30.7% of patients with suspected malaria who needed testing according to the old policy were tested. Statistical modeling revealed that health workers were significantly more likely to test patients needing a test if the health worker's caseload was <25 patients per day and if the patient had an elevated temperature at the time of the consultation. Patients were more likely to be tested by health workers who had received training on the AL policy, although the association was of borderline statistical significance and testing was still uncommon (38.1%) among health workers exposed to the AL training. Unnecessary testing was relatively uncommon. Of patients who did not need testing, only 20.8% were tested. Results were similar for an analysis using the new policy as a standard.

Regarding the accuracy of laboratory testing, our survey had too few patients to provide a precise answer. Among the 27 patients tested by surveyors with microscopy (our gold standard for evaluating malaria diagnostics) <u>and</u> by health workers (with RDT or microscopy), the sensitivity of health worker testing was 2/2, and the specificity was 19/25 (76.0%, unweighted).

According to the old policy gold standard, 0.8% of patients had complicated malaria and 35.0% had uncomplicated malaria; and according to the new policy, 4.3% had complicated malaria and 45.9% had uncomplicated malaria. According to the old policy, 66.1% of malaria-related diagnoses were correct, 20.1% were minor errors, and 13.9% were major (potentially life-threatening) errors. According to the new policy, the quality of diagnosis was somewhat lower:

61.8% of malaria-related diagnoses were correct, 15.0% were minor errors, and 23.2% were major errors.

Compared to the old policy gold standard, among all 177 patients, 61.4% of malaria-related treatments were correct, 22.3% were minor errors, and 16.3% were major (potentially life-threatening) errors; and among the 59 patients with malaria, treatment quality was lower: only 49.0% of malaria treatments were correct, 5.4% were minor errors, and 45.6% were major errors. According to the old policy, the most common errors among all patients were prescribing no antimalarials for patients with uncomplicated malaria and prescribing AL for patients without malaria. According to the new policy, treatment quality was somewhat lower than for the old policy.

Analyses that were not based on the gold standards revealed that, in general, health workers correctly treated the diagnoses they made. AL was prescribed to 83.6% of patients whom health workers diagnosed with uncomplicated malaria, and no antimalarial was prescribed to 97.3% of patients whom health workers did not diagnose with malaria. Additionally, an analysis of patients prescribed AL (whether or not AL was indicated by the guidelines) revealed that health workers almost always dosed AL correctly and gave accurate dosing instructions to patients; however, other aspects of counseling need improvement.

Among the 69 patients that surveyors tested with microscopy (non-pregnant patients ≥5 years old), only two (3.4%) were positive for *Plasmodium falciparum*. Two possible explanations are that the result was for a group of patients who typically are less likely to have malaria as a cause of febrile illness than children under 5 years old and pregnant women, and that the survey was conducted early in the rainy season, before malaria transmission had peaked. Still, low prevalence raises questions about the importance of malaria as a cause of illness in Huambo Province.

Conclusions

Implementation of the AL policy has clearly been started; and the NMCP, development partners, local implementers, and health facility staff should be congratulated. The survey had several important positive findings. All health facilities had AL in-stock, trained health workers, and the ability to perform malaria laboratory testing; most patients whom health workers diagnosed with malaria were prescribed AL at the correct dose; AL dosing instructions were accurate; and ineffective and non-recommended antimalarials were rarely used. However, important gaps were found. In particular, the unclear case-management policy, under-use of malaria testing, and distrust of negative test results led to many incorrect malaria diagnoses and inappropriately treated patients. The strengths and weaknesses identified in this evaluation directly led to practical recommendations, including the need for the development and dissemination of a clear policy and training materials, improved drug and staff management, increased adherence to clinical guidelines, strengthened laboratory diagnostic practices, and a follow-up evaluation to determine whether actions taken were successful. Lessons from this evaluation might apply to other parts of Angola and other low-income countries.

Recommendations

1) **Regarding policy.** Review the case-management policy document, clarify the guidelines, finalize and disseminate the document, and work with program and training experts to develop clear training materials. Revise AL dosing guidelines so weight categories have no gaps or overlaps (i.e., 5.0–14.9 kg, 15.0–24.9 kg, 25.0–34.0 kg, and >34.0 kg). Consider defining suspected malaria as simply a history of fever or a measured axillary temperature $\geq$37.5°C (i.e., drop the part of the definition that includes $\geq$3 non-fever symptoms).

2) **Regarding drug management.** Improve the drug management system to avoid stock-outs of antimalarials, especially for oral quinine and drugs for severe malaria (i.e., injectable quinine or artesunate, or artemether suppositories).

3) **Regarding staff management.** Schedule health workers with AL training to work on weekdays during regular hours, when many patients with suspected malaria are seen.

4) **Regarding training.** Implement the existing training plan (which now includes training on Integrated Management of Childhood Illness guidelines). Because of the importance of differential diagnosis, integrated training should continue to be supported. Training materials should be reviewed (and revised, if needed) to ensure that they are appropriate for the educational level of the health workers that will use them.

5) **Regarding supervision.** Evaluate case-management quality (recommendation 9) and use results to guide supervision plans. Supervision should include observation of consultations and constructive feedback, and focus on: complete patient assessments, identifying which patients need malaria testing, building trust in diagnostics so test-negative patients are not treated, identifying complicated malaria cases, and improving counseling. Health workers who perform well should be supervised at least once every three months, and workers with important deficiencies should be visited more often. Supervisors themselves should be supported to give them a high level of technical and interpersonal skills.

6) **Regarding guideline adherence.** Strengthen strategies to improve health worker adherence to guidelines, ideally in the context of a comprehensive approach. Along with supervision (recommendation 5), many interventions exist—e.g., job aids, incentives, targeted training, and the quality improvement process. If appropriate, seek external technical assistance.

7) **Regarding laboratory testing.** Precisely explain the national policy in a finalized document, use clear training on when to test and what to do with results (especially for negative results), emphasize differential diagnoses, establish a quality control system for RDTs and microscopy, continue supervision, implement strategies to keep health worker caseloads to <25 patients per day (e.g., by scheduling an extra health worker during busy times), and avoid reporting malaria test results in batches.

8) **Regarding care-seeking.** Implement a coordinated campaign with PMI, the MOH, provincial managers, WHO, and other partners to promote prompt care-seeking for a febrile illness.

9) **Regarding future evaluation.** Evaluate case-management quality, preferably on a recurrent basis, to monitor indicators on drug stocks, use of diagnostic tests, and case-management quality—perhaps with data collected by supervisors during their routine visits.

INTRODUCTION

In 2005, Angola was selected to receive funding from the United States President's Malaria Initiative (PMI). PMI's goal is to reduce malaria mortality by 50% by 2010 [PMI, 2009]. A key activity has been supporting the implementation of a malaria case-management policy in health facilities that recommends use of artemisinin-based combination therapy (ACT). This policy was based on World Health Organization (WHO) recommendations. The Angolan National Malaria Control Program (NMCP) has two first-line ACTs for the treatment of uncomplicated malaria cases: artemether-lumefantrine (AL) and amodiaquine-artesunate. The policy calls for ACTs and malaria testing with rapid diagnostic tests (RDTs) to be provided without charge for patients at public health facilities.

Huambo Province has been a PMI focus area. The province, in the highlands of central Angola (Figure 1), has meso-endemic, stable malaria transmission [COSEP et al., 2007]. The peak malaria transmission season extends from November–April (personal communication from Rachel Shaw, The MENTOR Initiative, January 23, 2009). *Plasmodium falciparum* is responsible for >90% of all infections. The primary vectors are *Anopheles gambiae* ss, *A. melas, and A. arabiensis* [COSEP et al., 2007]. The population of Huambo Province (2.3 million) is impoverished, and agriculture is a primary source of economic activity (Info-Angola, 2009). About half (45%) of the population lives in rural areas; and there is one large urban center, the city of Huambo. Angola suffered through a long civil war (1975–2002), and the city of Huambo was particularly hard hit. Many land mines and unexploded ordnance remain in Huambo Province. Since the end of the war, donor-supported efforts have rebuilt many health facilities.

PMI efforts began in five of the 11 municipalities (i.e., districts) in Huambo Province: Huambo, Bailundo, Caála, Londuimbali, and Tchikala Tcholohanga (heretofore referred to as the "initial five municipalities"). By the time of the survey, however, scale-up activities were occurring in all 11 municipalities. Scale-up activities were conducted by staff from the Ministry of Health, WHO, The MENTOR Initiative (a non-governmental organization), USAID, and CDC.

Four main activities have been undertaken in Huambo to implement the ACT policy: training health workers, providing ACTs and improving pharmaceutical management, strengthening diagnostics (microscopy and RDTs), and community education. Diagnostics are being strengthened to reduce unnecessary use of ACTs (which until recently had been relatively expensive), reduce avoidable adverse events and costs for patients, and slow the development of resistance to ACTs. As part of the PMI monitoring and evaluation plan, a health facility survey was conducted to evaluate the first three implementation activities listed above. Results are intended to document progress and identify lessons that could contribute to implementing the malaria case-management policy in Huambo and other provinces.

Implementation of the ACT policy

The ACT policy was adopted in October 2004, although as early as 2003 ACTs had been supplied and short trainings conducted in some health facilities in Huambo. In February 2006, the NMCP began disseminating the policy with a 5-day training course for 20 trainers: six from

Luanda (Angola's capital) and one from each of 14 other provinces, including Huambo. While a variety of trainings were held over the next several years, it is notable that there was no finalized policy document—only draft versions. Moreover, the draft policy document was somewhat unclear on malaria diagnosis. The document defined a malaria case as anyone with fever and a positive test for *Plasmodium*. It then had a "clinical case" sub-section that simply listed signs and symptoms of uncomplicated malaria (fever and eight other signs and symptoms) without any other explanation, and a "confirmed case" sub-section that gave a slight variation of the original case definition (<u>acute</u> febrile illness with a positive test for *Plasmodium*) [Angola NMCP, 2007]. The document then added that malaria cannot be ruled out in patients with a negative test and no other cause of fever, and that WHO recommends that malaria can be diagnosed among children <5 years old (under-5s) based on clinical findings without laboratory testing. Thus, while these statements provided general guidance on diagnosing malaria, they left some questions. For example: 1) should under-5s with fever be tested?; 2) how should non-fever signs and symptoms be used to decide which patients should be tested?; and 3) in patients with fever and a negative test, which non-malaria causes of fever should be ruled out before treating with an antimalarial?

In Huambo, in response to the unclear policy, local malaria control staff developed training materials that were based on the draft NMCP policy document, as well as WHO guidelines when the NMCP policy document was unclear. The training materials included flow diagrams to indicate more precisely what health workers should do during consultations (Figure 2). From August–September 2006, the training materials were used in 3-day courses for health workers from 16 health facilities. From January–November 2007, several related courses on the ACT policy were used to train 570 staff in all 11 municipalities, with some health workers attending more than one. Courses typically lasted 3 days (range: 1/2 day to 5 days), involved clinicians, pharmacists and sometimes laboratorians, and included instruction on RDT use (except for courses that only focused on pharmaceutical management). During this first phase of training, participants were expected to share the content of the training with health workers in their facilities (i.e., informal, cascade training[1]), although there was a multi-year plan to train additional health workers with the formal 3-day course (see the Discussion section). The need for a multi-year plan was partly based on the fact that the public sector employs an unusually large number of health workers[2].

In early September 2007, the Director of the NMCP modified the ACT policy: children under-5 with suspected malaria did not need testing and could be presumptively treated for malaria. The change was announced during a partners meeting; but, to the best of our knowledge, no policy document was revised to reflect the change. Moreover, one year after the announcement, partners sometimes still had different understandings of what presumptive treatment meant. Some thought that all under-5s with clinical signs of malaria should be treated with an antimalarial, and others thought that under-5s with clinical signs of malaria should only be treated with an antimalarial if no other cause of the febrile illness was found.

---

[1] In this report, formal training means an organized course (e.g., in a classroom or clinical setting) that typically lasts at least a half day, and informal training means a short (e.g., 1 hour) impromptu educational session provided by a supervisor or peer.

[2] As part of the peace agreement to end Angola's civil war, many nurses from the opposition faction (UNITA) were integrated into the health system.

AL, RDTs, and other commodities (e.g., quinine and sulfadoxine-pyrimethamine) were usually first supplied at the time of the trainings. Subsequently, the pharmaceutical management plan called for monthly deliveries to health facilities; but in practice, the time period varied. Commodities were sometimes delivered when the health facilities requested them and sometimes when the provincial authorities decided to deliver them. The timing of deliveries also depended on commodity availability. For several months before the survey, commodities were not supplied because of difficulties with transportation from the central warehouse in Luanda. However, commodities were delivered shortly before the survey. Regarding other activities to support the ACT policy before the survey, supervision on AL use and training on microscopy were just getting underway. Supervision had been done in the initial five municipalities (at least one visit per health facility, focused primarily on pharmaceutical management—not on the clinical use of AL and RDTs), and 41 laboratory technicians were trained in microscopy.

OBJECTIVES

In all 11 municipalities of Huambo Province, Angola, assess the following in outpatient health facilities where ACT implementation activities had occurred:

1. The quality of malaria case management for patients of all ages, from the perspectives of both the "old" policy (pre-September 2007) and "new" policy (announced in September 2007);

2. The preparedness of health facilities to manage malaria cases in terms of the availability of antimalarials, diagnostic testing, and trained staff; and

3. The quality of laboratory diagnostics (microscopy or RDTs) for non-pregnant patients $\geq$5 years old.

METHODS

Study design and population

We conducted a cross-sectional cluster survey from October 26 to November 21, 2007. This timing was intended to coincide with Angola's short rainy season; although in 2007, the rains, and thus malaria transmission, began late (around the last week of October). A cluster (i.e., the primary sampling unit) was a "health facility-day", which was defined as all patient consultations performed in an eligible health facility during regular working hours (Monday–Friday, 8am–3pm) during the 4-week survey period.

Health facilities were eligible for inclusion if they were a public or private facility[3] in Huambo Province that provided curative care in the outpatient setting and where the ACT policy had been implemented. "ACT implementation" meant that at least one health worker had been trained on the policy and that ACTs had been delivered at any time in the past. Health workers

[3] In fact, all eligible health facilities were public.

were eligible for inclusion if they performed consultations with sick patients in the outpatient setting of an eligible facility. Inclusion criteria for patient consultations were any initial consultation for a patient seeking care for any illness in the outpatient department of an eligible facility during regular working hours. Initial consultation meant the first time the patient had visited the facility for the current illness episode. Patients without illness were excluded (e.g., women seen for routine prenatal care, family planning clients, children brought for well baby checks or vaccinations, and patients seen for routine physical examinations). Thus, the populations of interest were all eligible health facilities, all health workers in the facilities, and initial outpatient consultations in the facilities during regular working hours during the 4-week survey period.

Sample size and sampling

To make the survey rapid and inexpensive, we chose a sample size of 30 health facilities, which is the smallest number of clusters that many experts advise for cluster surveys [WHO, 1991]. As health facility lists are sometimes outdated and Huambo Province has roads that become impassable during the rainy season, we assumed that about 10% of facilities would be permanently closed or inaccessible. Thus, the total sample size was 30 facilities + 10%, or 33 facilities. This sample was considered to provide acceptable precision[4].

We developed a sampling frame of health facilities by applying the inclusion criteria to a list provided by local officials and thereby identified 57 eligible facilities. We ordered the list by health facility type (hospitals, health centers with adequate infrastructure, health centers with low infrastructure, and health posts) and municipality and then selected a sample of 33 facilities using systematic sampling[5]. As we planned to visit each facility once, to select 33 "health facility-day" clusters, we used systematic sampling[6] to choose one date for each selected facility.

---

[4] Precision depends on: 1) whether the indicator is at the health facility, health worker, or patient level; 2) average number of patients per cluster; 3) indicator value; and 4) design effect (for patient indicators). For example, for the indicator "% of patients with malaria treated correctly," if there were 5 patients with malaria per health facility, 30 facilities, an indicator value of 50%, and a design effect of 3.8, then the precision (i.e., the 95% confidence interval half-width), D, would be +/- 15.6 %-points. D = square-root{$[(1.96)^2$ x (P) x (1 – P)] / (N / design effect)} = square-root{$[(1.96)^2$ x (0.5) x (1 – 0.5)] / ([30 facilities x 5 patients per facility] / 3.8)} = 0.156. In other words, the 95% confidence interval would be 50% +/- 15.6 (i.e., 34.4% – 65.6%).

[5] First, we classified health facilities into four types: hospital (n = 8), health center with adequate infrastructure (i.e., large outpatient facility with a laboratory and inpatient ward and where antenatal consultations are performed) (n = 23), health center with low infrastructure (i.e., large outpatient facility that lacks either a laboratory, inpatient ward, or antenatal consultations) (n = 25), or health post (i.e., small facility) (n = 1). Second, we created a list of health facilities that was ordered by health facility type; and within each type, we ordered municipalities (where facilities were located) alphabetically. Within facility-type-municipality groups, facilities were not ordered in any special way. Third, we numbered the ordered facility list (which became the facility identification number), calculated a skip interval (57 eligible facilities / sample size of 33 facilities, or 1.727), and selected a random starting point between one and the skip interval (starting point = 0.834). Fourth, we obtained the sample of 33 facilities by selecting the facility with the identification number closest to the random starting point, and selecting 32 other facilities by progressively adding the skip interval to the starting point and identifying facility identification numbers closest to this cumulative sum.

[6] We had two teams of surveyors. We mapped the 33 selected health facilities and divided them geographically into a group of 17 facilities that was approximately in the northern half of the province and a group of 16 facilities that

When survey teams arrived at health facilities, an attempt was made to include all initial patient consultations using the “follow-the-patient” approach (see below). However, in high-volume clinics, not all initial consultations could be included. The team reviewed patient registers, counted the number of patients seen in the past 5 weekdays, and used this number as an estimate of the caseload on the day of the survey visit. The estimated caseload was used to determine the sampling fraction of patients[7]. If the team determined that not all initial consultations could be included, then patients were selected with systematic sampling (e.g., every other patient or every third patient, with the first patient selected randomly[8]). Regarding the selection of health workers, surveyors observed health workers during the consultations of selected patients. Survey teams interviewed all health workers who performed sick patient consultations at the health facility on the day of the survey visit.

In summary, the above sampling plan resulted in a probability sample of health facilities, a non-probability sample of health workers, and a probability sample of initial patient consultations. Although this method did not give a probability sample of health workers, it probably was not a large source of bias (the sample probably under-represented health workers who worked relatively fewer days per week).

Data collection

Two survey teams visited health facilities and collected data, each consisting of three surveyors (one of whom also acted as a supervisor), a laboratory technician, and a driver. The surveyors and laboratory technicians were medical professionals. Additionally, there was a separate supervisory team, consisting of 1–2 survey organizers. Efforts were made to avoid the situation in which a health worker was observed or interviewed by a supervisor, co-worker, or peer[9]. For 8 days, surveyors practiced survey procedures in a classroom setting and then in health facilities that were not in the sample. Concordance testing was conducted to assess observation skills (with role-playing and videos of ill patient consultations) and re-examination skills (with patients in a hospital), and training continued until concordance (i.e., percent agreement between surveyors and a “gold standard” determined by the survey supervision team) was at least 90%; concordance was typically about 96%.

Dates of survey visits were not announced in advance to health facility staff. On the day of the survey visit, survey teams arrived at each facility before regular working hours began.

---

was in the southern half of the province. Each team was assigned one group. For each group, we determined a driving route that minimized distances between facilities, thus creating a “ring” of facilities. We randomly selected a starting point for each ring, and that facility was visited on day 1 of the survey. Dates of subsequent facility visits corresponded to the order of facilities in the ring (e.g., the next facility in the ring was visited on the survey day 2, etc.). For the southern ring, we inserted one “blank”, which corresponded to a date on which no facility would be visited (this blank ensured that all 17 week days during the survey period had a chance to be included).

[7] If <21 patients, the sampling fraction was 100% (include all initial consultations); if 22–42 patients, the sampling fraction was 50% (every other initial consultation); if 43–63 patients, the sampling fraction was 33%; if 64–84 patients, the sampling fraction was 25%, etc.

[8] Write numbers (e.g., 1, 2, 3, etc.) on slips of paper, put the papers in a bag, and choose one.

[9] After the survey, surveyors could only recall one or two instances of this occurring.

Teams carried a letter from the Ministry of Health specifying the purpose and nature of the assessment. Teams met with the facility director and all health workers who were to perform consultations with ill patients that day to introduce the team, describe the purpose of the visit (emphasizing that results will be used to assess and improve health services—not to punish health workers), and ask for verbal consent from health workers (see below). To avoid coercion, consent was obtained by a team member who was not the health worker's supervisor. Survey teams explained that after patients were re-examined, some patients might receive additional treatments in accordance with the national policy. After introducing themselves to health facility staff, the team asked for a brief tour of the facility and worked with the staff to establish a post for the interview, re-examination, and laboratory testing (the "IRLT" post). Observation, interviews, examinations, and laboratory testing began with the first enrolled patient.

Questionnaires were prepared in English and translated into Portuguese. Questions for patients were translated into Umbundu, the local language. Questionnaires were back-translated to verify accuracy. Interviews were conducted in the language that the subject was most comfortable speaking.

As ill patients arrived at the health facility, the driver introduced himself, described the purpose of the survey, and asked each patient if he or she had come for an initial consultation (Annex 1). The driver gave a card with a sequential identification number to each patient who had come for an initial consultation. At the end of the day, the team used the number of distributed patient identification cards to confirm the total number of initial consultations performed at the facility that day.

When patients coming for an initial consultation arrived at the facility (or were identified in the queue that might have formed before the survey team arrived), selected patients were met by a surveyor. Surveyors introduced themselves and requested verbal consent. If the patient agreed, the surveyor continued with the following steps. If the patient refused, the surveyor approached the next selected patient. If a surveyor noticed that a consented patient was going to be seen by a health worker who refused to participate in the survey, the surveyor was instructed to stop following the patient, end data collection for that patient, write "health worker refusal" on the data collection form (without writing the health worker's name), and return to the patient queue and meet the next eligible patient[10].

a) *Observation of the consultation*. The surveyor silently observed the consultation and used a standardized checklist to record the health worker's assessment, diagnoses, treatments, and counseling of the patient (see Annex 2 for the checklist). At the end of the consultation, the surveyor asked the health worker: 1) what the patient's diagnoses were, 2) what treatments were given to the patient during the encounter, and 3) what medications the health worker prescribed. If the surveyor observed that the health worker's actions were threatening the life of the patient (e.g., a health worker preparing to give an injection that appeared to be a lethal overdose, or a health worker not treating

[10] By the end of the survey, we determined that no health worker refused to participate.

obvious cardiopulmonary distress), then surveyors were trained to intervene and assist the health worker to give life-saving treatment or avoid life-threatening medical errors[11].

b) *Follow the patient*. After the consultation, surveyors followed patients through all other parts of the facility that the patient visited (e.g., laboratory, pharmacy) and silently observed all that occurred. All malaria-related treatment instructions and counseling messages were recorded.

c) *Exit interview and re-examination*. When patients were ready to leave the facility, surveyors took patients to the IRLT post, where surveyors interviewed patients and performed a focused re-examination using a standardized form (see Annexes 3, 4a, and 4b). The interview was used to determine if patients understood the information given by the health worker regarding diagnosis, referral, treatment, follow-up, and home care. All dispensed medications were recorded. Questions regarding satisfaction were also asked. The team's laboratory technician was stationed at the IRLT post, and after the interview and re-examination, surveyors introduced patients to the laboratory technician and left patients with the technician. Surveyors checked all survey forms for accuracy and then returned to the health facility queue to meet the next selected patient. After the re-examination (and laboratory testing, for some patients), if the survey team supervisor determined that a patient did not receive adequate (i.e., life-saving) treatment for malaria or any other potentially life-threatening illness, the supervisor provided treatment without charge to the patient.

d) *Laboratory testing*. We tested non-pregnant patients ≥5 years old with suspected malaria ("suspected malaria" defined according to national guidelines, see details below). Survey team laboratory technicians drew several drops of blood by finger stick with a single-use sterile lancet to make a blood smear and perform a RDT (Paracheck®, Orchid Diagnostics, Mumbai, India). The blood smear was air dried in the field; then, after the survey, it was stained with Giemsa stain and read by an expert microscopist to evaluate health facility diagnostics[12]. The RDT was read in the field after 15–25 minutes to ensure that parasitemic patients were treated with an antimalarial. Note that we did not test two groups of patients that the "old" (pre-September 2007) national guidelines recommended testing: under-5s[13] and pregnant women with suspected malaria[14]. The reasons were that we wanted to minimize unnecessary patient discomfort and the time patients spent with surveyors. Due to a misunderstanding, we thought that the policy stated that these two groups of patients should be treated with an antimalarial regardless of the test result. Therefore, because testing did not seem to effect the treatment, the survey protocol did not include testing for these two groups.

---

[11] By the end of the survey, we determined that no consultation was interrupted to prevent a life-threatening situation.

[12] The microscopists did not report any problems with the transportation of the slides.

[13] Recall that the "new" national guidelines (announced in September 2007) did not recommend testing under-5s.

[14] During the survey, five patients who would not have been tested according to the protocol were tested for clinical reasons (e.g., patients with an ambiguous clinical presentation whom the survey team tested to clarify the diagnosis).

e) *Health worker interview and health facility assessment*. Teams remained at health facilities until the regular closing time. After the facility closed, or in the late afternoon if no patients were being seen, surveyors used a standardized questionnaire to conduct interviews with facility staff (Annex 5) to obtain information on health worker training, supervision, and knowledge of malaria case management. To avoid making health workers feel uncomfortable, health worker interviews were conducted in private by a surveyor who was not a supervisor of the health worker. If a health worker left before the facility closing time, surveyors attempted to administer the interview before the worker left. Meanwhile, another surveyor conducted an inventory of the health facility's equipment and drug stocks with a checklist (Annex 6).

Definitions

Defining the standard or guideline for assessing the quality of laboratory testing, diagnosis, and treatment of malaria was complicated by several factors: 1) national policy documents were somewhat unclear (see Introduction); 2) training materials for health workers in Huambo lacked precision in some places (e.g., terms such as "fast breathing" were not defined [Figure 2]); 3) the survey protocol did not include malaria laboratory testing for all patients who should have been tested, according to the national guideline; and 4) national policy changed around the time the survey was conducted. We therefore developed two algorithms that were based on Angolan guidelines and included enough precision to analyze our sample of consultations. The first of these analysis algorithms (Figure 3) was used to assess case-management quality according to the old policy (pre-September 2007) as it was implemented in Huambo Province. The second analysis algorithm (Figure 4) was used to assess quality according to the new policy (announced in September 2007) that the NMCP intended for use in all parts of Angola with hyper- and meso-endemic malaria transmission, including Huambo. The only differences between the algorithms are that the new algorithm does not recommend malaria testing for under-5s with suspected malaria and that under-5s with suspected malaria and another cause of fever (e.g., pneumonia) should be treated for malaria, as well as for other causes of fever (as recommended in WHO's Integrated Management of Childhood Illness [IMCI] guidelines [Gove et al., 1997]).

Both algorithms were based on training materials used in Huambo and extensive discussions with in-country staff, and both algorithms closely resembled the guidelines on which they were based. Although the survey was conducted 1–2 months after the new policy was announced, the new policy did not practically affect implementation activities in Huambo during the survey (only three health workers had been trained in the new policy before survey teams visited the workers' health facilities). Thus, during the survey, virtually all health workers would have been expected to follow the old policy. Due to the timing of the survey, we were able to perform analyses that looked back (through the lens of the old policy, to evaluate how well the old policy had been implemented), and forward (through the lens of the new policy, to identify gaps and develop practical guidance on strengthening the implementation of the new policy).

The two analysis algorithms were used to make "gold standard" determinations of who had suspected malaria, who should have been tested, and who should have received antimalarial

therapy. Case-management quality was assessed by comparing the observed health worker's diagnosis and treatment with the gold standard diagnosis and treatment. The diagnosis and treatment portions of the algorithms were not based on the survey laboratory results; instead, the algorithms used information that should have been available to health workers (e.g., results of microscopy and RDTs ordered by health workers). This approach is best for evaluating adherence to a guideline, as it prevents classifying health worker practices as erroneous if laboratory results from the health facility did not match survey laboratory results. Survey laboratory results were used in a separate analysis to evaluate quality of care for "true" cases (i.e., patients with documented parasitemia), although this approach was greatly limited by the fact that only two patients were parasitemic (see Results). The analysis algorithms used clinical signs and symptoms from patient interviews and re-examinations performed by surveyors.

Note that the analysis algorithms had a limitation. The national policy required test results to determine which patients with suspected malaria should be treated, but in routine practice, health workers often did not test these patients. Therefore, the gold standard diagnoses and treatments (according to information that should have been available to health workers) were somewhat ambiguous for patients not tested according to the guidelines. A similar situation existed for patients without suspected malaria (who did not need testing) who were tested by health workers. To address these issues, investigators most closely involved in training based the analysis algorithms on the case-management principles conveyed to health workers during training (e.g., patients with a febrile illness and no identifiable cause should be treated with an antimalarial).

The definition of suspected malaria was either fever (history of fever or measured axillary temperature >37.5ºC) <u>or</u> at least three of the following non-fever symptoms: headache, joint pain, chills, sweating, anemia, cough (applies to children only), anorexia, fatigue, vomiting, or diarrhea. Uncomplicated malaria was defined as malaria (determined by the analysis algorithms) with no signs of severe illness (Box 1), and complicated malaria was defined as malaria with at least one sign of severe illness. Definitions of non-malaria causes of fever, which are used by the analysis algorithms, are shown in Box 2.

The definitions of treatment quality were based on surveyor-measured patient weights (with one exception[15]) and the NMCP's antimalarial dosing guidelines [Angola NMCP 2007]. However, as with the diagnostic guidelines in the NMCP's policy document, a lack of precision for AL dosing[16] necessitated the development of a slightly modified version for the analysis (Box 3). In practice, difficulty in applying the NMCP's dosing guidelines was rare (only two patients); and in these cases, AL dosing was considered correct. The quality of malaria treatment was categorized as either: 1) recommended (treatment prescribed by the health worker exactly matched the analysis algorithms, including drug type, dosage, and duration of treatment), 2) adequate (health worker treatments were not recommended, but still considered life-saving), or

---

[15] One adult patient with a missing weight was dosed in the ">35 kg" category.

[16] The problem was that weight categories in the NMCP guidelines (<5 kg, 5–14 kg, 15–24 kg, 24–34 kg, and >35 kg) had gaps and were not mutually exclusive. For example, it was not clear how to dose a patient weighing 14.5 kg (in between two weight categories) or 24.0 kg (in two categories).

3) inadequate (neither recommended nor adequate). These three categories correspond to correct treatment, minor errors, and major errors, respectively [Rowe et al., 2003].

Analysis

Data were double-entered with SPSS Data Entry version 1.0 (SPSS Inc., Chicago, Illinois) and validated against paper questionnaires. Analyses were conducted with SAS version 9.1 (SAS Institute, Inc., Cary, North Carolina). Weights (for patient-level indicators only) had two components that were multiplied together. The first weight component was 1 / the patient selection probability at health facility *f*, which equaled 1 / [(the probability of selecting facility *f*) x (the probability of selecting patient *p* given that one is sampling from facility *f*)], or 1 / [(33 selected facilities / 57 eligible facilities) x (the sampling fraction at facility *f*)]. The second weight component, which adjusted for non-response, equaled the number of eligible patients selected for enrollment (i.e., patients enrolled and included in the analysis + selected patients missed by surveyors + refusals + withdrawals + patients lost to follow-up) / (patients enrolled and included in the analysis). For example, in health facility 36 (Chiumbo Health Center), the sampling fraction was 50% (i.e., every other eligible patient was selected for enrollment); thus, the first weight component was 1 / [(33/57) x (0.50)], or 3.455. In Chiumbo, five eligible patients were selected for enrollment, but only four were actually enrolled and included in the analysis (one patient refused); thus, the second weight component was five patients selected / four actually enrolled, or 1.250. The final weight for all the patients in Chiumbo was the product of the two weight components (i.e., 3.455 x 1.250, or 4.318). In other words, in Chiumbo, each patient included in the analysis represented about four patients seen in the 57 eligible health facilities on one weekday during regular hours during the 4-week survey period. Overall, final weights ranged from 1.727 to 40.642.

We performed descriptive analyses of patient and health worker-level indicators with the SURVEYFREQ procedure, which uses the Taylor expansion method to account for cluster sampling and unequal analysis weights. Analyses of health worker indicators were not weighted, as selection probabilities were equal. Analyses of patient-level indicators were weighted, unless otherwise noted. As the sample size of health facilities (n = 33) was a relatively large proportion of all facilities in the sampling frame (N = 57), confidence intervals (CIs) for health facility-level indicators were adjusted with the finite population correction factor—i.e., for a proportion, *p*, the standard error = {square-root [$p(1 - p)$/n]} x {square-root [(N – n)/(N – 1)]}. If $p = 0\%$ or 100% (and thus the estimated standard error would be zero), CIs were estimated using the exact binomial method.

For descriptive analyses that required a clustered analysis, we also estimated the design effect and intraclass correlation coefficient (ρ or "rho"). The design effect is "the ratio of the actual variance of a sample to the variance of a simple random sample of the same number of elements" [Kish, 1965, page 258], and it equals $\rho(m - 1) + 1$, where *m* is the mean cluster size (i.e., average number of consultations in the analysis per health facility) [Kish, 1965, page 162]. The intraclass correlation coefficient is a measure of the homogeneity of elements within clusters and has a maximum value of +1 when there is complete homogeneity within clusters, and a minimum value of $-1/(m - 1)$ when there is extreme heterogeneity within clusters [Kish, 1965, page 171]. The output of the SAS SURVEYFREQ procedure includes an estimate of the design

effect. We then estimated ρ with the expression: ρ = (design effect – 1)/(m – 1). Both the design effect and ρ quantify the similarity of outcomes (i.e., indicator values of case-management quality) for consultations performed in the same health facility. For example, large design effect values (e.g., 10) and ρ values close to one mean that case-management quality for the patients in any given facility was similar (e.g., most patients treated correctly or most treated incorrectly). The distribution of these measures were examined because they are useful for designing future cluster surveys, and very little published information exists on design effects and intraclass correlation coefficients from health facility surveys in developing countries [Rowe et al., 2002].

Statistical modeling of dichotomous patient-level outcomes was performed with logistic regression using the SURVEYLOGISTIC procedure, which also uses the Taylor expansion method to account for cluster sampling and unequal analysis weights. All models used analysis weights. Variables with a p-value <0.15 in the univariate analysis were included in a multivariate model (the full model). As we were concerned about over-specifying models[17] in analyses with small sample sizes, we tested reduced models by removing variables with a multivariate p-value >0.10. However, variables with a multivariate p-value >0.10 were not removed if doing so changed the odds ratio (OR) of another variable by >20%. To assess confounding by excluded variables (i.e., those with a univariate p-value ≥0.15), we added excluded variables to the reduced model one at a time and retained them if ORs of other variables changed by >20%. Hypothesis testing and CI estimation were done with an alpha level of 0.05. Results with a p-value from 0.05 to 0.10 were considered to have borderline statistical significance.

RESULTS

Enrollment

Survey teams visited all 33 sampled health facilities, and all visits occurred on schedule. In these health facilities, 100 health workers performed outpatient consultations. Ninety-three (93.0%) of these workers were interviewed, and seven left before interviews could be administered. No health worker refused to participate in the survey. Not all health workers performed a consultation on a patient included in the analysis. Of the 93 health workers interviewed, 64 treated at least one included patient; and of the 7 health workers missed, 6 treated at least one included patient. That is, patient-level indicators of case-management quality (see below) reflected the practices of 70 health workers (64 interviewed, 6 missed).

During the 33 health facility visits, 778 patients came for an initial consultation and thus were eligible. Teams used systematic sampling to select 389 of these patients for inclusion. Of these 389 patients: 177 were included in database (i.e., response rate = 177/389, or 45.5%), 170 were missed (i.e., by the time of the consultation, no surveyor was available to begin the "follow the patient" procedure), 38 refused, 3 withdrew after enrollment, and 1 was lost to follow-up (i.e., the patient left the facility before completing all survey steps). Response rates in individual health facilities varied from 16.7% to 100% (median = 44.4%).

[17] That is, having too many variables in a model, relative to the sample size.

Although many sampled patients were not surveyed (i.e., 170 missed + 38 refusals + 3 withdrawals + 1 lost, or 212 patients), most (170/212, or 80.2%) were missed patients. These missed patients were unlikely to have been much different from included patients, as they were essentially missing at random. Missed patients might have been somewhat more likely than included patients to have been seen in mid-morning, as surveyors were more likely to include patients in the early morning (at the beginning of the queue) and late morning (after the earliest consultations had been completed and surveyors could return to the queue). If missed patients are not considered, the "participation rate" (i.e., the proportion of patients approached for consent who were included) was 80.8% (177 included / [177 + 38 refusals + 3 withdrawals + 1 lost]).

Health facility characteristics

Of the 57 eligible health facilities, 8 (14.0%) were hospitals, 23 (40.4%) were health centers with adequate infrastructure, 25 (43.9%) were health centers with low infrastructure, and 1 (1.8%) was a health post (Table 1). This distribution was similar to that of the 33 facilities in the sample. The distributions of municipalities where health facilities were located were also similar between the sample and all eligible health facilities. All facilities in the sample were operated by the government. Twenty-three facilities were in the initial five municipalities, and 10 were in the other six municipalities.

Regarding caseload, 1103 patients came to the 33 sampled facilities on the day of the survey visit: 778 (70.5%) for initial consultations (and thus were eligible for inclusion) and 325 (29.5%) for follow-up visits. The median number of total consultations per facility per day was 26 (range: 12–119), the median number of initial consultations per facility per day was 18 (range: 5–77), and the median number of follow-up visits per facility per day was 8 (range: 3–42). By dividing these figures by the sampling fraction of health facilities (0.579, or 33 facilities sampled / 57 total eligible facilities), one can estimate that in Huambo Province, during the survey period, 1905 patients (1344 initial consultations and 561 follow-up visits) came each weekday during regular working hours to all health facilities in which ACT implementation had occurred. If one assumes that weekend caseloads are similar to weekday caseloads, no after-hours consultations (a plausible assumption for many health centers), and a population of 2.3 million in Huambo Province, then a rough estimate of the utilization of public health facilities in which ACT implementation had occurred was about 0.83 consultations per 1000 population per day (i.e., [1905 consultations / 2.3 million / day] x 1000) during the early rainy season, or about 25 consultations per 1000 population per month (i.e., 0.83 x 30 days/month).

Our results revealed a very large number of health workers (3564 workers) in the 57 eligible facilities (Table 2, last row). This estimate represents all health workers assigned to the facilities, including all departments (inpatient, outpatient, laboratory, administrative, etc.) and all shifts (i.e., not all these health workers work at the same time). The large number of health workers was driven by nurses, which were nearly three-quarters of the total (Table 2, row 2). Not only was the median number of nurses per facility high (24 nurses/facility), but many were concentrated in just a few facilities. Among the 33 surveyed facilities, respondents at six (18.2%) reported that each had between 92 and 347 nurses per facility (a total of 942 nurses in these six facilities). That is, nearly 60% (942/2577) of all nurses in the surveyed facilities worked in one-

fifth (6/33) of facilities. Of these six highly staffed health facilities, three were municipal (district) hospitals and three were health centers.

According to health facility respondents, over a thousand health workers had received formal in-service training related to malaria case management. The proportions of health workers who received the training were generally modest; however, as described above, the total number of health workers was quite large. Also, health workers who did not receive formal training on malaria case management might have received informal training from their peers (trainers encouraged participants to train health workers in their facilities). Notably, most of the training focused on nurses, and a median of five nurses per facility had received training on ACTs. Coverage of training, in terms of having at least one health worker trained per facility, was generally excellent (Table 3). All facilities had at least one nurse trained to use ACTs and RDTs, and nearly all (29/33, or 87.9%) facilities had at least one nurse or midwife trained in IPTp. The exception was IMCI training: only 39.3% (13/33) health facilities had at least one IMCI-trained health worker.

Regarding the equipment needed to manage malaria cases, nearly all health facilities had a thermometer and a scale for weighing children; but only about half of facilities had a booklet or chart with ACT algorithms (Table 4). In the initial five municipalities, 47.8% (i.e., 11/23) had a booklet or chart with ACT algorithms versus 50.0% (i.e., 5/10) in the other six municipalities. All facilities were able to perform malaria testing: 13 (39.4%; 95% CI: 28.5–50.3%) had both microscopy and RDTs available, 6 (18.2%; 95% CI: 9.6–26.8%) had microscopy only, and 14 (42.4%; 95% CI: 31.4–53.5%) had RDTs only.

AL was the only ACT used in Huambo. On the day of the survey visit, all health facilities had AL in stock, and most had all four AL blister packs in stock (Table 5, columns 1 and 2). Notably, only three-quarters of facilities had stocks of the AL blister pack for young children (i.e., 5–14 kg). In contrast, about two-thirds of facilities had stock-outs of AL in the past three months (Table 5, column 3). Results were similar for the initial five municipalities and the other six municipalities[18]. No facility had parenteral artemisinins in stock. Only one-third of facilities had oral quinine in stock, and one-half of facilities had injectable quinine or quinidine (for complicated malaria) in stock. Most facilities had other essential drugs in stock (oral antibiotics and iron), but only about two-thirds had oral rehydration solution. Many facilities had had stock-outs of these essential drugs in the past three months.

In summary, health facilities were generally well prepared to manage malaria cases. All facilities had staff trained in the ACT policy, malaria diagnostic testing available, and AL in stock. The main weaknesses were that one-quarter of facilities did not stock AL blister packs for young children, only about half of facilities had parenteral drugs for severe malaria cases, and about two-thirds had stock-outs of AL in the past three months.

[18] The proportions of facilities that had AL in-stock every day in the past 3 months for each of the four AL blister packs were: 21.7%, 34.8%, 34.8%, and 21.7% (for each of the four AL blister packs, respectively) for the initial five municipalities; and 20.0%, 40.0%, 40.0%, and 50.0% for the other six municipalities.

Health worker characteristics

We interviewed 93 health workers. The median age was 36 years (range: 21–70; 25–75% interquartile range [IQR]: 29–47), and about half (48/93, or 51.6%) were female. Nearly all (91/93, or 97.8%) were nurses, and the remaining two (2.2%) were physicians. Of the 91 nurses, 41 (45.1%) were an *Enfermeira Básica* (two years of pre-service medical training at a high school level), 39 (42.9%) were an *Enfermeira Média* (four years of pre-service medical training at a high school level), and 11 (12.1%) were an *Enfermeira Geral* (four years of pre-service medical training at a high school level plus one year of training at the college level). The median number of years of professional training was four (range: 1–25; IQR: 2–4).

Three-quarters (70/93, or 75.3%) of health workers reported receiving in-service training unrelated to malaria. About one-third had been trained in diarrhea case management and nutrition, and about one-quarter had been trained on acute respiratory infections and immunizations (Table 6). More than half of health workers had received training on some other topic, such as essential medicines or "*Doentes Correntes*" (i.e., common diseases, such as diarrhea, respiratory infections, and malaria). Most of these training courses had occurred in the previous 3–5 years, and course duration was typically 3–5 days.

Sixty percent (56/93) of health workers reported participating in at least one formal training course on AL[19], and about one-fifth (20/93, or 21.5%) were trained more than once (Table 7). Nearly two-thirds (64.5%) of health workers reported receiving informal training on ACTs, and 77.4% had either formal or informal training[20]. If one considers each formal training course or informal session as an "exposure" to AL training, then the numbers of workers with 0, 1, 2, 3, 4, and 5 training exposures were 21, 26, 27, 12, 6, and 1, respectively. The median number of days of AL training for all health workers was 3 (range: 0–45.5; IQR: 0.5–4.5).

Among the 56 health workers with at least one formal training course on AL, a large majority (50/56, or 89.3%) reported that the course included training on RDT use (Table 8). Training duration was usually 3 days (range: 1–15). Most (75.0%) courses occurred in 2007. From training records, we determined that only three health workers in the survey had received training in the new policy (announced in September 2007), and these three health workers only performed four of the consultations in the analysis. It should be mentioned that while this number reflects training in the new policy during the survey, it is now (at the time of this writing) an underestimate because training in the new policy has continued since the survey ended.

The number of times health workers were supervised (any type of supervision) in the 6 months before the survey ranged from 0 to 5 (median = 1), with nearly one-quarter (26.9%) of workers reporting no supervision (Table 9). Supervision visits in which the supervisor observed

[19] Results were similar for the initial five municipalities (40/66, or 60.6%) and the other six municipalities (16/27, or 59.3%).

[20] Results were similar for the initial five municipalities (51/66, or 77.3%) and the other six municipalities (21/27, or 77.8%).

and provided feedback on a consultation numbered from 0 to 5 (median = 0), with nearly two-thirds (65.6%) of workers reporting no supervision with observation and feedback. From the perspective of PMI-supported activities, this result was not surprising because the ACT-related supervision during the first year of scale-up focused primarily on pharmaceutical management. Supervision visits on AL use numbered from 0 to 3 (median = 1), with half (49.5%) of workers reporting no supervision on AL. Altogether, 50.5% (47/93) of health workers were supervised at least once on AL use in the past six months; this proportion was higher in the initial five municipalities (38/66, or 57.6%) than in the other six municipalities (9/27, or 33.3%). Of the 47 health workers supervised at least once on AL use, almost half (20/47, or 42.6%) reported never having received supervision with observation and feedback on a consultation, which confirms that supervision usually did not focus on the clinical use of AL.

Regarding job aids and caseload, about three-quarters of health workers (69/93, or 74.1% [95% CI: 63.1–85.3]) reported that they had an ACT job aid (e.g., clinical algorithm or dosage guide) in the consultation room[21]. The total number of patients seen per health worker on the day of the survey visit ranged from 1–44 (median = 13; IQR: 8–21) (Table 10). One in six health workers (15/92 [1 missing], or 16.3%) had high caseloads of $\geq$25 patients during the day.

Our knowledge assessment revealed that no health worker could repeat the complete description (or case definition) of which patients should be tested for malaria, for either the old or new policy (Table 11). Each case definition contained 14 elements (Annex 9), and health workers needed to mention all 14 elements to have demonstrated knowledge of the complete case definition (a verbatim repetition of the case definition was not required). After examining the specific elements of each worker's response, we found that they contained a median of only two (14.3%) of the criteria of the case definition, for both the old and new policies. Nearly two-thirds (59/90, or 65.6%) of health worker responses included fever as a criteria for testing[22]. Three (3.3%) health workers stated that under-5s should not be tested (i.e., the new policy), although these workers were not the three who had been trained in the new policy. About one-third of workers (33/90, or 36.7%) mentioned signs or symptoms not in the policy (i.e., extra criteria, such as a child who cries a lot or stomach ache).

Health worker responses to a series of case scenarios revealed several trends (Table 12). First, in scenarios 1–3 with a febrile patient and a negative malaria test, most health workers (72.0–81.7%) seemed to ignore the test result and gave a diagnosis of malaria or suspected malaria[23]. In these three scenarios, for virtually all (96–100%) patients diagnosed with malaria or suspected malaria, workers said they would treat with an antimalarial. Second, most workers (83.9–89.2%) correctly responded that these patients did not need hospitalization. Third, in scenario 4, most workers (63/93, or 67.7%) correctly diagnosed the severe malaria, most

---

[21] This proportion (74.1%) is higher than the proportion shown in Table 4 (48.5%, in row 4) because an "ACT job aid" had a broader definition (it could include a simple ACT dosage guide, as well as an ACT policy document or clinical algorithm). The result in Table 4 was only for ACT treatment algorithms.

[22] Results were similar for the initial five municipalities (43/64, or 67.2%) and the other six municipalities (16/26, or 61.5%).

[23] Results were similar for the initial five municipalities and the other six municipalities; e.g., for scenario 2, the proportions were 71.2% (47/66) and 74.1% (20/27), respectively.

correctly diagnosed cases (53/63, or 84.1%) were treated with an injectable antimalarial, and nearly all cases (87/93, or 93.5%) were referred for hospitalization. Fourth, in scenarios 5–7, most workers (76.3–87.1%) correctly responded that they would test the patient (in accordance with the old policy). Finally, regarding a knowledge assessment score that summarized responses across all seven scenarios, the median percentage of questions correctly answered per health worker was 56.3% (range: 31.3–87.5%; IQR: 50.0–62.5%).

Patient characteristics — demographics, consultation attributes, and illnesses

Weighted analyses of the 177 included initial consultations showed that patient ages ranged from 0–80 years (median = 8, IQR: 1–28); 45.0% were under-5s (Table 13). About half (55.9%) of patients were female, and 2.2% of the 177 patients (n=5; 95% CI: 0–4.8%) reported being pregnant. All patients were seen in health facilities with AL in stock (Table 14), although only 70.8% of consultations occurred in facilities with stocks of the AL blister pack for young children (i.e., patients weighing 5–14 kg). All patients were seen in facilities able to perform malaria laboratory testing. About half (53.9%) of patients were seen by a health worker with formal training on AL use, and 75.3% of patients were seen by a worker with any AL training (formal or informal training). Only 30.8% of patients sought care on the day of illness onset or the next day.

The chief complaint of about half of patients was fever or malaria (Tables 15 and 16). Not surprisingly, this complaint was more common among under-5s. The distribution of chief complaints was similar for data collected during consultations and re-examinations, and the agreement between these two data sources was excellent (>90%) (Table 17).

Febrile illness was defined as a history of fever or a measured axillary temperature >37.5ºC, according to the surveyor's re-examination. Among all 177 patients, the prevalence of febrile illness was 70.5% (n=119; 95% CI: 62.0–78.9). Among the 72 under-5s, the prevalence was 74.1% (n=58; 95% CI: 60.2–88.0); and among the 105 patients ≥5 years old, the prevalence was 67.5% (n=61; 95% CI: 55.0–80.0). Notably, among the 119 patients who had a febrile illness, only 69.5% (95% CI: 59.0–80.1) gave a chief complaint of fever or malaria. This percentage was 85.8% for under-5s and 54.9% for patients ≥5 years old. These results show why it is important for health workers to ask about fever and measure temperatures for all patients. Relying only on chief complaints could cause health workers to miss many cases of febrile illness.

The definition of suspected malaria in Huambo was: either fever (history of fever or measured axillary temperature >37.5ºC) <u>or</u> at least three of the following non-fever symptoms: headache, joint pain, chills, sweating, anemia, cough (applies to children only), anorexia, fatigue, vomiting, or diarrhea. By this definition, 77.8% of all patients had suspected malaria; this proportion was similar for under-5s and older patients (Table 18). Among patients with suspected malaria, about half (48.0%) had clinical signs of a non-malaria cause of the febrile illness—most commonly an acute respiratory infection. The presence of a non-malaria cause of fever did not exclude the possibility of malaria; but it does illustrate the need for malaria testing and a comprehensive case-management approach, such as IMCI for under-5s and Integrated

Management of Adolescent and Adult Illness for older patients [WHO 2004], to help health workers identify and treat non-malaria illnesses.

Among 72 under-5s, 62 had suspected malaria. Of these 62 patients, 57 (91.9% [unweighted], or 57/62) would have been detected by history of fever alone, and 58 (93.5% [unweighted]) would have been detected by history of fever or measured temperature >37.5ºC. If suspected malaria had been defined as "fever history or measured temperature >37.5ºC", the inclusion of "or at least three non-fever symptoms" added only four more patients (i.e., an additional 6.5% [unweighted], or 4/62). Thus, the added benefit of ≥3 non-fever symptoms was small.

Among 105 patients ≥5 years old, 74 had suspected malaria. Of these 74 patients, 59 (79.7% [unweighted], or 59/74) would have been detected by history of fever alone, and 61 (82.4% [unweighted]) would have been detected by history of fever or measured temperature >37.5ºC. The inclusion of ≥3 non-fever symptoms added 13 patients (i.e., an additional 17.6% [unweighted], or 13/74). Thus, the added benefit of ≥3 non-fever symptoms was moderate.

According to the old policy gold standard analysis algorithm, among all patients, 0.8% (1 patient) had complicated malaria, 35.0% (58 patients) had uncomplicated malaria, and the remaining 64.2% (118 patients) did not have malaria (Table 19). These proportions were similar for patients under-5 and ≥5 years old.

According to the new policy gold standard analysis algorithm, 4.3% (4 patients) had complicated malaria, 45.9% (74 patients) had uncomplicated malaria, and the remaining 49.7% (99 patients) did not have malaria (Table 20). The prevalence of complicated and uncomplicated malaria were greater for under-5s (7.9% and 54.8%, respectively) than for patients ≥5 years old (1.4% and 38.7%, respectively). The difference existed because the new policy (for treatment purposes) considered under-5s with suspected malaria as cases (unless for some reason, the child was tested and the result was negative).

Patient characteristics — quality of assessment and use of diagnostic testing

We evaluated the quality of health worker assessments of patients' signs and symptoms by estimating the proportion of patients for whom health workers had determined whether or not patients had a given sign or symptom, where "determined" meant whether the health worker had been exposed to the information by any means (e.g., information spontaneously offered by the patient, provided by the patient in response to a health worker's question, or obviously evident). This approach avoids penalizing health workers who do not ask for a clinical sign when the patient offers the information spontaneously or when the sign is evident (e.g., a seizure or obvious coughing).

We found that the determination of fever history was generally very good: the symptom was determined for 87.6% of patients (Table 21, row 1). Even when patients did not spontaneously offer a history of fever, health workers asked for the information in a large majority of cases. Among all 177 patients, a history of fever was spontaneously offered in 38.2% of consultations (53.2% for under-5s and 25.8% for patients ≥5 years old); and when the

information was not spontaneously offered, health workers asked in 80.0% of consultations (88.8% for under-5s and 75.5% for patients >5 years old). Health workers measured the patient's temperature in only 25.9% of consultations (Table 21); this proportion was somewhat higher in the initial five municipalities (28.6%) than in the other six municipalities (16.6%).

Assessment quality seemed poor for all other symptoms needed for the case definition of suspected malaria (Table 21). However, we were concerned that health workers might not have been asking about these symptoms because patients often met the case definition of suspected malaria simply by having fever. Therefore, we repeated the analysis for the subgroup of patients without fever (i.e., the patients for whom it was necessary to ask about the non-fever symptoms). This analysis revealed somewhat better quality, but still the determination for the various symptoms ranged from only 0 to 44.6% of patients (Table 22, analysis of all patient ages).

In the analysis of the use of malaria laboratory testing, microscopy and RDTs were considered equally appropriate. Among all 177 patients, 77.8% (n=136; 95% CI: 69.5–86.2) needed testing according to the old policy, and 42.1% (n=74; 95% CI: 27.9–56.3) needed testing according to the new policy (i.e., same as the old policy, except under-5s with suspected malaria do not need testing). Altogether 28.5% (n=64; 95% CI: 17.9–39.2) of patients were tested, whether or not it was indicated. According to the old policy, only 30.7% of patients needing testing were tested (i.e., substantial under-use of testing)[24], 79.2% of patients not needing testing were not tested (i.e., little over-use of testing), and overall adherence to the policy was 41.5% (Table 23, top half of table). Results did not vary by age. Results for the new policy were similar to those from the old policy analysis.

Univariate statistical modeling identified several factors that were positively associated with malaria testing among patients needing testing according to the old policy: increasing supervision on AL use, lower health worker caseload (<25 versus >25 patients on day of survey visit), higher patient temperatures, and health facility type (health centers with low infrastructure versus hospitals) (Table 24). Although the continuous caseload variable was also associated with testing, the dichotomous variable (<25 versus >25 patients) had a better fit and was used in the multivariable model. Several factors were not associated with testing, but p-values were low enough ($p < 0.15$) to retain in the multivariable analysis: any AL training (formal or informal) and health worker age. The following factors were not associated with testing and not retained in the multivariable analysis: formal AL training, days of AL training, health worker knowledge score, health worker's sex, chief complaint of fever or malaria, patient's sex, and patient's age.

Multivariable modeling identified two factors with statistically significant associations with malaria testing (Table 25). First, the odds of testing (for patients needing testing according to the old policy) among patients seen by health workers with daily caseloads <25 patients were about 18-fold greater than the odds of testing by workers with daily caseloads >25 patients. Based on unadjusted results (Table 24), the proportion of patients tested by health workers with lower and higher caseloads were 49.0% and 7.5%, respectively—a large difference of 41.5 percentage points (%-points). Second, the odds of testing increased by about 2.5-fold for each

[24] Results were similar for the initial five municipalities (29.8%) and the other six municipalities (34.4%).

increase in patient temperature by 1ºC. Based predicted probabilities from the reduced model[25], for each 1ºC increase in temperature (in the 37–39ºC range), the proportion of patients tested increases by about 13–22 %-points. The multivariable model also revealed that the association between testing and training was of borderline statistical significance (p-values from the full and reduced models were 0.086 and 0.072, respectively). The odds of testing among patients seen by health workers with any AL training (formal or informal) was about 5-fold greater than the odds of testing among patients seen by workers without AL training. Based on unadjusted results (Table 24), the proportion of patients tested by health workers with any AL training and no AL training were 38.1% and 17.2%, respectively—a moderate increase of 20.9 %-points. The other variables in the model were not associated with testing. For example, the significant association between testing and supervision on AL in a univariate model disappeared in the multivariate model (i.e., the univariate association was confounded by other factors in the model).

Patient characteristics — results of malaria testing and quality of diagnosis

Seventy-four patients were tested by surveyors. As per the protocol, the 69 non-pregnant patients ≥5 years old with suspected malaria were tested with both RDTs and microscopy. Five other patients were tested for clinical reasons: two were tested with RDTs only (both were pregnant patients with suspected malaria) and three were tested with microscopy only (two under-5s with suspected malaria and one older patient). Thus, surveyors tested 72 patients with microscopy. Among the 69 patients tested by surveyors with both RDTs and microscopy, the sensitivity of RDTs performed by survey teams was 2/2, and the specificity was 59/67 (88.1%, unweighted). Among the 69 non-pregnant patients ≥5 years old with suspected malaria (all tested with microscopy), we found 3.4% (n=2; 95% CI: 0–8.5) were parasitemic with *P. falciparum.*

Health workers tested 64 patients (Table 26), and 62 of these patients had results available the same day: 50 had RDTs only, nine had microscopy only, and three had RDT and microscopy. Among the 27 patients tested by surveyors with microscopy (our gold standard for evaluating malaria diagnostics) and by health workers (with RDT or microscopy), the sensitivity of health worker testing was 2/2, and the specificity was 19/25 (76.0%, unweighted). For the three patients that health workers tested with both an RDT and microscopy, the results always agreed. Among the 24 patients that health workers tested with RDTs and who were tested by surveyors with microscopy, the sensitivity of health worker RDTs was 2/2, and the specificity was 17/22 (77.3%, unweighted). Among the five patients that health workers tested with microscopy and who were tested by surveyors with microscopy, the sensitivity of health worker microscopy was 1/1, and the specificity was 3/4.

According to the old policy, among all 177 patients, 66.1% (110 patients) of malaria-related diagnoses were correct[26], 20.1% (42 patients) were minor errors[27], and 13.9% (25

[25] Assuming the case load and training variables (each coded as 0 or 1) have a value of 0.5.

[26] Patients with malaria were diagnosed with malaria (including illness severity), and patients without malaria were not given a diagnosis of malaria.

[27] Incorrectly over-diagnosing uncomplicated malaria as complicated malaria, or over-diagnosing no malaria as uncomplicated malaria.

patients) were major (potentially life-threatening) errors[28] (Table 27). Diagnosis was somewhat better in the initial five municipalities (69.4% correct, 17.8% minor errors, and 12.7% major errors) than in the other six municipalities (54.5% correct, 27.7% minor errors, and 17.8% major errors). According to the new policy, the quality of diagnosis was somewhat lower: 61.8% (105 patients) of malaria-related diagnoses were correct, 15.0% (35 patients) were minor errors, and 23.2% (37 patients) were major errors (Table 28). The lower quality of diagnosis under the new policy can be explained by the fact that the 19 under-5s with suspected malaria, no other cause of fever, and no test (i.e., the 19 "extra" malaria cases under the new policy, relative to the old policy) were usually not diagnosed with malaria by health workers (only 7 of 19 diagnosed with malaria).

Although we focused on health workers' adherence to guidelines, in which the gold standard was based on our analysis algorithms (Figures 3 and 4), it is also useful to evaluate healthcare quality by comparing health worker diagnoses to: a) survey laboratory results (i.e., a standard based on true parasitemia), and b) health worker laboratory results (i.e., to evaluate how well workers interpreted or trusted their own results). As the number of patients with laboratory results was small, these results must be interpreted cautiously.

A comparison of health worker diagnoses with survey microscopy results showed that: a) for the one microscopy-positive case of uncomplicated malaria, the health worker's diagnosis was uncomplicated malaria (correct); b) for the one microscopy-positive case of complicated malaria, the health worker's diagnosis was uncomplicated malaria (major error); and c) for the 70 microscopy-negative cases, health workers correctly diagnosed no malaria in 34 cases (48.6%, unweighted), and incorrectly over-diagnosed malaria in 36 cases (51.4%, unweighted).

A comparison of health worker diagnoses with health worker laboratory results showed that: a) among 17 patients with a positive test (microscopy or RDT), health workers always correctly diagnosed malaria; and b) of 45 patients with a negative test (microscopy or RDT), health workers correctly diagnosed no malaria in 19 patients (42.2%, unweighted) and incorrectly diagnosed malaria in 26 patients (57.8%, unweighted). This pattern was similar for microscopy and RDT results. Thus, it appears that health workers always trusted positive tests, but only trusted negative results less than half the time.

We also compared our gold standard diagnoses (old and new policy, based on analysis algorithms) with survey microscopy results and found that: when microscopy was positive (n = 2), gold standard diagnoses were always some form of malaria (correct); and when microscopy was negative, gold standard diagnoses frequently over-diagnosed malaria. Neither result was surprising, as health worker testing detected the two microscopy-positive patients, and the analysis algorithms relied heavily on clinical findings.

When comparing the frequency of malaria cases (uncomplicated and complicated combined for simplicity) according to the various definitions described above, we found the following. Among all 177 patients, health workers diagnosed 75 patients with malaria; by the old policy gold standard, 59 patients had malaria; and by the new policy gold standard, 78 patients

[28] Incorrectly under-diagnosing complicated malaria as uncomplicated malaria, or under-diagnosing uncomplicated malaria as no malaria.

had malaria. Among the 69 non-pregnant patients ≥5 years old with suspected malaria who were tested with microscopy, two were parasitemic with *P. falciparum*.

Informal laboratory assessment

Although it was not a primary objective of the survey, we informally assessed health worker and laboratory worker practices and knowledge related to performing RDTs. Twenty-two staff were observed performing an RDT in a convenience sample of 20 health facilities. All the correct steps (i.e., 1 drop of blood and 6 drops of buffer solution) were followed in only half of observations (11/22, or 50.0%). The most common error was using an incorrect amount of buffer solution: staff were observed using 2 drops (in 2 of 22 observations, or 9.1%), 4 drops (2/22, or 9.1%), 5 drops (4/22, or 18.2%), 6 drops (correct amount; 13/22, or 59.1%), and 8 drops (1/22, or 4.5%). The amount of blood was usually correct: staff were observed using 1 drop (correct amount; in 19 of 22 observations, or 86.4%) and 2 drops (3/22, or 13.6%). After 21 of the observations, staff were asked how long they needed to wait before reading the RDTs. Most responses reflected the understanding that one should wait 15 minutes or a little longer: 15 minutes (from 10 of 21 staff questioned, or 47.6%), 10–15 minutes (4/21, or 19.0%), 15–25 minutes (2/21, or 9.5%), 15–30 minutes (1/21, or 4.5%), 15–20 minutes (1/21, or 4.5%), 10 minutes (1/21, or 4.5%), 5 minutes (1/21, or 4.5%), and "do not know" (1/21, or 4.5%). Still, one-third (7/21, 33.3%) of laboratory workers might have thought that waiting times were <15 minutes or were unsure. We also noticed that in some health facilities, test results were reported in batches instead of reporting each result as soon as it was ready. This practice caused unnecessarily long waiting times (an hour or more) for patients in some facilities (an RDT result should be available in <30 minutes).

Patient characteristics — quality of treatment and counseling

According to the old policy, among all 177 patients, 61.4% (105 patients) of prescribed malaria-related treatments were correct[29], 22.3% (42 patients) were minor errors[30], and 16.3% (30 patients) were major (potentially life-threatening) errors[31] (Table 29). Treatment was somewhat better in the initial five municipalities (64.7% correct, 20.7% minor errors, and 14.6% major errors) than in the other six municipalities (49.8% correct, 27.7% minor errors, and 22.5% major errors). The most common errors were prescribing no antimalarials for patients with uncomplicated malaria and prescribing AL for patients without malaria. Errors such as treating children <5 kg with AL, under-dosing AL, and treating with ineffective or non-recommended antimalarials were uncommon. Of the 5 pregnant patients, one had uncomplicated malaria; she was not treated with an antimalarial (a major error). The one patient with complicated malaria was treated with under-dosed AL (a major error).

[29] Patients with malaria were treated with the recommended antimalarial (including dosage and treatment duration), and patients without malaria were not treated with antimalarials.

[30] For patients with malaria: treatment with non-recommended (but still effective) antimalarials or non-toxic over-dosing a recommended antimalarial. For patients without malaria: treatment with any antimalarial.

[31] Treating malaria with an ineffective or under-dosed antimalarial, or no antimalarial. There is no major error category for patients without malaria.

Among the 59 patients with malaria according to the old policy gold standard, the quality of prescribed treatments was lower than that for all patients: only 49.0% (27 patients) of prescribed malaria treatments were correct[32], 5.4% (2 patients) were minor errors, and 45.6% (30 patients) were major errors. In an analysis of treatment quality from the patient's perspective (i.e., the patient or patient's caretaker left the facility with the antimalarial drug and demonstrated knowledge on how to administer it), only 27.1% of patients received recommended care, 5.4% received adequate (but not recommended) care, and 67.5% received inadequate care (Table 29).[33]

According to the new policy, treatment quality was somewhat lower than for the old policy: 56.0% (99 patients) of malaria-related treatments were correct, 17.2% (35 patients) were minor errors, and 26.5% (43 patients) were major errors (Table 30). Among the 78 patients with malaria according to the new policy gold standard, treatment quality was also lower than for the old policy: only 42.7% (33 patients) of malaria treatments were correct, 3.8% (2 patients) were minor errors, and 53.4% (43 patients) were major errors. The types of errors were similar to those found in the analysis of the old policy. Of note, there were major errors in the treatment of all four patients with complicated malaria.

Similar to our analysis of the quality of diagnosis, we compared health worker treatments to survey microscopy results and found that: a) for the one microscopy-positive case of uncomplicated malaria, the treatment was recommended (AL correctly dosed); b) for the one microscopy-positive case of complicated malaria, the treatment was inadequate (AL under-dosed); and c) for the 70 microscopy-negative patients, 34 (48.6% unweighted) correctly received no antimalarial, 32 (45.7% unweighted) incorrectly received AL, one (1.4% unweighted) incorrectly received quinine, and 3 (4.3% unweighted) incorrectly received an ineffective antimalarial.

A comparison of health worker treatments with health worker laboratory results showed that a large majority (84.7%) of the 17 patients with a positive test (microscopy or RDT) were correctly treated (Table 31). Among the 45 patients with a negative test, 43.8% correctly received no antimalarial, 56.2% incorrectly received an antimalarial (44.7% got AL, 9.1% got an ineffective antimalarial, and 2.4% got quinine). These results reflect the pattern seen in the comparison of health worker diagnoses with health worker laboratory test results (i.e., all test-positive patients and just over half of test-negative patients were diagnosed with malaria).

Health worker treatments generally agreed with their diagnoses. Among 73 patients whom health workers diagnosed with uncomplicated malaria, a large majority (83.6%) received AL; and among 102 patients without a diagnosis of malaria, nearly all (97.3%) received no antimalarial (Table 32). Both of the patients with a health worker diagnosis of complicated

[32] Treatment might have been somewhat better in the initial five municipalities (51.1% correct) than in the other six municipalities (42.7% correct), however these percentages are based on small numbers and have low precision.

[33] Results for the initial five municipalities were similar to those for the other six municipalities, although numbers were small.

malaria were treated with AL (one of these patients was seen in a health facility without injectable quinine in stock).

An analysis of 62 patients prescribed AL (whether or not AL was indicated according to guidelines) revealed that health workers almost always dosed AL correctly, but the quality of counseling was mixed (Table 33). Altogether, 95.1% of patients received AL that was correctly dosed. However, only 10.7% of patients were given the first dose of AL during the consultation. Although a large majority (88.2%) of patients were given complete dosing instructions (definition of a dose, number of doses per day, and treatment duration), fewer patients (60.9%) could repeat all the dosing instructions given by the health worker. Only 69.9% of patients were advised to complete all the treatment, and a lower proportion of patients were advised to take AL with food (31.3%), take AL with milk or fat-containing food (4.9%), return for a follow-up visit (14.4%), and return to the facility if the patient becomes seriously ill (5.8%). No patient was advised to sleep under a bed net to prevent malaria.

Most (78.4%) patients reported being very satisfied with the services they received at the health facility (Table 34), although we recognize that assessing patient satisfaction in the health facility might be positively biased. When asked, few patients expressed a specific thing that would improve the care at the health facility.

A rapid assessment of patients' malaria-related knowledge and reported practices showed that only about one-third of patients knew that people get malaria from mosquitoes and that one should sleep under a bed net to protect against malaria (Table 35). A slightly higher proportion (40.5%) of patients reported sleeping under a bed net. It is possible that these results underestimate true knowledge levels and practices, as shy respondents might have been reluctant to answer the questions. It is also possible that reported bed net use was overestimated, as respondents might have been trying to give an answer that would please the interviewer.

Graphical pathway analysis of the case management process

To link results of individual steps of the case-management process and identify strengths and weaknesses of health worker practices, we performed a graphical pathway analysis. To simplify the graphs, percentages are unweighted. The standard for this analysis was the old (pre-September 2007) policy. Among the 40 patients without febrile illness/suspected malaria (and therefore no malaria), we found that most (35/40, or 87.5%) patients were not tested for malaria, nearly all (34/35, or 97.1%) of these untested patients were not diagnosed with malaria; and of the 34 patients without a malaria diagnosis, none were treated with an antimalarial (Figure 5a, steps along the bottom of the figure). In other words, most patients in this group were managed correctly at all points in the case-management process. Additionally, although numbers of patients were very small, it is worth noting that among the 4 patients with a negative test result, 2 were given a diagnosis of malaria and the other 2 were not.

Among the 78 patients with febrile illness/suspected malaria but no gold standard malaria diagnosis, many (36/78, or 46%) were not tested even though they should have been (Figure 5b). Among the 41 patients with a negative test result, a majority (24/41, or 58.5%) were diagnosed with malaria. Regarding treatment, nearly all (33/36, or 91.7%) patients diagnosed with malaria

were prescribed an effective antimalarial; and of the 42 patients without a malaria diagnosis, none were treated with an antimalarial.

Among the 59 patients with febrile illness/suspected malaria and who actually had a gold standard malaria diagnosis, most (42/58, or 72.4%) were not tested even though they should have been (Figure 5c). Of the 26 patients who did not receive an antimalarial (a major error), none had been tested. All 16 patients with a positive malaria test were diagnosed with malaria. Regarding treatment, a large majority (31/35, or 88.6%) of patients diagnosed with malaria were prescribed an effective antimalarial; and of the 24 patients without a malaria diagnosis, none were treated with an antimalarial.

In summary, many patients who should have been tested were not, and this failure led to many incorrect diagnoses. Health workers did not trust negative test results: over half of patients with a negative test result were diagnosed with malaria. Health workers did trust positive test results, as all patients with a positive test were diagnosed with malaria. Prescribed treatments closely matched health worker diagnoses, and AL was usually dosed correctly.

Design effects and intraclass correlation coefficients

The design effects and intraclass correlation coefficients (ρ, or "rho") were examined for 25 key patient-level indicators of case-management quality (Table 36a). One indicator was excluded because the indicator value was zero, and thus neither the design effect nor ρ could be estimated. Among the remaining 24 indicators, the weighted design effects ranged from about 1.0 (indicating no correlation) to 3.4 (indicating moderate correlation); the median was 1.7 (Table 36b). Weighted values of ρ ranged from just under zero to about 1.0; the median was 0.3. Interestingly, the design effects for the two indicators of correct malaria treatment were close to one (i.e., 1.1 and 1.6), which reflects a relatively low amount of correlation.

As heterogeneous analysis weights have a tendency to increase design effects and decrease precision [Kalton et al., 2005], an unweighted analysis was performed to examine the effect of heterogeneous weights (final analysis weights ranged from 1.7 to 40.6). As expected, the unweighted values of the design effect and ρ were usually lower than values from the weighted analysis. An examination of the ratio of weighted design effects to unweighted design effects revealed a median ratio of 1.48. In other words, a typical impact of heterogeneous weights in the survey increased the design effect by 48%. This increase in design effect then led to an increase in the width of the 95% CI by 22% (i.e., the square-root of 1.48 is about 1.22)[34].

DISCUSSION

Case management with diagnostic testing and treatment with ACTs is a pillar of malaria control; yet despite enormous investments to implement it, remarkably few rigorous studies have evaluated the success of scale-up efforts [Zurovac et al., 2005; Zurovac et al., 2007; Zurovac et

[34] Design effects >1 reflect an increase in variance, variance equals the standard error^2, and 95% CIs increase proportionately with the standard error. So, for example, if the design effect quadrupled, the standard error would double (because the square-root of 4 is 2); and if the standard error doubled, then the 95% CI would double.

al., 2008a; Zurovac et al., 2008b; Skarbinski et al., 2009]. In Huambo Province, ACTs have been supplied intermittently since 2003, and large-scale implementation of the ACT policy began in August 2006. Therefore, this survey (conducted from October–November 2007) evaluated scale-up activities over a 14-month period. As the implementation plan had a multi-year time frame, by the time of the survey, not all activities had been completed. Since the survey, the following has occurred (personal communication from Rachel Shaw, The MENTOR Initiative, January 27, 2009): AL and RDTs have been distributed to more than 95 health facilities; a supervisory checklist was developed and tested; 379 supervisory visits were completed; 410 health workers were trained on RDT use, drug management, intermittent preventive treatment of malaria in pregnancy, and the use of AL and quinine; 24 laboratory technicians were trained to diagnose malaria with microscopy and RDTs; and communication activities disseminated malaria messages at the community level (i.e., five radio spots, 19 theatre presentations, and 94 community health presentations). As a result of these activities, the quality of case management at the present time is probably better than what is reflected in the survey results. However, the strengths and weaknesses identified in the survey are notable; and the results reveal problems that might exist today. In summary, this survey provides a wealth of practical information on malaria-related policies, health facility readiness, and case-management quality after recent scale-up efforts in a setting with a weak infrastructure that is typical of many parts of the developing world. Therefore, while our quantitative results only apply to Huambo Province, the broader lessons might be relevant to the rest of Angola and other low-income countries.

Our first main finding was related to the fact that when a policy is not clearly written and communicated, confusion can ripple throughout the health system. In Angola, parts of the NMCP policy document, and the subsequent announcement of a key policy modification, lacked clarity. In Huambo, local staff did their best to respond by adding details in training materials to fill gaps and clarify the clinical guidelines that health workers were expected to follow. However, despite these good intentions, the result was that nearly everyone involved in scale-up activities (and by extension, this evaluation team) had a different interpretation of the guidelines. On a related matter, the definition of suspected malaria seems to be unnecessarily complex, especially for under-5s (remarkably, no health worker could repeat the full definition).

The second finding was that many key health facility supports for case management were in place, although some additional strengthening was needed. All facilities had AL in-stock, the ability to perform malaria testing, and health workers on staff with AL training. Although we recognize that not all planned training had occurred and that supervision had just begun, aspects of health facility preparedness that we observed requiring improvement were: oral and injectable quinine were frequently not in-stock during survey visits, few facilities had all antimalarials in-stock continuously in the 3 months before the survey, one-quarter of patients were seen by health workers without AL training, and supervision on AL use was uncommon and often did not involve observation of consultations with feedback. In addition, our knowledge assessment of health workers revealed two major deficiencies: no one knew the full definition of which cases should be tested (although most knew which patients in case scenarios needed testing), and most health workers did not trust negative test results.

As implementation of IMCI had not occurred in Huambo by the time of the survey, it was not surprising that very few health workers had received IMCI training. However, the relevance

of IMCI is worth noting because IMCI guidelines recommend that health workers systematically identify and treat all potentially life-threatening childhood illnesses (e.g., in malarious areas, a child with fever, cough, and fast breathing is treated for both malaria and pneumonia). In contrast, malaria-specific training courses place less emphasis on this process, which might cause important illnesses to be missed and might lead to the unnecessary use of antimalarials among patients who have a negative malaria test. For example, if a patient with fever tests negative and the health worker has not checked for other causes of the fever, the worker might prescribe an antimalarial unnecessarily, just to have something to give the patient.

The third main finding (or group of findings) is related to case-management quality. In many ways, these results were the most important, as it is through the adherence to guidelines that ultimately lives are saved and resources, such as drugs and diagnostic tests, are efficiently used. As many indicators are needed to describe case-management quality comprehensively, we constructed a causal diagram to display the key malaria-related case-management practices and show the causal chain of observed problems, based on survey results (Figure 6). Clearly, this diagram is simplistic, as it omits many fundamental environmental factors of potential importance, such as low salaries, poor motivation, weak infrastructure, perceived patient expectations, and the low educational level of many health workers. For example, anecdotally, one NMCP official involved with training cited the low educational level of health workers as being a principal difficulty faced by trainers.

As illustrated at the top of Figure 6, the case-management process begins with patient assessment. We found that except for the determination of a history of fever, assessments were often incomplete. In particular, health workers measured the patient's temperature in only one-quarter of consultations and checked for anemia even less often.

Regarding malaria laboratory testing, under-use was very common, but over-use was not a large problem. We performed an in-depth analysis of health worker testing practices for patients needing a test because testing and trusting the result was the key to making a correct diagnosis and correct diagnosis was the key to prescribing the appropriate treatment. Interestingly, the factors significantly associated with correct testing were health worker caseload and patient's temperature (shown as root causes on the left side of Figure 6). These results illustrate the importance of non-intervention environmental factors [Rowe et al., 2005] and could be used to guide future quality improvement activities (e.g., changing schedules to reduce high caseloads, and teaching health workers to test all patients with a history of fever—not just those with an elevated temperature at the time of the consultation). Training on AL use had a borderline significant association with correct testing; but even if statistical significance is ignored, the absolute proportion of patients tested by health workers exposed to AL training was low (38.1%). Supervision on AL was not associated with testing, perhaps because supervision was primarily focused on pharmaceutical management rather than clinical practice. It was also notable that the health worker knowledge score was not associated with correct testing, which might explain the modest effect of training. The lack of association between health worker knowledge and practice has been observed in other settings [Ofori-Adjei & Arhinful, 1996; Paredes et al., 1996; Rowe et al., 2000], and this phenomenon should make program managers consider other quality improvement strategies besides training.

The quality of malaria diagnosis and treatment followed similar patterns to those seen for testing. Compared to our two analysis gold standards, only half to two-thirds of patients were correctly diagnosed and treated. For diagnosis, the most common errors were missing malaria cases and over-diagnosing malaria among patients not meeting the case definition. Part of the over-diagnosis can be explained by health workers' apparent distrust of negative test results, which echoes the results of the knowledge assessment. The erroneous diagnoses then translated into erroneous treatments because, in general, health workers correctly treated the diagnoses that they made. In addition, health workers rarely administered the first dose of AL during the consultation, and many aspects of counseling needed improvement. An analysis from the patient's perspective, which was perhaps the single most important indicator of health care quality (i.e., did patients needing malaria treatment according to the national policy leave the health facility with the medicine and the knowledge how to administer it at home?), found that only about one-third of patients received adequate (i.e., life-saving) care.

There were several important positive findings related to AL treatment. For example, it was reassuring that the large majority of patients that health workers diagnosed with malaria were prescribed AL at the correct dose; and when AL was prescribed, health workers accurately provided dosing instructions to patients. Also, there was little use of ineffective and non-recommended antimalarials—a problem that has been observed in other settings [Skarbinski et al., 2009; Wasunna et al., 2008; Zurovac et al., 2005].

Regarding the accuracy of laboratory testing, our survey had too small a sample size to provide a precise answer. Among the 27 patients tested by surveyors with microscopy (our gold standard for evaluating malaria diagnostics) <u>and</u> by health workers (with RDT or microscopy), the sensitivity of health worker testing was 2/2, and the specificity was 19/25 (76.0%, unweighted).

The fourth main finding was that the prevalence of parasitemia among patients with suspected malaria was very low (3.4%; 95% CI: 0–8.5). Two possible explanations are that the result was for non-pregnant patients $\geq$5 years old, who typically are less likely to have malaria as a cause of febrile illness than under-5s and pregnant women, and that the survey was conducted early in the rainy season, before malaria transmission had peaked. Still, low prevalence raises questions about the importance of malaria as a cause of illness in Huambo Province.

Methodologic lessons

This survey provided several methodologic lessons and raised some important questions. First, our survey team struggled with the definition of the gold standard against which case-management quality should be evaluated. Should the definition be based on what is written in a policy document, which was still in draft form; the understanding of the policy according to verbal explanations of NMCP officials; or what was taught in health worker training courses in the survey setting? We ultimately performed two parallel analyses to take the perspective of what had been taught to health workers, and then (as the policy itself was evolving) what NMCP officials wanted the policy to be—even though it was not yet written into a document.

The second lesson concerns sampling patients with the “follow the patient” method. While this method does allow surveyors to record aspects about all parts of the patient’s visit to the health facility and reduces the chance that a patient would be lost after enrollment, clearly we missed many patients because of an insufficient number of surveyors. In our case, another disadvantage was that the method led to widely varying analysis weights, which reduced precision. The alternate approach, which is probably better, is to have surveyors at fixed stations (e.g., observers who spend the entire visit observing consultations). The disadvantage of the fixed-station method is that a small survey team might not be able to observe all consultations in large facilities with several consultation rooms. The solution to this problem is to either randomly select consultation rooms (and record the sampling fraction, so results can be appropriately weighted) or determine in advance the number of consultation rooms in each sampled facility and create a small pool of extra surveyors who can be called to work at the larger facilities. The latter approach worked well in a series of sub-national health facility surveys in Benin [Rowe et al., 2009].

The third lesson relates to choosing which patients to test. Our protocol attempted to minimize the number of patients tested. We did this to prevent unnecessary patient discomfort and reduce the time patients spent with surveyors. This decision was short-sighted. Future surveys should test all patients, as it provides a parasitologic gold standard that is valuable for evaluating case-management quality and it allows for the estimation of the proportion of fever cases truly caused by malaria parasites.

Fourth, this survey demonstrated the positive bias that can result when health care quality is evaluated by measuring how well prescriptions match health worker diagnoses. With this approach, health care quality seemed excellent. Most (83.6%) patients whom health workers diagnosed with uncomplicated malaria were prescribed AL, and nearly all (97.3%) patients whom health workers did not diagnose with malaria were not prescribed an antimalarial[35]. In contrast, when health care quality was assessed with a gold standard based on an expert re-examination, the results were much lower.

Fifth, the survey revealed both strengths and weaknesses in the ability of our health worker knowledge assessment to be an accurate proxy for observed health worker practices. Results of the knowledge assessment correctly identified that: a) health workers did not trust negative malaria test results, b) this distrust led to unnecessary prescriptions of AL, and c) in general, health workers correctly treated the diagnoses they made. In contrast, although the knowledge assessment showed that most health workers could identify which patients needed malaria testing, in practice health workers did not test most of the patients that needed it.

Finally, as the survey was done in the midst of implementation, the results must be available promptly if they are to have practical value. The long delay in preparing this report should not be acceptable. Potential methods that could shorten the time from data collection to reporting results are cross-sectional surveys that use handheld computers for data entry [Thwing et al., 2009] or continuous surveys that report results monthly [Rowe, 2009].

[35] The small exception was two patients whom health workers diagnosed with complicated malaria but prescribed AL—a major error.

In addition to these methodologic lessons, the survey raised a fundamental question: when is the best time to evaluate scale-up efforts? As illustrated conceptually in Figure 7, scale-up has an initial phase when basic activities are implemented (e.g., delivering commodities and training) and a maintenance phase when quality might improve, stabilize, or decline—depending on the effectiveness of support strategies (e.g., supervision) and other factors [Rowe et al., 2005]. This survey was conducted during the initial phase, although about 14 months after scale-up efforts began. So perhaps it should not be surprising that some aspects of case management were weak. The benefit of conducting a survey at this time is that the results can be used to refine subsequent initial and maintenance scale-up activities. If the initial scale-up phase is expected to be lengthy or unusually expensive, or if there are serious concerns about the effectiveness of the scale-up approach, then a survey during initial scale-up might be quite valuable. The disadvantage is that the results risk "unfairly" portraying scale-up efforts negatively. A survey at the end of the initial phase evaluates initial scale-up activities (more fairly than during initial scale-up), and results can be used to refine maintenance phase activities. The advantages of a survey at this point are that it probably provides a less (negatively) biased characterization of initial scale-up and that it is easier to attribute weaknesses to real problems needing attention (i.e., weaknesses identified in a survey during initial scale-up could be attributed to the fact that scale-up simply had not been completed). Surveys even later (during the maintenance phase) evaluate the net effect of initial and maintenance scale-up activities and provide information that can inform the design of future maintenance phase interventions.

Recommendations

Based on the evidence from this survey and acknowledging the implementation activities that have occurred since the survey, we recommend the following.

1) **Regarding policy.** A committee of technical experts from the NMCP and other partners involved in malaria control should carefully review the NMCP's case-management policy document, clarify the guidelines, finalize and disseminate the document, and work with program and training experts to develop clear, easy-to-use educational materials (including a diagram of the algorithm) for training provincial managers, supervisors, and health workers. The dosing guidelines for AL should be revised so the weight categories have no gaps or overlaps (i.e., 5.0–14.9 kg, 15.0–24.9 kg, 25.0–34.0 kg, and >34.0 kg). One particular point to examine is the definition of which patients need malaria testing (i.e., the definition of suspected malaria). For all patients, the committee should consider slightly changing the temperature threshold to be "≥37.5ºC" instead of ">37.5ºC", to match WHO's IMCI guidelines. For under-5s (and perhaps older patients, too), the committee should consider defining suspected malaria as simply a history of fever or a measured axillary temperature ≥37.5ºC, as is done in IMCI guidelines (i.e., drop the part of the definition that includes ≥3 non-fever symptoms).

2) **Regarding drug management.** Efforts should be made to improve the drug management system to avoid stock-outs of antimalarials. It is especially important to focus on the availability of oral quinine and drugs for severe malaria (i.e., injectable quinine or artesunate, or artemether suppositories), which might have been somewhat ignored during the scale-up of ACTs.

3) **Regarding staff management.** Health facility directors should schedule health workers with AL training to work on weekdays during regular hours, when many patients with suspected malaria are seen.

4) **Regarding training.** Many health workers have been trained since the survey, therefore it is likely that many of the training gaps identified in the survey have already been filled. Additionally, since the survey, IMCI is now a priority. In any case, the existing training plan should be implemented, and health workers should be encouraged to disseminate their new knowledge and skills among colleagues who have not received formal training. As the importance of differential diagnosis grows as malaria intervention scale-up proceeds (because progressively fewer cases of fever will be caused by malaria parasites), integrated training (e.g., IMCI) should continue to be supported. Training materials should also be reviewed (and revised, if needed) to ensure that they are appropriate for the educational level of the health workers that will use them.

5) **Regarding supervision.** As with training, above, because substantial amounts of supervision have occurred since the survey, it is likely that many of the supervision gaps identified in the survey have been filled. Therefore, today, the key is to evaluate the quality of case management (see recommendation 9, below) and either continue the current supervision plan (if all is going well) or modify the current plan (if additional improvements are needed). As supervision is an excellent way to provide targeted training and motivation, model correct performance, and engage in problem-solving, all health workers who perform consultations should receive regular supportive supervision with the observation of consultations and constructive feedback. Supervisors themselves should be supported to give them a high level of technical and interpersonal skills. Although supervision should not target malaria case management exclusively (it is preferable to support health workers in more comprehensive, integrated case-management approaches), when malaria is the topic at hand, supervision should focus on: performing complete patient assessments, identifying which patients need malaria testing, building trust in diagnostics so patients with a negative test are not treated with an antimalarial, identifying complicated malaria cases, and improving counseling (e.g., health workers should systematically ask patients, or caretakers of patients, to repeat all counseling messages to verify comprehension) and patient education (e.g., teach patients that mosquito bites cause malaria and then remind patients to sleep under an insecticide-treated net). Health workers who perform well should be supervised at least once every three months, and workers with important deficiencies should be visited more often. There should also be consideration of training and supporting particularly high-performing and well-respected health workers to perform supervision in their health facilities or in nearby facilities.

6) **Regarding guideline adherence.** As mentioned above, current case-management quality should be evaluated. If weaknesses are identified, the NMCP and other partners should work with the provincial health management team to strengthen strategies to improve health worker adherence to the guidelines on malaria case management (ideally, within the context of a more comprehensive case-management approach). In addition to clear guidelines (see recommendation 1) and supervision (see recommendation 5), many possible interventions

exist, such as job aids, incentives, targeted training, and the quality improvement process, to name a few. If appropriate, consider seeking external technical assistance.

7) **Regarding laboratory testing.** The National Malaria Control Strategy should be fully implemented, including the change to presumptive treatment for under-5s, and microscopy and RDT training by national experts with cascade training at the regional and local level. Specifically, we recommend that the national policy be explained precisely in a finalized document, clear and focused training on when to test (emphasizing that all patients $\geq$5 with a history of fever should be tested, even if the temperature is not elevated) and what to do with the results (especially for negative results), emphasizing the importance of differential diagnoses (i.e., malaria is not the only cause of febrile illness), rapid establishment of a quality control system for RDTs and microscopy, continued supervision of both laboratorians and clinicians, and the inclusion of clinicians in some laboratory training (as clinicians sometimes perform malaria testing). In addition, supervisors should work with staff at high-volume clinics to implement strategies to keep the health worker caseload to <25 patients per day (e.g., by scheduling an extra health worker during busy times) and to avoid the practice of reporting malaria test results in batches (i.e., results should be reported as soon as they are ready).

8) **Regarding care-seeking.** The behavioral change component of malaria control efforts (e.g., activities on information/education/communication and behavioral change communication) should promote prompt care-seeking for people in the community with a febrile illness. Prompt care-seeking means visiting a health facility within 24 hours of fever onset. Some communication activities have occurred since the survey, which is a good start. We recommend a coordinated campaign with PMI working with the Ministry of Health, the provincial health management team, WHO, and other partners.

9) **Regarding future evaluation.** Some sort of follow-up evaluation should be performed to determine the degree to which activities over the past year (and actions taken based on these recommendations) were successful, as well as to identify new issues that need attention. The follow-up evaluation need not be a rigorous survey, but it should examine some of the indicators from the present survey, such as: a) the proportion of patients needing testing that are tested, b) the proportion of patients with suspected malaria who are truly parasitemic, c) the proportion of patients meeting the definition of malaria who are correctly diagnosed and treated, d) the proportion of treated cases who receive appropriate counseling, and e) the proportion of patients meeting the definition of malaria who leave the health facility with the correct medicines and demonstrated knowledge on how to administer the medicines at home. In addition, as implementing guidelines is a dynamic process, the NMCP and provincial health managers should consider an inexpensive approach for routinely monitoring indicators on key aspects of case management such as drug stocks, use of diagnostic tests, and case-management quality—perhaps with data collected by supervisors during their routine visits.

Limitations

The survey has several important limitations. First, we only studied health facilities in which ACT implementation activities had occurred. Therefore, we cannot generalize results to facilities where many patients might be seeking care—especially government-run health posts and private-sector facilities. Second, the participation rate was low because the patient flow at many facilities (especially bottlenecks at laboratories) led to long delays in completing patient visits that tied up surveyors and caused them to miss eligible patients. However, it does not seem likely that missing these patients introduced a large bias. Third, the direct observation of consultations could have caused health workers to be somewhat more careful than usual [Leonard 2006; Rowe 2002], which would have led to an overestimation of case-management quality. When we found high levels of performance, one might question our conclusions if quality was routinely lower in the absence of observers. However, when we found low levels of performance, the positive bias of observation would imply that regular performance was even lower; and so our conclusions would not be effected. Fourth, due to the design of the survey protocol, some patient groups with suspected malaria were not tested. This issue, along with the fact that the rains (and thus malaria transmission) began late, led to a patient sample size that was too small to evaluate the performance characteristics of microscopy and RDTs adequately. Lastly, we acknowledge some ambiguity in the gold standard diagnosis and treatment for cases in which health workers either tested a patient that should not have been tested or did not test a patient who should have been tested, although our general conclusions about case-management quality were unlikely to have been affected.

Conclusions

Huambo Province, Angola, is a challenging place to scale-up a new case-management policy that recommends a new drug, emphasizes diagnostic testing, and introduces a new test. The scale-up process has clearly been started; and the NMCP, development partners, local implementers, and health facility staff should be congratulated. Indeed, the survey had several important positive findings. Most patients whom health workers diagnosed with malaria were prescribed AL at the correct dose, AL dosing instructions were accurate, and ineffective and non-recommended antimalarials were rarely used. However, while substantial progress has been made to implement the case-management policy, important gaps were found. In particular, the lack of a clear policy, under-use of malaria diagnostic testing, and distrust of negative test results led to many incorrect malaria diagnoses and inappropriately treated patients. The strengths and weaknesses identified in this evaluation directly led to practical recommendations, including the need for the development and dissemination of a clear policy and training materials, improved drug and staff management, increased adherence to clinical guidelines, strengthened laboratory diagnostic practices, and a follow-up evaluation to determine whether actions taken were successful. These recommendations should be implemented promptly. Although health facility surveys like ours can seem to have a narrow geographic and temporal scope, the findings can inform public health activities elsewhere. We believe the lessons from this evaluation might apply to other parts of Angola and other low-income countries.

REFERENCES

Angola National Malaria Control Program. 2007. *Normas de manejo de casos de malária em Angola* [Standards for managing malaria cases in Angola]. Document is labeled as a draft and has two dates: 2005 and January 10, 2007.

Consultoria de Serviços e Pesquisas (COSEP) Lda., Consultoria de Gestão e Administração em Saúde (Consaúde) Lda. Angola, and Macro International Inc. 2007. *Angola Malaria Indicator Survey 2006–07*. Calverton, Maryland: COSEP Lda., Consaúde Lda., and Macro International Inc.

Gove S for the WHO Working Group on Guidelines for Integrated Management of the Sick Child. Integrated management of childhood illness by outpatient health workers: technical basis and overview. Bulletin of the World Health Organization 1997; 75 (suppl 1): 7–24.

Kish L. *Survey Sampling*. New York: John Wiley & Sons, Inc., 1965, pages 162, 171, and 258.

Info-Angola, Projecto Portal do Governo. Available at: http://www.info-angola.com/governo/index.php?option=com_content&task=view&id=265&Itemid=202, accessed January 22, 2009.

Kalton G, Brick JM, Lê T. Estimating components of design effects for use in sample design. In: Household sample surveys in developing and transitional countries. Studies in methods, series F, no. 96. 2005. Statistics Division, Department of Economic and Social Affairs, United Nations. New York: United Nations. Document no. ST/ESA/STAT/SER.F/96.

Leonard K, Masatu MC. Outpatient process quality evaluation and the Hawthorne effect. Social Science and Medicine 2006; 63: 2330–2340.

Ofori-Adjei D, Arhinful DK. Effect of training on the clinical management of malaria by medical assistants in Ghana. Social Science and Medicine 1996; 42: 1169–1176.

Paredes P, Pena M, Flores-Guerra E, Diaz J, Trostle J. Factors influencing physicians' prescribing behavior in the treatment of childhood diarrhoea: knowledge may not be the clue. Social Science and Medicine 1996; 42: 1141–1153.

The President's Malaria Initiative. Internet website. Available at: http://www.fightingmalaria.gov/, accessed April 4, 2009.

Rowe AK, Hamel MJ, Flanders WD, Doutizanga R, Ndoya J, Deming MS. Predictors of correct treatment of children with fever seen at outpatient health facilities in the Central African Republic. American Journal of Epidemiology 2000; 151: 1029–1035.

Rowe AK, Lama M, Onikpo F, Deming MS. Design effects from a health facility cluster survey in Benin. International Journal for Quality in Health Care 2002; 14: 521–523.

Rowe AK, Lama M, Onikpo F, Deming MS. Health worker perceptions of how being observed influences their practices during consultations with ill children. Tropical Doctor 2002; 32: 166–167.

Rowe AK, Lama M, Onikpo F, Deming MS. Risk and protective factors for two types of error in the treatment of children with fever at outpatient health facilities in Benin. International Journal of Epidemiology 2003; 32: 296–303.

Rowe AK, de Savigny D, Lanata CF, Victora CG. How can we achieve and maintain high-quality performance of health workers in low-resource settings? Lancet 2005; 366: 1026–1035.

Rowe AK, Onikpo F, Lama M, Osterholt DM, Rowe SY, Deming MS. A multifaceted intervention to improve health worker adherence to Integrated Management of Childhood Illness guidelines in Benin. American Journal of Public Health 2009; 99: 837–846.

Rowe AK. Potential of integrated continuous surveys and quality management to support monitoring, evaluation, and the scale-up of health interventions in developing countries. American Journal of Tropical Medicine and Hygiene 2009; 80: 971–979.

Skarbinski J, Ouma PO, Causer LM, Kariuki SK, Barnwell JW, Alaii JA, Macedo de Oliveira A, Zurovac D, Larson BA, Snow RW, Rowe AK, Laserson KF, Akhwale WS, Slutsker L, Hamel MJ. Effect of malaria rapid diagnostic tests on the management of uncomplicated malaria with artemether-lumefantrine in Kenya: a cluster randomized trial. American Journal of Tropical Medicine and Hygiene 2009; 80: 919–926.

Thwing JI, Mihigo J, Fernandes AP, Saute F, Ferreira C, Fortes F, de Oliveira AM, Newman RD. How much malaria occurs in urban Luanda, Angola? A health facility-based assessment. American Journal of Tropical Medicine and Hygiene 2009; 80: 487–491.

Wasunna B, Zurovac D, Goodman CA, Snow RW: Why don't health workers prescribe ACT? A qualitative study of factors affecting the prescription of artemether-lumefantrine. Malaria Journal. 2008; 7: 29.

World Health Organization. Training for Mid-level Managers: The EPI Coverage Survey. Geneva: WHO Expanded Programme on Immunization, 1991. WHO/EPI/MLM/91.10.

World Health Organization. Integrated Management of Adolescent and Adult Illness. Interim guidelines for first-level facility health workers at health centre or district out patient clinic. Geneva: World Health Organization, 2004. Document WHO/CDS/IMAI/2004.1 Rev. 1.

Zurovac D, Ndhlovu M, Rowe AK, Hamer DH, Thea DM, Snow RW. Treatment of paediatric malaria during a period of drug transition to artemether-lumefantrine in Zambia: cross-sectional study. British Medical Journal 2005; 331: 734–737.

Zurovac D, Ndhlovu M, Sipilanyambe N, Chanda P, Hamer DH, Simon JL, Snow RW. Paediatric malaria case-management with artemether-lumefantrine in Zambia: a repeat cross-sectional study. Malaria Journal 2007; 6: 31.

Zurovac D, Njogu J, Akhwale W, Hamer DH, Snow RW. Translation of artemether-lumefantrine treatment policy into paediatric clinical practice: an early experience from Kenya. Tropical Medicine and International Health 2008; 13: 99–107.

Zurovac D, Tibendarana JK, Nankabirwa J, Ssekitooleko J, N Njogu JN, Rwakimari JB, Meek S, Talisuna A, Snow RW. Malaria case-management under artemether-lumefantrine treatment policy in Uganda. Malaria Journal 2008; 7: 181.

Box 1. Definitions of signs of severe illness for all ages (same for “old” policy [pre-September 2007] and “new” policy [announced in September 2007])

1. Cerebral dysfunction and cerebral malaria in the guidelines was defined as: history of convulsions or observed convulsions, lethargy, or unconsciousness.

2. Disseminated intravascular coagulopathy defined as: spontaneous bleeding or bruising.

3. Hemoglobinuria defined as: dark urine.

4. Hepatic dysfunction defined as: jaundice.

5. Hyperthermia defined as: temperature >41º C.

6. Pulmonary edema defined as: respiratory distress.

7. Renal insufficiency defined as: little or no urine.

8. Severe anemia defined as: severe palmar pallor.

9. Shock defined as: cyanosis, nail bed capillary refill >2 seconds, pulse that is weak and >110 beats/minute, systolic blood pressure <80 mm Hg (for adults only).

Note. While the following signs were in the national policy, they were not included in the survey analysis algorithm because it was not feasible to perform the laboratory tests in the field (and most health facilities could not perform the tests either): hyperparasitemia (>100,000 parasites/mm^3), hypoglycemia, hyponatremia, and metabolic acidosis.

Box 2. Definitions of other causes of febrile illness

1. Dysentery in the guidelines was defined as: diarrhea and bloody stools.

2. Hepatitis defined as: jaundice.

3. Influenza-like illness defined as: nasal or sinus congestion.

4. Measles defined as: measles rash or Koplic spots.

5. Otitis defined as: ear pain.

6. Pneumonia defined as: cough and either fast breathing or respiratory distress. Fast breathing was defined as respiratory rates of ≥50 breaths/minute for ages <12 months, ≥40 breaths/minute for ages 12–59 months, ≥30 breaths/minute for ages 5–13 years, and ≥20 breaths/minute for ages >13 years.

7. Urinary tract infection defined as: difficult or painful urination.

Box 3. Dosage for artemeter-lumefantrine (AL) used in the analysis

- Weight <5 kg: AL not recommended
- Weight 5.0–14.9 kg: 1 pill twice a day for 3 days
- Weight 15.0–24.0 kg: 2 pills twice a day for 3 days
- Weight 24.1–34.9 kg: 3 pills twice a day for 3 days
- Weight ≥35.0 kg: 4 pills twice a day for 3 days

Table 1. Health facility type and municipality in a health facility survey in Huambo Province, Angola, October–November 2007

Characteristic [question/variable in dataset]	All eligible[a] health facilities in Huambo Province (N=57)		Health facilities in the sample (N=33)	
	No.	%	No.	%
Health facility type[b] [F5]				
Hospital	8	14.0%	5	15.2%
Health center-adequate infrastructure	23	40.4%	13	39.4%
Health center-low infrastructure	25	43.9%	15	45.5%
Health post	1	1.8%	0	0%
Municipality (i.e., district) where The health facility is located [F3]				
Bailundo	4	7.0%	3	9.1%
Caala	6	10.5%	4	12.1%
Ekunha	3	5.3%	2	6.1%
Huambo	18	31.6%	10	30.3%
Katchihungo	3	5.3%	1	3.0%
Londuimbali	5	8.8%	4	12.1%
Longonjo	4	7.0%	1	3.0%
Mungo	3	5.3%	2	6.1%
Tchickala Tcholohanga	4	7.0%	2	6.1%
Tchinjenje	3	5.3%	2	6.1%
Ukuma	4	7.0%	2	6.1%

Footnotes for Table 1.

[a] At the time of the survey, there were 57 health facilities where artemisinin-based combination therapy (ACT) had been implemented (defined as having at least 1 health worker trained in the diagnosis and treatment of malaria with the ACT case-management policy and the delivery of ACTs at least once in the past).

[b] Health centers with adequate infrastructure are larger outpatient health facilities with a laboratory and inpatient ward, and where antenatal consultations are performed. Health centers with low infrastructure are larger outpatient health facilities that lack at least one of the following: a laboratory, an inpatient ward, or antenatal consultations. Health posts are smaller health facilities.

Table 2. Estimated numbers[a] of health workers (HWs) and their training related to malaria case management, extrapolated to all 57 health facilities (HFs) in Huambo Province, Angola, October–November 2007

Health worker category [question/ variable in dataset]	Estimated no. of HWs in all 57 HFs in Huambo, rounded (median no. per HF)	Estimated no. of HWs who received formal in-service training on topics related to malaria case management			
		Training on the use of ACTs[b] (%)	Training on the use of malaria RDTs[b] (%)	Training on IPTp (%)	Training on IMCI guidelines (%)
Physician [F11a1x – F11a5x]	26 (0)	3 (11.5%)	3 (11.5%)	0 (0%)	7 (26.9%)
Nurse [F11b1x – F11b5x]	2577 (24)	668 (25.9%)	698 (27.1%)	328 (12.7%)	54 (2.1%)
Midwife [F11c1x – F11c5x]	326 (4)	152 (46.6%)	161 (49.4%)	226 (69.3%)	5 (1.5%)
Health assistant or nursing aid [F11d1x – F11d5x]	181 (0)	133 (73.5%)	128 (70.7%)	100 (55.2%)	12 (6.6%)
Lab worker [F11e1x – F11e5x]	169 (3)	57 (33.7%)	83 (49.1%)	16 (9.5%)	0 (0%)
Other [F11f1x – F11f5x]	285 (0)	12 (4.2%)	12 (4.2%)	12 (4.2%)	2 (0.7%)
Total	3564	1025	1085	682	80

Footnotes for Table 2.

ACT = artemisinin-based combination therapy; IMCI = Integrated Management of Childhood Illness; IPTp = intermittent preventive treatment of malaria during pregnancy; RDT = rapid diagnostic test.

[a] Total number of health workers assigned to the facility, not just the number of health workers at the facility on the day of the survey visit.

[b] Use of ACTs and RDTs was generally taught in the same in-service training course.

Table 3. Percentage of health facilities with at least one health worker, by category and their training related to malaria case management, Huambo Province, Angola, October–November 2007

Health worker category [question/ variable in dataset]	Percentage of health facilities with at least one health worker in the category	Percentage of health facilities with at least one health worker in the category who received formal in-service training on topics related to malaria case management			
		Training on the use of artemisinin-based combination therapy	Training on the use of malaria rapid diagnostic tests	Training on intermittent preventive treatment of malaria during pregnancy	Training on Integrated Management of Childhood Illness guidelines
Physician [F11a1x – F11a5x]	18.2%	6.1%	6.1%	0%	9.1%
Nurse [F11b1x – F11b5x]	100%	100%	100%	51.5%	31.3%
Midwife [F11c1x – F11c5x]	81.8%	54.6%	54.6%	75.8%	6.1%
Health assistant or nursing aid [F11d1x – F11d5x]	18.2%	15.2%	15.2%	9.1%	9.1%
Lab worker [F11e1x – F11e5x]	60.6%	30.3%	45.5%	6.1%	0%
Other [F11f1x – F11f5x]	48.5%	3.0%	3.0%	0%	3.0%

Table 4. Equipment, supplies, and staffing related to malaria case management, Huambo Province, Angola, October–November 2007

Characteristic [question/variable in dataset]	Health facilities with the characteristic (N=33)	
	No.	% (95% CI)
Thermometer [F12a]	28	84.8% (76.8–92.9)
Functional scale for weighing children [F12b]	32	97.0% (93.1–100)
Booklet or chart with nationally recommended ACT treatment algorithms for children and adults [F12d]	16	48.5% (37.3–59.6)
Staff person who was trained to perform microscopy [F12f]	19	57.6% (46.5–68.6)
Functional microscope[a] [F12g]	19	57.6% (46.5–68.6)
Glass slides and Giemsa stain for at least 25 malaria smears [F12h]	16	48.5% (37.3–59.6)
Staff person who was trained to perform RDTs [F12i]	29	87.9% (80.6–95.2)
At least 25 valid (not expired) RDTs in stock [F12j]	31	93.9% (88.6–99.3)
Malaria testing available, by microscopy <u>or</u> RDT[b] [DX_AVAIL]	33	100% (89.4–100)
Both microscopy <u>and</u> RDTs available[c]	13	39.4% (28.5–50.3)

<u>Footnotes for Table 4.</u>

ACT = artemisinin-based combination therapy; CI = confidence interval; RDT = rapid diagnostic test.

[a] A microscope was considered functional if the microscopist said it was.

[b] Malaria testing was consider available if, on the day of the survey visit, the health facility had at least one of the following: 1) a microscopist <u>and</u> a functional microscope, or 2) a staff person trained to perform RDTs <u>and</u> at least 25 valid (not expired) rapid test cassettes.

[c] On the day of the survey visit, the health facility had <u>both</u> of the following: 1) a microscopist and a functional microscope, <u>and</u> 2) a staff person trained to perform RDTs and at least 25 valid (not expired) rapid test cassettes.

Table 5. Availability of bed nets and medicines in health facilities, Huambo Province, Angola, October–November 2007

Commodity [question/variable in dataset]	In stock on the day of the survey visit		At least 20 blister packs (not expired) in stock on the day of the survey visit		In stock every day for the past 3 months	
	n/N[a]	% (95% CI)	n/N[a]	% (95% CI)	n/N	% (95% CI)
Mosquito bed nets available at the facility for distribution [F13a]	0/32	0% (0–10.9)	NA		NA	
AL blister packs for patients 5–14 kg [F13a1, F13a2, F13a3]	26/33	78.8% (69.7–87.9)	24/32	75.0% (65.0–85.0)	7/33	21.2% (12.1–30.3)
AL blister packs for patients 15–24 kg [F13b1, F13b2, F13b3]	33/33	100% (89.4–100)	32/32	100% (89.1–100)	12/33	36.4% (25.6–47.1)
AL blister packs for patients 25–34 kg [F13c1, F13c2, F13c3]	33/33	100% (89.4–100)	31/32	96.9% (92.8–100)	12/33	36.4% (25.6–47.1)
AL blister packs for patients $\geq$35 kg [F13d1, F13d2, F13d3]	30/33	90.9% (84.5–97.3)	29/32	90.6% (83.9–97.4)	10/33	30.3% (20.0–40.6)
All four AL blister packs available	24/33	72.7% (62.8–82.7)	NA		4/33	12.1% (4.8–19.4)
Artesunate tablets [F13f1, F13f3]	0/33	0% (0–10.6)	NA		0/33	0% (0–10.6)
Amodiaquine tablets [F13g1, F13g3]	29/33	87.9% (80.6–95.2)	NA		23/33	69.7% (59.4–80.0)
Artemether (intramuscular injectable) [F13h1, F13h3]	0/33	0% (0–10.6)	NA		1/33	3.0% (0–6.9)
Artesunate (intravenous injectable) [F13i1, F13i3]	0/33	0% (0–10.6)	NA		0/33	0% (0–10.6)
Artemisinin suppositories [F13j1, F13j3]	0/33	0% (0–10.6)	NA		0/33	0% (0–10.6)
Artesunate suppositories [F13k1, F13k3]	0/33	0% (0–10.6)	NA		0/33	0% (0–10.6)
Quinine (tablets) [F13l1, F13l3]	12/33	36.4% (25.6–47.1)	NA		8/33	24.2% (14.7–33.8)
Quinine or quinidine (injectable) [F13m1, F13m3]	18/33	54.4% (43.4–65.7)	NA		11/33	33.3% (22.8–43.9)
Oral antibiotic (amoxicillin, ampicillin, cotrimoxazole, or erythromycin) [F13n1, F13n3]	31/33	93.9% (88.6–99.3)	NA		22/33	66.7% (56.1–77.2)
Iron [F13o1, F13o3]	27/33	81.8% (73.2–90.4)	NA		24/33	72.7% (62.8–82.7)
Oral rehydration solution packets [F13p1, F13p3]	23/33	69.7% (59.4–80.0)	NA		15/33	45.5% (34.3–56.6)

Footnotes for Table 5.

AL = artemether-lumefantrine; CI = confidence interval; NA = not available.

[a] Denominators that are less than 33 indicate at least one missing value.

Table 6. Formal[a] in-service training of health workers on topics other than the malaria case management policy recommending artemisinin-based combination therapy, Huambo Province, Angola, October–November 2007

Topic of the formal in-service training course[b] [question/variable in dataset]	Health workers who had received the in-service training (N = 93 health workers)		Year of training	Training duration, in days
	n	% (95% CI)	Median (range)	Median (range)
Diarrhea case management [E7b, E7b2, E7b3]	31	33.3% (20.1–46.6)	2005 (1989–2007)	5 (1–90)
Acute respiratory infection case management [E7c, E7c2, E7c3]	26	28.0% (16.5–39.5)	2005 (1992–2007)	5 (1–30)
Immunizations [E7d, E7d2, E7d3]	23	24.7% (12.9–36.6)	2005 (1986–2007)	3 (1–25)
Nutrition [E7e, E7e2, E7e3]	30	32.3% (20.1–44.4)	2005 (1992–2007)	5 (1–60)
Other[c] [E7f, E7g, E7h, E7other]	57	61.3% (48.5–74.0)	Not calculated	Not calculated

Footnotes for Table 6.

CI = confidence interval.

[a] Formal training means an organized course (e.g., in a classroom or clinical setting) that typically lasts at least a half day—not simply a short (e.g., 1 hour) impromptu educational session provided by a supervisor or peer.

[b] Results for training on Integrated Management of Childhood Illness (IMCI) are not shown because they were thought to be unreliable: the reported median training duration was only 5 days and ranged from 1 to 90 days (IMCI training is usually 11 days), and the year of training was sometimes as early as 1994 (IMCI was still being piloted in 1994). Presumably, health workers did not always understand what IMCI was.

[c] Numerous subjects were mentioned, such as "*Doentes Correntes*" (common illnesses such as diarrhea, respiratory illnesses, and malaria), essential medicines, breastfeeding, sexually transmitted illnesses/HIV/AIDS, obstetrics, and tuberculosis.

Table 7. In-service training of health workers on the malaria case-management policy for artemether-lumefantrine (AL), Huambo Province, Angola, October–November 2007

Training category [question/variable in dataset]	Health workers in the training category (N=93 health workers)	
	n	% (95% CI)
Health worker received at least one formal[a] training [E8a]	56	60.2% (47.7–72.8)
No. of formal training courses the health worker attended [E8b]		
0	37	39.8% (27.2–52.3)
1	36	38.7% (27.7–49.7)
2	12	12.9% (6.0–19.8)
3	7	7.5% (1.8–13.3)
4	1	1.1% (0–3.3)
Health worker received informal[a] training [E8f]	60	64.5% (53.1–75.9)
Health worker received any training (formal <u>or</u> informal) [E8a, E8f]	72	77.4% (66.6–88.2)
No. of formal and informal trainings received (no. of exposures[b])		
Neither formal nor informal training (0 exposures)	21	22.6%
One formal training and no informal training (1 exposure)	10	10.8%
Informal training and no formal training (1 exposure)	16	17.6%
Two formal trainings and no informal training (2 exposures)	1	1.1%
Informal training <u>and</u> one formal training (2 exposures)	26	28.0%
Three formal trainings and no informal training (3 exposures)	1	1.1%
Informal training <u>and</u> two formal trainings (3 exposures)	11	11.8%
Informal training <u>and</u> three formal trainings (4 exposures)	6	6.5%
Informal training <u>and</u> four formal trainings (5 exposures)	1	1.1%
Estimated total no. of days of AL training[c] [TR_DAYS_CAT]		
0 days (neither formal nor informal training)	21	22.6%
0.5 days	16	17.2%
1–3 days	13	14.0%
3.5 days	17	18.3%
4–6 days	12	12.9%
6.5–7.5 days	7	7.5%
9.5–45.5 days	7	7.5%

Footnotes for Table 7.

AL = artemether-lumefantrine; CI = confidence interval.

[a] Formal training means an organized course (e.g., in a classroom or clinical setting) that typically lasts at least a half day. Informal training is typically a short (e.g., 1 hour) impromptu educational session provided by a supervisor or peer.

[b] Each training (formal or informal) is considered one exposure. It is assumed that health workers with informal training only received it once.

[c] The method for estimating days of AL training (analysis variable TR_DAYS) was as follows. For formal training, assume that the duration of the last formal training represents all formal trainings (a reasonable assumption because 73 [78.5%] of 93 of health workers had either zero or one formal training); and for the 3 health workers with missing duration, assume duration of 3 days (by far the most common value). For informal training, assume a training duration of 0.5 days.

Table 8. Details of the formal in-service training on the malaria case management policy recommending artemether-lumefantrine received by 56 health workers, Huambo Province, Angola, October–November 2007

Training category [question/variable in dataset]	Health workers in the training category (N=56 health workers)	
	n	%
Formal training course included use of rapid diagnostic tests [E8c]	50	89.3%
Duration of most recent formal training course [E8e]		
1–2 days	13	23.2%
3 days	26	46.4%
4–5 days	11	19.6%
6–15 days	3	5.4%
Missing	3	5.4%
Year of most recent formal training course [E8d]		
2005	1	1.8%
2006	10	17.9%
2007	42	75.0%
Missing	3	5.4%
Course taught the "new" policy (announced in September 2007)[a] [NEW_GUIDE_TR]	3	5.4%

Footnote for Table 8.

[a] These results are outdated because training in the new policy was just beginning during the survey period.

Table 9. Supervision of health workers, Huambo Province, Angola, October–November 2007

Characteristic [question/variable in dataset]	No. and percent of health workers (N=93)	
	n	% (95% CI)
No. of times supervised in the past 6 months (any type of supervision, even if unrelated to malaria) [E9]		
0	25	26.9% (17.6–36.1)
1	26	28.0% (15.2–40.7)
2	19	20.4% (10.7–30.2)
3	14	15.1% (7.5–22.6)
4	5	5.4% (0.6–10.1)
5	4	4.3% (0.1–8.5)
No. of times supervised in the past 6 months in which the supervisor observed and provided feedback on a consultation [E10]		
0	61	65.6% (56.5–74.7)
1	21	22.6% (14.5–30.7)
2	9	9.7% (3.2–16.2)
3	1	1.1% (0–3.3)
5	1	1.1% (0–3.3)
No. of times supervised in the past 6 months on the use of AL [E10a]		
0	46	49.5% (39.3–59.6)
1	35	37.6% (27.4–47.9)
2	10	10.8% (4.2–17.3)
3	2	2.2% (0–5.3)

Footnotes for Table 9.

AL = artemether-lumefantrine; CI = confidence interval.

Table 10. Distribution of the total number of patients seen per health worker on the day of the survey visit, Huambo Province, Angola, October–November 2007

Caseload on the day of the survey visit [E12, CLOAD_CAT]	No. and percent of health workers (N=92 [1 missing])	
	n	%
0–4 patients	13	14.1%
5–9 patients	14	15.2%
10–14 patients	26	28.3%
15–19 patients	15	16.3%
20–24 patients	9	9.8%
25–29 patients	4	4.3%
30–34 patients	5	5.4%
35–39 patients	3	3.3%
40–44 patients	3	3.3%

Table 11. Knowledge assessment of health workers, Huambo Province, Angola, October–November 2007: Summarizing responses to an open-ended question on diagnostics: "Describe in as much detail as possible how you decide which patients should be tested for malaria with microscopy or with a rapid diagnostic test (such as Paracheck)."

Element used to code the open-ended question (see Annex 9 for details) [question/variable in dataset]	N=90[a] HWs
HW repeated the complete definition of suspected malaria cases to be tested according to the old policy[b] in Huambo [n (%)] [E13, E13_var1]	0 (0%)
Percentage of the criteria in the definition of suspected malaria cases to be tested according to the old policy in Huambo[c] [median percentage (range of percentages)] [E13_var2]	14.3% (0–42.9)
HW repeated the complete definition of suspected malaria cases to be tested according to the new policy[b] in Huambo [n (%)] [E13_var3]	0 (0%)
Percentage of the criteria in the definition of suspected malaria cases to be tested according to the new policy in Huambo[c] [median percentage (range of percentages)] [E13_var4]	14.3% (0–35.7)
HW mentioned fever in the response as a criteria for testing [n (%)] [E13_var5]	59 (65.6%)
HW mentioned "3 or more (*non-fever*) symptoms" in the response as criteria for testing [n (%)] [E13_var7]	0 (0%)
HW mentioned signs or symptoms that were not in the Huambo policy as criteria for testing (i.e., Are "extra" elements added[d])? [n (%)] [E13_var11]	33 (36.7%)
Age ranges mentioned by the HW in the response as criteria for testing [E13_var6]	
Less than 5 years old or "child" [n (%)]	7 (7.8%)
5 years old or older [n (%)]	1 (1.1%)
Both less than 5 year and 5 years and older [n (%)]	1 (1.1%)
Other age mentioned [n (%)]	0 (0%)
Age not mentioned [n (%)]	81 (90.0%)
HW mentioned that children < 5 years old should not be tested [n (%)] [E13_var9]	3 (3.3%)

<u>Footnotes for Table 11.</u>

HW = health worker.

[a] Responses of three HWs indicated that they did not understand the question, and the responses could not be coded (i.e., E13_var10 = No).

[b] The "old" policy is the pre-September 2007 policy, and the "new" policy began in September 2007.

[c] Each definition of suspected malaria cases had 14 criteria (e.g., fever, headache, etc.). This coded element of the HW's response is the percentage of these 14 criteria included in the HW's response (e.g., if a HW's response included 7 of the 14 criteria, the percentage would be 50%).

[d] The "extra" element not included in this analysis is "test patients who failed previous treatment," which is a reasonable response and not part of guidelines for managing a patient seen at an initial consultation.

Table 12. Knowledge assessment of health workers, Huambo Province, Angola, October–November 2007: Summarizing responses to a series of case-management scenarios

Scenario and responses [question/variable in dataset]	No. and percent of health workers (N=93)	
	n	%
**Scenario 1.** "A 30-year old man with fever (temperature is 39ºC), fatigue, and no other symptoms. A malaria RDT is negative." The correct diagnosis is unexplained fever/not malaria; regarding antimicrobials, the correct treatment is none needed; and hospitalization is not needed.		
Diagnosis (no. of health workers who gave the response) [E14a, E14a_cat]		
Responses considered correct: uncertain diagnosis (n=7), or fever of unknown origin (n=4), or more testing needed (n=2), or not malaria (n=1)	14	15.1%
Malaria (n=70), or suspected malaria (n=6)	76	81.7%
Typhoid fever (n=1)	1	1.1%
Do not know (n=2)	2	2.2%
Treatment (no. of health workers who gave the response) [E14b, E14b_cat]		
Response considered correct: antipyretics only (n=15)	15	16.1%
Coartem (n=65), or amodiaquine (n=3), or quinine (n=2), or unspecified antimalarial (n=2), or "Arinate" artemisinin monotherapy (n=1), or Coartem + antibiotic (n=1), or amodiaquine + antibiotic (n=1)	75	80.6%
Antibiotic only (n=2)	2	2.2%
Do not know (n=1)	1	1.1%
Is hospitalization necessary? [E14c]		
Response considered correct: No	80	86.0%
Yes	11	11.8%
Not sure	2	2.2%

Table 12 continued on next page.

Table 12, continued. Knowledge assessment of health workers, Huambo Province, Angola, October–November 2007: Summarizing responses to a series of case-management scenarios

Scenario and responses [question/variable in dataset]	No. and percent of health workers (N=93)	
	n	%
**Scenario 2.** "A 25-year old pregnant woman with fever (temperature is 38ºC), headache, and no other symptoms. She has been pregnant for 2 months. A malaria RDT is negative." The correct diagnosis is unexplained fever/not malaria; regarding antimicrobials, the correct treatment is none needed; and hospitalization is not needed.		
Diagnosis (no. of health workers who gave the response) [E15a, E15a_cat]		
Responses considered correct: uncertain diagnosis (n=14), or fever of unknown origin (n=3), or more testing needed (n=1), or not malaria (n=2)	20	21.5%
Malaria (n=64), or suspected malaria (n=3)	67	72.0%
Other diagnoses: urinary tract infection (n=3), hypertension (n=1)	4	4.3%
Do not know (n=2)	2	2.2%
Treatment (no. of health workers who gave the response) [E15b, E15b_cat]		
Response considered correct: antipyretics only (n=18), or no medicines (n=1), or refer to maternity (n=1)	20	21.5%
Quinine (n=36), or Coartem (n=12), or amodiaquine (n=9), or sulfadoxine-pyrimethamine (n=5), or unspecified antimalarial (n=2), or "quinine or amodioquine" (n=1), or chloroquine (n=1)	66	71.0%
Other medicines: antibiotic only (n=4), or captopril (n=1)	5	5.4%
Do not know (n=2)	2	2.2%
Is hospitalization necessary? [E15c]		
Response considered correct: No	83	89.2%
Yes	9	9.7%
Not sure	1	1.1%

Table 12 continued on next page.

Table 12, continued. Knowledge assessment of health workers, Huambo Province, Angola, October–November 2007: Summarizing responses to a series of case-management scenarios

Scenario and responses<br>[question/variable in dataset]	No. and percent of health workers (N=93)	
	n	%
**Scenario 3.** "A 41-year old man with fever (temperature is 39ºC), fatigue, and no other symptoms. Microscopy is negative for malaria." The correct diagnosis is unexplained fever/not malaria; regarding antimicrobials, the correct treatment is none needed; and hospitalization is not needed.		
Diagnosis (no. of health workers who gave the response) [E17a, E17a_cat]		
Responses considered correct: uncertain diagnosis (n=12), or fever of unknown origin (n=5), or more testing needed (n=3), or not malaria (n=2), or *gripe*/influenza-like illness (n=2)	24	25.8%
Malaria (n=63), or suspected malaria (n=5)	68	73.1%
Do not know (n=1)	1	1.1%
Treatment (no. of health workers who gave the response) [E17b, E17b_cat]		
Response considered correct: antipyretics only (n=20), or give fluids (n=1)	21	22.6%
Coartem (n=60), or amodiaquine (n=6), or quinine (n=2), or unspecified antimalarial (n=1), or "Coartem or amodiaquine" (n=1)	70	75.3%
Antibiotic only (n=2)	2	2.2%
Is hospitalization necessary? [E17c]		
Response considered correct: No	78	83.9%
Yes	14	15.1%
Not sure	1	1.1%

Table 12 continued on next page.

Table 12, continued. Knowledge assessment of health workers, Huambo Province, Angola, October–November 2007: Summarizing responses to a series of case-management scenarios

Scenario and responses [question/variable in dataset]	No. and percent of health workers (N=93)	
	n	%
**Scenario 4.** "A 32-year old woman with fever and fatigue. She had a convulsion in the morning, but is awake now. No other symptoms. Microscopy was positive for malaria." The correct diagnosis is severe malaria; regarding antimicrobials, the correct treatment is injectable quinine (and no anti-seizure medicines); and hospitalization is needed.		
Diagnosis (no. of health workers who gave the response) [E18a, E18a_cat]		
Responses considered correct: severe malaria (n=63), or "malaria" (severity not specified)[a] (n=28)	91	97.8%
Moderate malaria (n=1), or uncomplicated malaria (n=1)	2	2.2%
Treatment (no. of health workers who gave the response) [E18b, E18b_cat]		
Response considered correct: injectable quinine (n=66), or injectable quinine + antibiotic (n=2)	68	73.1%
Coartem (n=15), or injectable quinine + diazepam (n=4), or oral quinine (n=2), or amodiaquine (n=1), or unspecified antimalarial (n=1), or quinine (route of administration not specified) (n=1), or injectable artemether + antibiotic (n=1)	25	26.9%
Is hospitalization necessary? [E18c]		
Response considered correct: Yes	87	93.5%
No	6	6.5%

Table 12 continued on next page.

Table 12, continued. Knowledge assessment of health workers, Huambo Province, Angola, October–November 2007: Summarizing responses to a series of case-management scenarios

Scenario and responses<br>[question/variable in dataset]	No. and percent of health workers (N=93)	
	n	%
**Scenario 5.** "A 22-year old man comes to see you with 2 days of headache, vomiting, fatigue, and joint pain. He states he does not have fever, and he has no other symptoms. Although he is tired, he does not appear critically ill. His temperature is normal (36.5ºC), and vital signs are normal." The correct response is that either microscopy or an RDT should be ordered. [E20, E20_cat]		
Response considered correct: Microscopy ordered (n=46), or RDT ordered (n=26), or microscopy + RDT ordered (n=9)	81	87.1%
Neither microscopy nor RDT ordered (n=12)	12	12.9%
**Scenario 6.** "A 4-year old child comes to a consultation with fever." The correct response according to the old policy is that either microscopy or an RDT should be ordered; the correct response according to the new policy is that neither microscopy nor an RDT should be ordered. [E21, E21_cat]		
Response considered correct (for the old policy): Microscopy ordered (n=35), or RDT ordered (n=37), or microscopy + RDT ordered (n=8)	80	86.0%
Response considered correct (for the new policy): Neither microscopy nor RDT ordered (n=13)	13	14.0%
**Scenario 7.** "A 4-year old child comes to a consultation with diarrhea, chills, fatigue, and poor appetite. The mother states that the child did not have fever, and the temperature is normal (36.4ºC)." The correct response is that either microscopy or an RDT should be ordered. [E22, E22_cat]		
Response considered correct: Microscopy ordered (n=36), or RDT ordered (n=32), or microscopy + RDT ordered (n=3)	71	76.3%
Neither microscopy nor RDT ordered (n=22)	22	23.7%

Footnotes for Table 12.

RDT = rapid diagnostic test.

[a] This response was considered correct because surveyors did not prompt health workers to specify illness severity.

Table 13. Demographic and care-seeking characteristics of patients seen for an initial consultation in outpatient health facilities, Huambo Province, Angola, October–November 2007

Characteristic [question/variable in dataset]	No. and weighted percentage of patients		
	n	%	(95% CI)
Patient age (among all 177 patients) [AGE_YRS, B1a, B1b, B1c]			
0–4 years[a]	72	45.0%	(29.6–60.4)
5–14 years	20	11.9%	(4.7–19.1)
15–49 years	71	37.0%	(22.8–51.2)
50 years and older	14	6.1%	(1.5–10.7)
Female sex (among all 177 patients) [A4a]	99	55.9%	(43.3–68.5)
Time between illness onset and the consultation (among all 177 patients) [ONSET, C2, D2]			
0 days (illness began today)	13	7.0%	(2.2–11.9)
1 day	47	23.8%	(14.5–33.0)
2 days	38	22.4%	(43.3–68.5)
More than 2 days	78	46.5%	(35.6–57.3)
Not sure	1	0.3%	(0–1.0)
Time between illness onset and the consultation (among 72 patients <5 years old) [ONSET, C2]			
0 days (illness began today)	5	9.8%	(0.9–18.6)
1 day	23	23.2%	(7.2–39.2)
2 days	16	24.2%	(15.9–32.5)
More than 2 days	28	42.9%	(27.9–57.8)

Footnotes for Table 13.

CI = confidence interval.

[a] Weighted percentages (among all 177 patients) for individual years were as follows: 19.4% (n=30) were <12 months old, 8.5% (n=16) were 1 year old, 13.3% (n=17) were 2 years old, 3.3% (n=6) were 3 years old, and 0.5% (n=3) were 4 years old.

Table 14. Percentages of patients seen at health facilities or seen by health workers with different characteristics, Huambo Province, Angola, October–November 2007

Characteristic [question/variable in dataset]	No. and weighted percentage of patients (N=177)	
	n	% (95% CI)
Patient seen in a health facility with the following drugs in stock[a]		
AL blister packs for patients 5–14 kg [F13a1]	141	70.8% (49.3–92.3)
AL blister packs for patients 15–24 kg [F13b1]	177	100% (NC)
AL blister packs for patients 25–34 kg [F13c1]	177	100% (NC)
AL blister packs for patients ≥35 kg [F13d1]	163	89.8% (77.4–100)
Amodiaquine tablets [F13g1]	156	94.1% (87.1–100)
Quinine (tablets) [F13l1]	71	32.4% (10.5–54.2)
Quinine or quinidine (injectable) [F13m1]	99	64.5% (45.0–84.0)
Oral antibiotic [F13n1]	172	91.7% (78.9–100)
Iron [F13o1]	149	68.2% (43.9–92.5)
Oral rehydration solution [F13p1]	126	47.4% (25.3–69.4)
Patient seen in a health facility with the ability to perform malaria testing (microscopy or rapid diagnostic test) [DX_AVAIL]	177	100% (NC)
Patient seen by a health worker with the following characteristics[b]		
Received formal training on ACTs [E8a]	94	53.9% (33.5–74.3)
Received any training on ACTs (formal or informal) [E8a, E8f]	134	75.3% (56.7–93.8)

Footnotes for Table 14.

ACT = artemisinin-based combination therapy; AL = artemether-lumefantrine; CI = confidence interval; NC = not calculated.

[a] The following commodities were not analyzed because no health facility had them in stock: 1) bednets, 2) artesunate tablets, 3) injectable artemisinins, and 4) artemisinin suppositories.

[b] Analysis includes 171 patients (6 patients excluded because interviews were not completed for the health worker who performed the consultation).

Table 15. Chief complaints observed during consultations of patients seen at outpatient health facilities, Huambo Province, Angola, October–November 2007

Characteristic [question/variable in dataset]	No. and weighted percentage[a] of patients	
	n	% (95% CI)
Chief complaints among all 177 patients		
Fever or malaria [A7a]	84	49.0% (40.4–57.6)
Diarrhea or vomiting [A7b]	31	18.7% (11.8–25.5)
Respiratory problem, cough, or flu-like illness [A7c]	37	25.1% (8.7–41.4)
Ear problem [A7d]	5	4.1% (0–8.3)
Any other complaint[b] [CC_OTHER_OBS]	133	70.5% (57.3–83.2)
Chief complaints among children <5 years old (N=72) [B2]		
Fever or malaria [A7a]	48	63.6% (53.5–73.7)
Diarrhea or vomiting [A7b]	27	35.0% (24.1–45.8)
Respiratory problem, cough, or flu-like illness [A7c]	20	38.2% (9.2–67.1)
Ear problem [A7d]	1	2.6% (0–7.8)
Any other complaint [CC_OTHER_OBS]	33	41.0% (29.2–52.7)
Chief complaints among patients ≥5 years old (N=105) [B2]		
Fever or malaria [A7a]	36	37.0% (25.9–48.2)
Diarrhea or vomiting [A7b]	4	5.3% (0–11.1)
Respiratory problem, cough, or flu-like illness [A7c]	17	14.3% (4.6–24.1)
Ear problem [A7d]	4	5.4% (0–11.9)
Any other complaint [CC_OTHER_OBS]	100	94.7% (85.9–100)

Footnotes for Table 15.

CI = confidence interval.

[a] Percentages do not sum to 100% because patients could have had more than one chief complaint.

[b] The most common other chief complaints were headache, stomach ache, and chest pain.

Table 16. Chief complaints based on survey exit interviews of patients seen at outpatient health facilities, Huambo Province, Angola, October–November 2007

Characteristic [question/variable in dataset]	No. and weighted percentage[a] of patients	
	n	% (95% CI)
Chief complaints among all 177 patients		
Fever or malaria [CC_FEVR]	87	49.7% (41.5–57.9)
Diarrhea or vomiting [CC_DIAR]	32	19.3% (11.7–26.9)
Respiratory problem, cough, or flu-like illness [CC_CDBR]	39	26.8% (11.7–42.0)
Ear problem [CC_EARS]	3	1.4% (0–3.2)
Any other complaint[b] [CC_OTHER]	131	74.1% (65.6–82.6)
Chief complaints among children <5 years old (N=72) [B2]		
Fever or malaria [C1a]	46	58.6% (45.2–72.0)
Diarrhea or vomiting [C1b]	26	33.4% (22.2–44.5)
Respiratory problem, cough, or flu-like illness [C1c]	21	38.6% (10.0–67.2)
Ear problem [C1d]	72	0% (NC)
Any other complaint [CC_OTHER]	33	50.8% (33.8–67.9)
Chief complaints among patients ≥5 years old (N=105) [B2]		
Fever or malaria [D1a]	41	42.4% (33.6–51.3)
Diarrhea or vomiting [D1b]	6	7.8% (1.5–14.2)
Respiratory problem, cough, or flu-like illness [D1c]	18	17.2% (6.7–27.7)
Ear problem [D1d]	3	2.6% (0–5.7)
Any other complaint [CC_OTHER]	98	93.1% (84.0–100)

Footnotes for Table 16.

CI = confidence interval; NC = not calculated.

[a] Percentages do not sum to 100% because patients could have had more than one chief complaint.

[b] The most common other chief complaints were headache, stomach ache, and chest pain.

Table 17. The agreement of chief complaints observed during patient consultations and chief complaints based on survey exit interviews for all 177 patients[a] seen at outpatient health facilities, Huambo Province, Angola, October–November 2007

Characteristic [question/variable in dataset]	No. and unweighted percentage of patients n (%)
Chief complaint of fever or malaria [A7a, CC_FEVR]	
Observed and exit interview results agree	160 (90.4%)
Complaint found only during observation of consultations	7 (4.0%)
Complaint found only during exit interview	10 (5.7%)
Chief complaint of diarrhea or vomiting [A7b, CC_DIAR]	
Observed and exit interview results agree	174 (98.3%)
Complaint found only during observation of consultations	1 (0.6%)
Complaint found only during exit interview	2 (1.1%)
Chief complaint of respiratory problem, cough, or flu-like illness [A7c, CC_CDBR]	
Observed and exit interview results agree	165 (93.2%)
Complaint found only during observation of consultations	5 (2.8%)
Complaint found only during exit interview	7 (4.0%)

Footnotes for Table 17.

[a] Results were very similar for subgroups of patients <5 years old and ≥5 years old.

Note that there were too few patients who complained of an ear problem (only 5) for a meaningful analysis.

Table 18. Suspected malaria and non-malaria causes of febrile illness of patients seen for an initial consultation in outpatient health facilities, Huambo Province, Angola, October–November 2007

Illness [question/variable in dataset]	No. and weighted percentage of patients	
	n	% (95% CI)
Patient had suspected malaria[a] [SUSMAL]		
Among all 177 patients	136	77.8% (69.5–86.2)
Among the 72 patients <5 years old	62	79.5% (65.3–93.7)
Among the 105 patients ≥5 years old	74	76.5% (66.1–86.9)
Patient had another cause of febrile illness[b] (among the 136 patients of all ages with suspected malaria)		
Influenza-like illness [FLU_LIKE]	42	31.2% (20.1–42.3)
Pneumonia [PNEUMONIA]	15	13.0% (6.1–19.9)
Otitis media [OTITS]	10	10.2% (1.3–19.0)
Measles [MEASLES]	0	0% (NC)
Dysentery [DYSENTERY]	6	3.8% (0–8.0)
Urinary tract infection [UTI]	5	4.9% (0–12.5)
Hepatitis [HEPATITIS]	3	2.6% (0–5.8)
At least one of the above other causes [OTHERCAUSE]	63	48.0% (36.8–59.3)
Combinations of illnesses (among the 63 patients with suspected malaria and another cause of febrile illness)		
Influenza-like illness only	28	34.8%
Influenza-like illness and pneumonia	7	14.0%
Influenza-like illness and otitis media	4	11.5%
Urinary tract infection only	4	9.6%
Pneumonia only	5	7.1%
Otitis media only	4	5.9%
Dysentery only	4	4.6%
Pneumonia and otitis media	1	3.1%
Hepatitis only	1	2.3%
Influenza-like illness and hepatitis	1	2.1%
Pneumonia and dysentery	1	1.9%
Influenza-like illness and dysentery	1	1.5%
Influenza-like illness, pneumonia, and hepatitis	1	1.0%
Otitis media and urinary tract infection	1	0.6%

Table 18 continued on next page.

Table 18, continued. Suspected malaria and non-malaria causes of febrile illness of patients seen for an initial consultation in outpatient health facilities, Huambo Province, Angola, October–November 2007

Illness [question/variable in dataset]	No. and weighted percentage of patients	
	n	% (95% CI)
Patient had another cause of febrile illness (among 62 patients <5 years old with suspected malaria)		
Influenza-like illness [FLU_LIKE]	25	47.4% (34.8–60.0)
Pneumonia [PNEUMONIA]	9	18.3% (6.8–29.8)
Otitis media [OTITS]	4	12.0% (0–26.4)
Measles [MEASLES]	0	0% (NC)
Dysentery [DYSENTERY]	6	8.3% (0–16.7)
Urinary tract infection [UTI]	0	0% (NC)
Hepatitis [HEPATITIS]	0	0% (NC)
At least one of the above other causes [OTHERCAUSE]	31	55.8% (41.9–69.8)
Combinations of illnesses (among 31 patients <5 years old with suspected malaria and another cause of febrile illness)		
Influenza-like illness only	13	34.3%
Influenza-like illness and pneumonia	7	26.1%
Influenza-like illness and otitis media	4	21.6%
Dysentery only	4	8.5%
Pneumonia and dysentery	1	3.5%
Pneumonia only	1	3.1%
Influenza-like illness and dysentery	1	2.9%

Table 18 continued on next page.

Table 18, continued. Suspected malaria and non-malaria causes of febrile illness of patients seen for an initial consultation in outpatient health facilities, Huambo Province, Angola, October–November 2007

Illness [question/variable in dataset]	No. and weighted percentage of patients	
	n	% (95% CI)
Patient had another cause of febrile illness (among 74 patients ≥5 years old with suspected malaria)		
Influenza-like illness [FLU_LIKE]	17	17.5% (5.3–29.6)
Pneumonia [PNEUMONIA]	6	8.5% (1.5–15.6)
Otitis media [OTITS]	6	8.5% (0.9–16.2)
Measles [MEASLES]	0	0% (NC)
Dysentery [DYSENTERY]	0	0% (NC)
Urinary tract infection [UTI]	5	9.1% (0–22.2)
Hepatitis [HEPATITIS]	3	4.8% (0–10.9)
At least one of the above other causes [OTHERCAUSE]	32	41.4% (25.5–57.4)
Combinations of illnesses (among 32 patients ≥5 years old with suspected malaria and another cause of febrile illness)		
Influenza-like illness only	15	35.5%
Urinary tract infection only	4	20.6%
Otitis media only	4	12.6%
Pneumonia only	4	11.7%
Pneumonia and otitis media	1	6.7%
Hepatitis only	1	4.9%
Influenza-like illness and hepatitis	1	4.4%
Influenza-like illness, pneumonia, and hepatitis	1	2.2%
Otitis media and urinary tract infection	1	1.4%

Footnotes for Table 18.

CI = confidence interval; NC = not calculated.

[a] Defined as either fever (history of fever or axillary temperature >37.5ºC), or at least 3 of the following: headache, joint pain, chills, sweating, anemia (palmor pallor), cough (applies to children only), anorexia, fatigue, vomiting, or diarrhea.

[b] See Box 2 for case definitions.

Table 19. Prevalence of malaria cases according to the "old" (pre-September 2007) policy[a] among patients seen for an initial consultation in outpatient health facilities, Huambo Province, Angola, October–November 2007

Illness [question/variable in dataset]	No. and weighted percentage of patients	
	n	% (95% CI)
Malaria cases among all 177 patients [OUR_DX_OLD]		
Complicated malaria	1	0.8% (0–2.4)
Uncomplicated malaria	58	35.0% (26.0–44.1)
Not malaria	118	64.2% (55.1–73.3)
Malaria cases among 72 patients <5 years old [OUR_DX_OLD]		
Complicated malaria	0	0% (NC)
Uncomplicated malaria	23	30.5% (20.4–40.7)
Not malaria	49	69.5% (59.3–79.6)
Malaria cases among 105 patients ≥5 years old [OUR_DX_OLD]		
Complicated malaria	1	1.4% (0–4.3)
Uncomplicated malaria	35	38.7% (26.1–51.4)
Not malaria	69	59.9% (47.7–72.0)

Footnotes for Table 19.

CI = confidence interval; NC = not calculated.

[a] Malaria defined by applying an analysis algorithm (Figure 3), which reflects the pre-September 2007 malaria case-management policy as implemented in Huambo, to patient clinical signs and symptoms (assessed by surveyors, but information that should have been available to the observed health workers) and laboratory data available to the observed health workers (i.e., laboratory results of tested ordered by the observed health workers—not the "gold standard" survey team's laboratory results).

Table 20. Prevalence of malaria cases according to the "new" policy[a] (announced in September 2007) among patients seen for an initial consultation in outpatient health facilities, Huambo Province, Angola, October–November 2007

Illness [question/variable in dataset]	No. and weighted percentage of patients	
	n	% (95% CI)
Malaria cases among all 177 patients [OUR_DX_NEW]		
Complicated malaria	4	4.3% (0–9.0)
Uncomplicated malaria	74	45.9% (36.9–55.0)
Not malaria	99	49.7% (42.5–57.0)
Malaria cases among 72 patients <5 years old [OUR_DX_ NEW]		
Complicated malaria	3	7.9% (0–16.5)
Uncomplicated malaria	39	54.8% (38.5–71.0)
Not malaria	30	37.3% (26.6–48.1)
Malaria cases among 105 patients ≥5 years old [OUR_DX_ NEW]		
Complicated malaria	1	1.4% (0–4.3)
Uncomplicated malaria	35	38.7% (26.1–51.4)
Not malaria	69	59.9% (47.7–72.0)

Footnotes for Table 20.

CI = confidence interval.

[a] Malaria defined by applying an analysis algorithm (Figure 4), which reflects the national malaria case-management policy that was announced in September 2007, to patient clinical signs and symptoms (assessed by surveyors, but information that should have been available to the observed health workers) and laboratory data available to the observed health workers (i.e., laboratory results of tested ordered by the observed health workers—not the "gold standard" survey team's laboratory results).

Table 21. Quality of assessment of patients seen for an initial consultation in outpatient health facilities, Huambo Province, Angola, October–November 2007

Assessment task [question/variable in dataset]	No. and weighted percentage of patients								
	All patients (N=177)			Age <5 years (N=72)			Age ≥5 years (N=105)		
	n	%	(95% CI)	n	%	(95% CI)	n	%	(95% CI)
Determine[a] history of fever [DET_FEV]	152	87.6%	(80.7–94.6)	68	94.8%	(88.9–100)	84	81.8%	(70.2–93.4)
Determine headache [DET_HA]	76	39.2%	(29.1–49.2)	5	9.8%	(1.1–18.5)	71	63.2%	(51.6–74.8)
Determine joint pain [DET_JP]	52	23.1%	(14.0–32.2)	0	0%	(NC)	52	42.0%	(28.8–55.3)
Determine vomiting [DET_VOM]	47	24.1%	(13.8–34.4)	30	34.0%	(14.2–53.8)	17	16.0%	(7.1–24.9)
Determine chills [DET_CHILLS]	5	3.7%	(0.3–7.1)	2	2.2%	(0–5.5)	3	4.9%	(0–10.6)
Determine poor appetite [DET_ANOREX]	56	26.8%	(17.5–36.2)	30	35.1%	(23.8–46.3)	26	20.1%	(8.9–31.3)
Determine fatigue [DET_FATIG]	5	1.8%	(0–3.8)	1	0.5%	(0–1.7)	4	2.7%	(0–6.2)
Determine diarrhea [DET_DIAR]	61	37.5%	(28.3–46.7)	42	59.6%	(52.6–66.6)	19	19.4%	(8.2–30.7)
Determine cough (for children only) [DET_COUGH]	Not applicable			51	77.5%	(62.9–92.0)	Not applicable		
Measure patient's temperature [A9a]	55	25.9%	(14.1–37.8)	34	37.8%	(23.2–52.4)	21	16.2%	(3.5–28.9)
Check for anemia[b] [DET_ANEMIA]	23	16.2%	(6.8–25.7)	12	19.0%	(3.4–34.6)	11	14.0%	(2.7–25.3)

Footnotes for Table 21.

CI = confidence interval; NC = not calculated.

[a] Health worker determined whether the patient had the symptom by asking the patient or by having the information obviously available (e.g., the patient spontaneously offered the information during the consultation).

[b] Health worker examined either the inside of the patient's mouth, palms, or fingertips.

Table 22. Quality of assessment of non-fever symptoms among patients without fever[a] seen for an initial consultation in outpatient health facilities, Huambo Province, Angola, October–November 2007

Assessment task [question/variable in dataset]	No. and weighted percentage of patients					
	All patients (N=58)		Age <5 years (N=14)		Age ≥5 years (N=44)	
	n	% (95% CI)	n	% (95% CI)	n	% (95% CI)
Determine[b] headache [DET_HA]	30	41.6% (19.1–64.0)	0	0% (NC)	30	68.6% (48.5–88.8)
Determine joint pain [DET_JP]	17	26.0% (10.2–41.8)	0	0% (NC)	17	42.9% (26.5–59.4)
Determine vomiting [DET_VOM]	14	33.1% (11.5–54.7)	6	33.2% (0–73.2)	8	33.0% (11.6–54.4)
Determine chills [DET_CHILLS]	0	0% (NC)	0	0% (NC)	0	0% (NC)
Determine poor appetite [DET_ANOREX]	14	28.2% (12.6–43.8)	7	43.8% (23.0–64.6)	7	18.0% (0.3–35.8)
Determine fatigue [DET_FATIG]	1	0.9% (0–2.7)	0	0% (NC)	1	1.4% (0–4.4)
Determine diarrhea [DET_DIAR]	19	44.6% (26.2–63.0)	10	65.3% (42.3–88.3)	9	31.1% (8.9–53.4)
Determine cough (for children only) [DET_COUGH]	Not applicable		11	84.5% (61.8–100)	Not applicable	
Check for anemia[c] [DET_ANEMIA]	5	9.2% (0–20.6)	0	0% (NC)	5	15.3% (0–32.7)

<u>Footnotes for Table 22.</u>

CI = confidence interval; NC = not calculated.

[a] History of fever or measured temperature >37.5 ºC (by survey team).

[b] Health worker determined whether the patient had the symptom by asking the patient or by having the information obviously available (e.g., the patient spontaneously offered the information during the consultation).

[c] Health worker examined either the inside of the patient’s mouth, palms, or fingertips.

Table 23. Appropriateness of the use of malaria diagnostic testing ordered by observed health workers among patients seen for an initial consultation in outpatient health facilities, Huambo Province, Angola, October–November 2007

Indicator [question/variable in dataset]	No. and weighted percentage of patients								
	All patients			Age <5 years			Age ≥5 years		
	n/N	%	(95% CI)	n/N	%	(95% CI)	n/N	%	(95% CI)
Analysis according to the old policy [NEEDTEST_OLD, GOT_TEST, COR_TEST_OLD, B2]									
Proportion of patients who needed testing (N) who were tested (n)	58/136	30.7%	(17.9–43.6)	28/62	30.0%	(11.9–46.7)	30/74	32.0%	(13.6–50.3)
Proportion of patients who did not need testing (N) who were not tested (n)	35/41	79.2%	(60.5–97.9)	8/10	81.6%	(NC)	27/31	77.4%	(53.6–100)
Overall adherence to the old policy[a] [COR_TEST_OLD]	93/177	41.5%	(30.2–52.7)	36/72	40.0%	(25.0–55.0)	57/105	42.7%	(27.7–57.6)
Analysis according to the new policy [NEEDTEST_NEW, GOT_TEST, COR_TEST_NEW, B2]									
Proportion of patients who needed testing (N) who were tested (n)	30/74	32.0%	(13.6–50.3)	NC (no testing needed for children <5 years old)			30/74	32.0%	(13.6–50.3)
Proportion of patients who did not need testing (N) who were not tested (n)	69/103	73.9%	(60.7–87.2)	42/72	72.9%	(56.4–89.5)	27/31	77.4%	(53.6–100)
Overall adherence to the new policy[a] [COR_TEST_NEW]	99/177	56.3%	(42.3–70.2)	42/72	72.9%	(56.4–89.5)	57/105	42.7%	(27.7–57.6)

Footnotes for Table 23.

CI = confidence interval; NC = not calculated.

[a] Patients needing testing got tested, and patients not needing testing did not get tested.

Table 24. Predictors of correct diagnostics use, according to the "old" (pre-September 2007) policy among patients with suspected malaria (who all needed testing by either microscopy or a rapid diagnostic test) seen for an initial consultation in outpatient health facilities, Huambo Province, Angola, October–November 2007—univariate results

Attribute [question/variable in dataset]	No. of patients tested / no. of patients who needed testing (weighted %)	Univariate odds ratio (95% CI)	p-value	R-squared
All patients who needed testing [NEEDTEST_OLD]	58/136 (30.7%)	NA	NA	NA
HW attended at least one formal training on AL (N=131; 5 missing) [TR_FORMAL]				
Yes	30/66 (36.5%)	1.46 (0.47–4.59)	0.51	5.8%
No	27/65 (28.2%)	reference		
Type of HW training on AL (analyzed with 4 levels) (N=131; 5 missing) [TR_4LEVEL]				
Formal and informal training	25/56 (36.4%)	Not analyzed[a]		
Formal training only	5/10 (36.8%)			
Informal training only	18/33 (41.4%)			
No AL training	9/32 (17.2%)			
Type of HW training on AL (analyzed with 2 levels) (N=131; 5 missing) [TR_ANY]				
Any AL training (formal or informal training)	48/99 (38.1%)	2.96 (0.78–11.21)	0.11	27.8%
No AL training	9/32 (17.2%)	reference		
Number of days of AL training[b] (N=131; 5 missing) [TR_DAYS, TR_DAYS_CAT]				
More than 6 days	5/16 (23.6%)	odds ratio per additional day of training: 0.97 (0.91–1.04)	0.43	4.4%
4–6 days	13/24 (38.8%)			
1–3.5 days	12/26 (43.9%)			
0.5 days (informal training only)	18/33 (41.4%)			
0 days (no AL training)	9/32 (17.2%)			

Table 24 continued on next page.

Table 24, continued. Predictors of correct diagnostics use, according to the "old" (pre-September 2007) policy among patients with suspected malaria (who all needed testing by either microscopy or a rapid diagnostic test) seen for an initial consultation in outpatient health facilities, Huambo Province, Angola, October–November 2007—univariate results

Attribute [question/variable in dataset]	No. of patients tested / no. of patients who needed testing (weighted %)	Univariate odds ratio (95% CI)	p-value	R-squared
HW supervised on AL use in past 6 months (N=131, 5 missing) [E10a]				
Supervised 3 times	5/7 (71.2%)	odds ratio per additional supervision visit: 1.84 (1.01–3.36)	0.046	33.8%
Supervised 2 times	18/32 (55.0%)			
Supervised 1 time	11/33 (30.2%)			
Not supervised	23/59 (25.0%)			
HW's caseload on day of survey visit (N=130, 6 missing) [CLOAD_CAT, E12]				
40–43 patients	0/1 (0%)	odds ratio per additional patient: 0.93 (0.88–0.97)	0.002	60.7%
35–39 patients	1/7 (13.0%)			
30–34 patients	2/11 (8.7%)			
25–29 patients	0/8 (0%)			
20–24 patients	8/13 (63.7%)			
15–19 patients	12/26 (45.3%)			
10–14 patients	20/37 (57.9%)			
5–9 patients	7/19 (25.1%)			
0–4 patients	6/8 (57.0%)			
HW's caseload on day of survey visit (N=130, 6 missing) [CLOAD_024]				
0–24 patients	53/103 (49.0%)	11.88 (2.88–49.01)	0.0006	81.0%
25–43 patients	3/27 (7.5%)	reference		

Table 24 continued on next page.

Table 24, continued. Predictors of correct diagnostics use, according to the “old” (pre-September 2007) policy among patients with suspected malaria (who all needed testing by either microscopy or a rapid diagnostic test) seen for an initial consultation in outpatient health facilities, Huambo Province, Angola, October–November 2007—univariate results

Attribute [question/variable in dataset]	No. of patients tested / no. of patients who needed testing (weighted %)	Univariate odds ratio (95% CI)	p-value	R-squared
HW knowledge score on three case-management scenarios related to selecting which patients needed malaria testing (N=131, 5 missing) [KNOW_TEST]				
All 3 scenarios answered correctly	37/85 (31.2%)	odds ratio per additional correct answer: 0.94 (0.62–1.44)	0.78	0.4%
2 scenarios answered correctly	17/36 (36.3%)			
1 scenario answered correctly	0/3 (0%)			
0 scenarios answered correctly	3/7 (43.0%)			
HW’s sex (N=131, 5 missing) [SEX_HW]				
Male	39/76 (41.5%)	2.11 (0.76–5.81)	0.151	20.1%
Female	18/55 (25.2%)	reference		
HW’s age (N=131, 5 missing) [HW_AGE_CAT, E3m1]				
50–70 years	6/15 (22.9%)	odds ratio per additional year of age: 0.96 (0.91–1.01)	0.098	25.9%
40–49 years	16/41 (25.9%)			
30–39 years	19/37 (33.7%)			
20–29 years	16/38 (43.0%)			

Table 24 continued on next page.

Table 24, continued. Predictors of correct diagnostics use, according to the "old" (pre-September 2007) policy among patients with suspected malaria (who all needed testing by either microscopy or a rapid diagnostic test) seen for an initial consultation in outpatient health facilities, Huambo Province, Angola, October–November 2007—univariate results

Attribute [question/variable in dataset]	No. of patients tested / no. of patients who needed testing (weighted %)	Univariate odds ratio (95% CI)	p-value	R-squared
Chief complaint of patient (or patient's caretaker) was fever or malaria (N=136) [A7a]				
Yes	39/84 (29.6%)	0.86 (0.41–1.83)	0.70	0.9%
No	19/52 (32.8%)	reference		
Patient's temperature measured by surveyor (N=135; 1 missing) [TEMPm1, TEMP_INT]				
39.0–39.9ºC	9/11 (80.5%)	odds ratio per additional 1ºC increase: 2.02 (1.30–3.13)	0.002	51.0%
38.0–38.9ºC	3/9 (29.9%)			
37.0–37.9ºC	19/37 (30.2%)			
36.0–36.9ºC	25/70 (26.2%)			
35.0–35.9ºC	1/8 (6.0%)			
Patient's sex (N=136) [SEX_PT]				
Male	22/59 (29.2%)	0.87 (0.29–2.61)	0.80	0.8%
Female	36/77 (32.2%)	reference		
Patient's age (N=136) [PT_AGE_CAT, AGE_YRS]				
50–75 years	1/9 (13.8%)	odds ratio per additional year increase: 0.99 (0.97–1.02)	0.53	3.1%
40–49 years	5/14 (17.1%)			
30–39 years	7/12 (55.6%)			
20–29 years	5/12 (22.1%)			
10–19 years	10/19 (41.4%)			
5–9 years	2/8 (32.7%)			
2–4 years	7/23 (14.5%)			
0–1 years	21/39 (40.1%)			

Table 24 continued on next page.

Table 24, continued. Predictors of correct diagnostics use, according to the "old" (pre-September 2007) policy among patients with suspected malaria (who all needed testing by either microscopy or a rapid diagnostic test) seen for an initial consultation in outpatient health facilities, Huambo Province, Angola, October–November 2007—univariate results

Attribute [question/variable in dataset]	No. of patients tested / no. of patients who needed testing (weighted %)	Univariate odds ratio (95% CI)	p-value	R-squared
Health facility type (N=136) [HF_TYPE, HC_ADEQ, HC_LOW]				
Health center with "low" infrastructure[c]	36/67 (50.1%)	5.71 (1.03–31.79)	0.047	44.0%
Health center with "adequate" infrastructure[c]	18/51 (27.6%)	2.17 (0.33–14.24)	0.42	
Hospital	4/18 (15.0%)	reference		
Group of municipalities (N=136) [MENTOR]				
Initial five municipalities (Huambo, Bailundo, Caála, Londuimbali, and Tchikala Tcholohanga)	45/99 (29.8%)	0.81 (0.22–2.96)	0.75	1.2%
Other six municipalities	13/37 (34.4%)	reference		

Footnotes for Table 24.

AL = artemether-lumefantrine; CI = confidence interval; HW = health worker.

[a] Results for the three groups of patients seen by trained HWs were similar, so they were combined into one category (see results for "TR_ANY" on the next row of the table).

[b] See methods for how days of training was estimated.

[c] Health centers with adequate infrastructure are larger outpatient health facilities with a laboratory and inpatient ward, and where antenatal consultations are performed. Health centers with low infrastructure are larger outpatient health facilities that lack at least one of the following: a laboratory, an inpatient ward, or antenatal consultations.

Table 25. Predictors of correct diagnostics use, according to the "old" (pre-September 2007) policy among patients with suspected malaria (who all needed testing by either microscopy or a rapid diagnostic test) seen for an initial consultation in outpatient health facilities, Huambo Province, Angola, October–November 2007—multivariate results

Attribute [question/variable in dataset]	Adjusted odds ratio[b] (95% CI)	p-value
Full model[a] with all variables with univariate p-values < 0.15		
HW's caseload on day of survey visit (0–24 vs. 25–43 patients) [CLOAD_024]	17.68 (5.83–53.60)	<0.0001
Patient's temperature measured by surveyor (mean centered) [TEMPm1]	2.42[c] (1.16–5.06)	0.018
Type of HW training on AL (any training vs. no training) [TR_ANY]	4.82 (0.80–29.00)	0.086
Health facility type [HC_ADEQ, HC_LOW]		
Health center with "low" infrastructure[d]	2.26 (0.80–6.39)	0.12
Health center with "adequate" infrastructure[d]	1.78 (0.57–5.59)	0.32
Hospital	reference	
HW supervised on AL use in past 6 months [E10a]	1.10[e] (0.59–2.04)	0.77
HW's age (mean centered) [E3m1]	1.00[f] (0.95–1.05)	0.99
Reduced model[a] with only variables with multivariate p-values < 0.10		
HW's caseload on day of survey visit (0–24 vs. 25–43 patients) [CLOAD_024]	18.35 (6.79–49.57)	<0.0001
Patient's temperature measured by surveyor (mean centered) [TEMPm1]	2.53[c] (1.28–5.00)	0.0073
Type of HW training on AL (any training vs. no training) [TR_ANY]	5.36 (0.86–33.47)	0.072

Footnotes for Table 25.

AL = artemether-lumefantrine; CI = confidence interval; HW = health worker.

[a] For both the full and reduced models, the dichotomous outcome was GOT_TEST, and the analysis was based on 129 patients (7 missing patients because of missing values of predictor variables). For the full model, the r-squared was 94.9%, the condition index was 14.3 (indicating no collinearity), and the model was: GOT_TEST = –4.8387 + (2.8726 x CLOAD_024) + (0.8847 x TEMPm1) + (1.5717 x TR_ANY) + (0.0928 x E10a) + (–0.00037 x E3m1) + (0.5773 x HC_ADEQ) + (0.8158 x HC_LOW). For the reduced model, the results were virtually identical to the full model; the r-squared was 94.4%, the condition index was 10.0 (indicating no collinearity), and the model was: GOT_TEST = –4.3062 + (2.9097 x CLOAD_024) + (0.9294 x TEMPm1) + (1.6793 x TR_ANY).

[b] Adjusted for all other variables in the model.

[c] Odds ratio per additional 1ºC increase in patient's temperature; variable centered with a mean of 37.045ºC.

[d] For definitions, see footnotes of Table 1.

[e] Odds ratio per additional supervision visit.

[f] Odds ratio per additional year of age; variable centered with a mean of 36.542 years.

Table 26. Details and results of malaria testing ordered by observed health workers in outpatient health facilities, Huambo Province, Angola, October–November 2007

Characteristic and patient sub-group [question/variable in dataset]	No. and weighted percentage of patients	
	n	% (95% CI)
Testing results (microscopy and RDT combined) among all 177 patients [TESTRES]		
Patient not tested	113	71.5% (60.8–82.1)
Patient tested negative	45	20.7% (12.8–28.7)
Patient tested positive	17	6.7% (2.0–11.3)
Patient tested, but results not available that day	2	1.1% (0–2.8)
Testing results (detailed) among all 177 patients [TESTRES2]		
Patient not tested	113	71.5% (60.8–82.1)
No smear and RDT-negative	37	14.8% (7.2–22.3)
No smear and RDT-positive	13	4.2% (1.0–7.3)
Smear-positive and RDT-positive	1	0.8% (0–2.4)
Smear-negative and RDT- negative	2	1.5% (0–3.8)
Smear-positive and no RDT	3	1.7% (0–4.0)
Smear-negative and no RDT	6	4.4% (0.5–8.4)
Smear results not available that day and no RDT	2	1.1% (0–2.8)
Testing results (microscopy and RDT combined) among the 64 tested patients [TESTRES]		
Patient tested negative	45	72.7% (60.5–84.8)
Patient tested positive	17	23.4% (11.2–35.7)
Patient tested, but results not available that day	2	3.9% (0–9.7)
Testing results (detailed) among the 64 tested patients [TESTRES2]		
No smear and RDT-negative	37	51.7% (33.7–69.6)
No smear and RDT-positive	13	14.6% (5.3–24.0)
Smear-positive and RDT-positive	1	2.7% (0–8.2)
Smear-negative and RDT- negative	2	5.4% (0–12.9)
Smear-positive and no RDT	3	6.2% (0–13.2)
Smear-negative and no RDT	6	15.6% (1.4–29.7)
Smear results not available that day and no RDT	2	3.9% (0–9.7)

Footnotes for Table 26.

CI = Confidence interval; NC = not calculated; RDT = rapid diagnostic test.

Table 27. Quality of malaria diagnosis according to the "old" (pre-September 2007) policy in outpatient health facilities, Huambo Province, Angola, October–November 2007

Characteristic and patient sub-group [question/variable in dataset]	No. and weighted percentage of patients	
	n	% (95% CI)
Health worker's malaria-related diagnosis for the 1 patient with a "gold standard" diagnosis[a] of <u>complicated malaria</u> [HW_DX_MALARIA2]		
Uncomplicated malaria (major error)	1	100% (NC)
Health workers' malaria-related diagnoses for the 58 patients with a "gold standard" diagnosis[a] of <u>uncomplicated malaria</u> [HW_DX_MALARIA2]		
Complicated malaria (minor error)	2	2.6% (0–6.4)
Uncomplicated malaria (correct)	32	60.1% (44.5–75.7)
No malaria[b] (major error)	24	37.4% (21.6–53.1)
Health workers' malaria-related diagnoses for the 118 patients with a "gold standard" diagnosis[a] of <u>no malaria</u> [HW_DX_MALARIA2]		
Uncomplicated malaria (minor error)	40	29.9% (17.6–42.2)
No malaria (correct)	78	70.1% (57.8–82.4)
Overall quality of health workers' malaria-related diagnoses among all 177 patients [HW_MAL_DX_QUAL_OLD]		
Correct (health worker diagnoses of malaria and no malaria matched "gold standard" diagnoses)	110	66.1% (58.3–73.8)
Minor error (health worker incorrectly "over-diagnosed" uncomplicated malaria as complicated malaria, or over-diagnosed no malaria as uncomplicated malaria)	42	20.1% (11.4–28.7)
Major error (health worker incorrectly "under-diagnosed" complicated malaria as uncomplicated malaria, or under-diagnosed uncomplicated malaria as no malaria)	25	13.9% (8.0–19.7)

<u>Footnotes for Table 27.</u>

CI = confidence interval; NC = not calculated.

[a] The "gold standard" malaria diagnosis (against which health worker diagnoses were compared) was defined by applying Angola's malaria case management guideline to patient clinical signs and symptoms (assessed by surveyors, but information that should have been available to observed health workers) and laboratory data available to observed health workers (i.e., not the "gold standard" survey team's laboratory results). See Table 17.

[b] Diagnoses of the 24 patients were gastrointestinal illnesses (n=10; e.g., gastritis, intestinal parasites, and dysentery), respiratory illnesses (n=5; e.g., bronchitis), skin problems (n=2; e.g., scabies and skin boils), and other (n=7; e.g., dental caries, trauma, chicken pox, and malnutrition).

Table 28. Quality of malaria diagnosis according to the "new" policy (announced in September 2007) in outpatient health facilities, Huambo Province, Angola, October–November 2007

Characteristic and patient sub-group [question/variable in dataset]	No. and weighted percentage of patients	
	n	% (95% CI)
Health worker's malaria-related diagnosis for the 4 patient with a "gold standard" diagnosis[a] of complicated malaria [HW_DX_MALARIA2]		
Uncomplicated malaria (major error)	1	17.7% (NC)
No malaria[b] (major error)	3	82.3% (NC)
Health workers' malaria-related diagnoses for the 74 patients with a "gold standard" diagnosis[a] of uncomplicated malaria [HW_DX_MALARIA2]		
Complicated malaria (minor error)	2	2.0% (0–4.8)
Uncomplicated malaria (correct)	39	57.0% (42.0–71.9)
No malaria[c] (major error)	33	41.1% (25.9–56.3)
Health workers' malaria-related diagnoses for the 99 patients with a "gold standard" diagnosis[a] of no malaria [HW_DX_MALARIA2]		
Uncomplicated malaria (minor error)	33	28.3% (15.2–41.4)
No malaria (correct)	66	71.7% (58.6–84.8)
Overall quality of health workers' malaria-related diagnoses among all 177 patients [HW_MAL_DX_QUAL_NEW]		
Correct (health worker diagnoses of malaria and no malaria matched "gold standard" diagnoses)	105	61.8% (52.7–71.0)
Minor error (health worker incorrectly "over-diagnosed" uncomplicated malaria as complicated malaria, or over-diagnosed no malaria as uncomplicated malaria)	35	15.0% (7.2–22.7)
Major error (health worker incorrectly "under-diagnosed" complicated malaria as uncomplicated malaria, or under-diagnosed any type of malaria as no malaria)	37	23.2% (14.9–31.5)

Footnotes for Table 28.

CI = confidence interval; NC = not calculated.

[a] The "gold standard" malaria diagnosis (against which health worker diagnoses were compared) was defined by applying Angola's malaria case management guideline to patient clinical signs and symptoms (assessed by surveyors, but information that should have been available to the observed health workers) and laboratory data available to the observed health workers (i.e., not the "gold standard" survey team's laboratory results). See Table 20.

[b] Diagnoses of the 3 patients were respiratory illnesses (n=2; e.g., bronchitis) and malnutrition (n=1).

[c] Diagnoses of the 33 patients were gastrointestinal illnesses (n=13; e.g., diarrhea, dysentery, gastritis, and intestinal parasites), respiratory illnesses (n=7; e.g., bronchitis), otitis (n=2), skin problems (n=2; e.g., scabies and skin boils), and other (n=9; e.g., chicken pox, dental caries, malnutrition, and trauma).

Table 29. Quality of malaria[a] treatment according to the “old” (pre-September 2007) policy in outpatient health facilities, Huambo Province, Angola, October–November 2007

Characteristic and patient sub-group [question/variable in dataset]	No. and weighted percentage of patients	
	n	% (95% CI)
Quality of malaria treatment among all 177 patients (detailed description of prescribed treatments) [TX_AMA6]		
Complicated malaria treated with under-dosed AL (major error)	1	0.8% (0–2.4)
Uncomplicated malaria treated with correctly dosed AL (correct)	27	17.5% (9.9–25.2)
Uncomplicated malaria treated with correctly dosed quinine (minor error)	1	1.6% (0–4.5)
Uncomplicated malaria in a child <5kg treated with AL (with a dosage appropriate for a child 5–14 kg) (minor error)	1	0.4% (0–1.1)
Uncomplicated malaria not treated with antimalarials (major error)	26	14.5% (8.9–20.1)
Uncomplicated malaria treated with an ineffective[b] antimalarial (major error)	2	0.4% (0–1.1)
Uncomplicated malaria treated with under-dosed AL (major error)	1	0.6% (0–1.9)
No malaria and not treated with antimalarials (correct)	78	43.8% (32.3–55.4)
No malaria treated with AL (minor error)	32	16.2% (8.6–23.9)
No malaria treated with quinine (minor error)	4	0.7% (0–1.6)
No malaria treated with an ineffective[c] antimalarial (minor error)	4	3.4% (0–7.0)
Quality of malaria treatment among all 177 patients (general quality categories of prescribed treatments[d]) [TX_AMA5]		
Correct (recommended treatment)	105	61.4% (52.0–70.7)
Minor treatment error	42	22.3% (12.3–32.3)
Major treatment error	30	16.3% (10.2–22.4)

Table 29 continued on next page.

Table 29, continued. Quality of malaria[a] treatment according to the "old" (pre-September 2007) policy in outpatient health facilities, Huambo Province, Angola, October–November 2007

Characteristic and patient sub-group [question/variable in dataset]	No. and weighted percentage of patients	
	n	% (95% CI)
Quality of malaria treatment among the 59 patients with malaria (detailed description of prescribed treatments) [TX_AMA6]		
Complicated malaria treated with under-dosed AL (major error)	1	2.1% (0–6.6)
Uncomplicated malaria treated with correctly dosed AL (correct)	27	49.0% (33.5–64.5)
Uncomplicated malaria treated with correctly dosed quinine (minor error)	1	4.4% (0–12.2)
Uncomplicated malaria in a child <5kg treated with AL (with a dosage appropriate for a child 5–14 kg) (minor error)	1	1.0% (0–3.1)
Uncomplicated malaria not treated with antimalarials (major error)	26	40.5% (24.2–56.8)
Uncomplicated malaria treated with an ineffective[b] antimalarial (major error)	2	1.2% (0–3.1)
Uncomplicated malaria treated with under-dosed AL (major error)	1	1.7% (0–5.2)
Quality of malaria treatment among the 59 patients with malaria (general quality categories of prescribed treatments[d]) [TX_AMA5]		
Correct	27	49.0% (33.5–64.5)
Minor error	2	5.4% (0–13.2)
Major error	30	45.6% (28.2–63.1)
Quality of malaria treatment among the 59 patients with malaria (general quality categories in terms of antimalarials obtained by patients and patient recall of treatment instructions) [TX_AMA_PT]		
Patient left the health facility with the recommended antimalarial in hand and knowledge of how to administer the drug at home	17	27.1% (14.8–39.4)
Patient left the health facility with an adequate (but not recommended) antimalarial in hand and knowledge of how to administer the drug at home	2	5.4% (0–13.2)
Patient left the health facility and did not receive at least one of the following: an effective antimalarial, or adequate knowledge of how to administer the drug at home	40	67.5% (53.6–81.5)

Table 29 continued on next page.

Table 29, continued. Quality of malaria[a] treatment according to the "old" (pre-September 2007) policy in outpatient health facilities, Huambo Province, Angola, October–November 2007

Characteristic and patient sub-group [question/variable in dataset]	No. and weighted percentage of patients		
	n	%	(95% CI)
Quality of malaria treatment among the 118 patients without malaria (detailed description of prescribed treatments) [TX_AMA6]			
No malaria and not treated with antimalarials (correct)	78	68.3%	(54.6–82.0)
No malaria treated with AL (minor error)	32	25.3%	(13.9–36.7)
No malaria treated with quinine (minor error)	4	1.2%	(0–2.4)
No malaria treated with an ineffective[c] antimalarial (minor error)	4	5.3%	(0–11.1)
Quality of malaria treatment among the 118 patients without malaria (general quality categories of prescribed treatments[d]) [TX_AMA5]			
Correct	78	68.3%	(54.6–82.0)
Minor error	40	31.7%	(18.0–45.4)

Footnotes for Table 29.

AL = artemether-lumefantrine; CI = confidence interval.

[a] Malaria defined by applying Angola's malaria case management guideline to patient clinical signs and symptoms (assessed by surveyors, but information that should have been available to the observed health workers) and laboratory data available to the observed health workers (i.e., not the "gold standard" survey team's laboratory results).

[b] One patient treated with amodiaquine, and one patient treated with sulfadoxine-pyrimethamine.

[c] Three patients treated with amodiaquine, and one patient treated with chloroquine.

[d] No error means that patients received recommended treatment in exact accordance with guidelines (malaria cases treated with the recommended antimalarial with the recommended dosage, and non-malaria cases received no antimalarial treatment). Minor error means that malaria cases received non-recommended, but still life-saving, antimalarial treatment (either an overdose of a recommended antimalarial, or an adequate dose of a non-recommended antimalarial); and non-malaria cases received unnecessary antimalarial treatment that was unlikely to cause serious harm. Major error means that malaria cases did not receive life-saving treatment (no antimalarial, an ineffective antimalarial, or an under-dosed antimalarial).

Table 30. Quality of malaria[a] treatment according to the "new" policy (announced in September 2007) in outpatient health facilities, Huambo Province, Angola, October–November 2007

Characteristic and patient sub-group [question/variable in dataset]	No. and weighted percentage of patients	
	n	% (95% CI)
Quality of malaria treatment among all 177 patients (detailed description of prescribed treatments) [TX_AMA6_NEW]		
Complicated malaria treated with under-dosed AL (major error)	1	0.8% (0–2.4)
Complicated malaria not treated with antimalarials (major error)	3	3.6% (0–8.1)
Uncomplicated malaria treated with correctly dosed AL (correct)	33	21.5% (12.4–30.6)
Uncomplicated malaria treated with correctly dosed quinine (minor error)	1	1.6% (0–4.5)
Uncomplicated malaria in a child <5kg treated with AL (with a dosage appropriate for a child 5–14 kg) (minor error)	1	0.4% (0–1.1)
Uncomplicated malaria not treated with antimalarials (major error)	35	20.3% (14.5–26.1)
Uncomplicated malaria treated with an ineffective[b] antimalarial (major error)	3	1.6% (0–4.0)
Uncomplicated malaria treated with under-dosed AL (major error)	1	0.6% (0–1.9)
No malaria and not treated with antimalarials (correct)	66	34.5% (26.7–42.2)
No malaria treated with AL (minor error)	26	12.3% (5.0–19.6)
No malaria treated with quinine (minor error)	4	0.7% (0–1.6)
No malaria treated with an ineffective[b] antimalarial (minor error)	3	1.5% (0–5.3)
Quality of malaria treatment among all 177 patients (general quality categories of prescribed treatments[c]) [TX_AMA5_NEW]		
Correct (recommended treatment)	99	56.0% (47.9–64.1)
Minor treatment error	35	17.2% (9.4–25.0)
Major treatment error	43	26.5% (18.5–35.2)

Table 30 continued on next page.

Table 30, continued. Quality of malaria[a] treatment according to the "new" policy (announced in September 2007) in outpatient health facilities, Huambo Province, Angola, October–November 2007

Characteristic and patient sub-group [question/variable in dataset]	No. and weighted percentage of patients	
	n	% (95% CI)
Quality of malaria treatment among the 78 patients with malaria (detailed description of prescribed treatments) [TX_AMA6_NEW]		
Complicated malaria treated with under-dosed AL (major error)	1	1.5% (0–4.7)
Complicated malaria not treated with antimalarials (major error)	3	7.1% (0–16.3)
Uncomplicated malaria treated with correctly dosed AL (correct)	33	42.7% (27.4–58.1)
Uncomplicated malaria treated with correctly dosed quinine (minor error)	1	3.1% (0–8.7)
Uncomplicated malaria in a child <5kg treated with AL (with a dosage appropriate for a child 5–14 kg) (minor error)	1	0.7% (0–2.2)
Uncomplicated malaria not treated with antimalarials (major error)	35	40.4% (27.5–53.2)
Uncomplicated malaria treated with an ineffective[b] antimalarial (major error)	3	3.2% (0–8.0)
Uncomplicated malaria treated with under-dosed AL (major error)	1	1.2% (0–3.7)
Quality of malaria treatment among the 78 patients with malaria (general quality categories of prescribed treatments[c]) [TX_AMA5_NEW]		
Correct	33	42.7% (27.4–58.1)
Minor error	2	3.8% (0–9.5)
Major error	43	53.4% (35.1–71.8)

Table 30 continued on next page.

Table 30, continued. Quality of malaria[a] treatment according to the "new" policy (announced in September 2007) in outpatient health facilities, Huambo Province, Angola, October–November 2007

Characteristic and patient sub-group [question/variable in dataset]	No. and weighted percentage of patients		
	n	%	(95% CI)
Quality of malaria treatment among the 99 patients without malaria (detailed description of prescribed treatments) [TX_AMA6_NEW]			
No malaria and not treated with antimalarials (correct)	66	69.3%	(56.2–82.5)
No malaria treated with AL (minor error)	26	24.7%	(11.9–37.5)
No malaria treated with quinine (minor error)	4	1.5%	(0–3.1)
No malaria treated with an ineffective antimalarial (minor error)	3	4.4%	(0–11.1)
Quality of malaria treatment among the 99 patients without malaria (general quality categories of prescribed treatments[c]) [TX_AMA5]			
Correct	66	69.3%	(56.2–82.5)
Minor error	33	30.7%	(17.5–43.8)

Footnotes for Table 30.

AL = artemether-lumefantrine; CI = confidence interval.

[a] Malaria defined by applying Angola's malaria case management guideline to patient clinical signs and symptoms (assessed by surveyors, but information that should have been available to the observed health workers) and laboratory data available to the observed health workers (i.e., not the "gold standard" survey team's laboratory results).

[b] Four patients treated with amodiaquine, one treated with sulfadoxine-pyrimethamine, and one treated with chloroquine.

[c] No error means that patients received recommended treatment in exact accordance with guidelines (malaria cases treated with the recommended antimalarial with the recommended dosage, and non-malaria cases received no antimalarial treatment). Minor error means that malaria cases received non-recommended, but still life-saving, antimalarial treatment (either an overdose of a recommended antimalarial, or an adequate dose of a non-recommended antimalarial); and non-malaria cases received unnecessary antimalarial treatment that was unlikely to cause serious harm. Major error means that malaria cases did not receive life-saving treatment (no antimalarial, an ineffective antimalarial, or an under-dosed antimalarial).

Table 31. AL use among the 62 patients who were tested by the observed health worker and who had results available by the end of the health facility visit in outpatient health facilities, Huambo Province, Angola, October–November 2007

Characteristic and patient sub-group [question/variable in dataset]	No. and weighted percentage of patients		
	n	%	(95% CI)
Treatment for the 17 patients tested by the observed health worker (with microscopy or RDT) and had a positive result [TESTRES, TX_AMA8]			
AL correctly dosed[a]	15	84.7%	(59.0–100)
AL under-dosed[b]	1	11.5%	(0–35.9)
Ineffective antimalarial prescribed	1	3.8%	(0–12.7)
Treatment for the 45 patients tested by the observed health worker (with microscopy or RDT) and had a negative result [TESTRES, TX_AMA8]			
No antimalarial prescribed (correct)	18	43.8%	(23.3–64.2)
AL prescribed (minor error)	22	44.7%	(24.1–65.4)
Ineffective antimalarial prescribed (minor error)	2	9.1%	(0–24.4)
Quinine prescribed (minor error)	3	2.4%	(0–5.5)

Footnotes for Table 31.

AL = artemether-lumefantrine; CI = confidence interval.

[a] None of these patients had a sign of severe illness; thus, we judged these to be uncomplicated malaria cases that should have been treated with AL.

[b] This patient had a sign of severe illness, but the under-dosed AL means that the patient was incorrectly treated regardless of the malaria severity.

Table 32. Analysis of the concordance of health worker malaria diagnoses and prescribed treatments in outpatient health facilities, Huambo Province, Angola, October–November 2007

Characteristic and patient sub-group [question/variable in dataset]	No. and weighted percentage of patients	
	n	% (95% CI)
Treatment for the 2 patients whom health workers diagnosed with complicated malaria [HW_DX_MALARIA2, TX_AMA9]		
AL prescribed (major error)	2[a]	100% (NC)
Treatment for the 73 patients whom health workers diagnosed with uncomplicated malaria [HW_DX_MALARIA2, TX_AMA9]		
AL prescribed (correct)[b]	59	83.6% (74.7–92.6)
Quinine prescribed (minor error)	5	5.6% (0–12.4)
AL prescribed inappropriately because patient weighed <5 kg (minor error)	1	0.9% (0–2.7)
Ineffective antimalarial prescribed (major error)	5	5.5% (0–12.0)
No antimalarial prescribed (major error)	3	4.4% (0–10.9)
Treatment for the 102 patients whom health workers did not diagnose with malaria [HW_DX_MALARIA2, TX_AMA9]		
No antimalarial prescribed (correct)	101	97.3% (92.0–100)
Ineffective antimalarial prescribed (minor error)	1	2.7% (0–8.0)

Footnotes for Table 32.

AL = artemether-lumefantrine; CI = confidence interval; NC = not calculated.

[a] One of these patients was seen in a health facility without injectable quinine in stock

[b] Dosage not considered in this analysis because health workers almost always prescribed AL correctly (see Table 33).

Table 33. Use of AL: frequency of prescription, and appropriateness of dosing and counseling (whether or not AL was indicated, according to guidelines) in outpatient health facilities, Huambo Province, Angola, October–November 2007

Characteristic and patient sub-group [question/variable in dataset]	No. and weighted percentage of patients	
	n	% (95% CI)
AL prescribed (whether or not indicated, according to guidelines) among all 177 patients [GOT_COARTEM, A13a1]	62[a]	35.5% (24.9–46.2)
Dosing for the 62 patients who received AL [DOSE_COARTEM, A13a1-4]		
Correctly dosed	59	95.1% (89.2–100)
Underdosed	2	3.9% (0–9.5)
Overdosed	0	0% (NC)
AL not recommended (weight <5 kg)	1	1.0% (0–3.1)
First AL dose given during consultation (for the 62 patients who received AL) [GOT_FIRST_AL_DOSE, A12a]	9	10.7% (1.2–20.3)
Quality of counseling for the 62 patients who received AL		
HW gave complete dosing instructions (definition of a dose, no. of doses/day, and treatment duration)[b] [A13A5]	55	88.2% (78.3–98.2)
HW advised to take the medicine with food [A14a1]	17	31.3% (12.3–50.2)
Patient could repeat all dosing instructions given by the HW (definition of a dose, no. of doses/day, and treatment duration[c]) (even if HW's dosage was incorrect) [UNDERSTOOD_COARTEM]	43	60.9% (44.2–77.6) [Note: 2 missing values]
HW advised to take the medicine with milk or fat-containing food [A14a2]	4	4.9% (0–10.8)
HW advised to return for a follow-up visit [A14f]	8	14.4% (0.4–28.3)
HW advised to sleep under a bed net to prevent malaria [A14d]	0	0% (NC)
HW advised to return to the health facility if the patient becomes seriously ill [A14e]	5	5.8% (0–11.8)
HW advised to complete all the treatment (take all medicines) [A14g]	41	69.9% (51.4–88.4)

Footnotes for Table 33 on the next page.

Footnotes for Table 33.

AL = artemether-lumefantrine; CI = confidence interval; HW = health worker; NC = not calculated.

[a] Of these 62 patients, 59 actually had AL in hand; for the other 3 patients, AL had been prescribed but the medicines were not given.

[b] Dosing instructions were considered complete even if the dosage was incorrect, although in nearly all cases (53 of the 55 patients) the dosage was correct.

[c] The response "*ate que termine*" (until all the medicines are done) was considered a correct response for treatment duration.

Table 34. Satisfaction of patients seen in outpatient health facilities, Huambo Province, Angola, October–November 2007

Characteristic/question<br>[question/variable in dataset]	No. and weighted percentage of patients		
	n	%	(95% CI)
In general, which statement best describes how you feel about the services you received today at the health facility? (N=177) [B8A]			
I was very satisfied	138	78.4%	(69.1–87.7)
I was somewhat satisfied	25	11.3%	(5.9–16.7)
I was somewhat dissatisfied	10	6.1%	(1.4–10.9)
I was very dissatisfied	4	4.2%	(0–10.3)
Please tell me one thing that you think would improve the care at this health facility? (N=175 patients; 2 missing) [B8B]			
No opinion or suggestion	116	61.4 %	
"I am happy with attention from health workers."	21	15.4 %	
Improve access to medicines	11	7.1 %	
Improve patient management	8	4.4%	
Improve the quality of treatment	7	3.7 %	
Improve the quality of diagnostics	4	3.1 %	
Increase the number of health workers	3	2.1 %	
Other ("I'd like them to have an ambulance" and 4 other non-specific comments)	5	2.7 %	

Footnote for Table 34.

CI = confidence interval.

Table 35. Malaria-related knowledge and reported practices of patients seen in outpatient health facilities, Huambo Province, Angola, October–November 2007

Characteristic and patient sub-group [question/variable in dataset]	No. and weighted percentage of patients		
	n	%	(95% CI)
What is the most important way that a person gets malaria? [B9] (Patient allowed to give more than one response)			
Mosquitoes, or a mosquito bite [B9A]	49	32.6%	(23.8–41.3)
Not sleeping under a mosquito net	2	2.9%	
Insects or an insect bite (mosquito not mentioned) [B9B]	4	1.0%	(0–2.3)
The sun, witchcraft, sorcery, or magic [B9C, B9D]	0	0%	NC
Other[a] [B9E]	33	21.4%	(11.8–31.0)
Do not know [B9F]	102	51.3%	(37.8–64.8)
How can you protect yourself from getting malaria? [B10]			
Sleep under an insecticide-treated bednet [B10A]	13	10.1%	(4.1–16.1)
Sleep under a bednet (insecticide not mentioned) [B10B]	38	23.8%	(14.7–32.9)
Take medicine [B10C]	9	4.0%	(0–8.1)
Other[b] [B10E]	45	27.8%	(11.0-44.5)
Do not know [B10F]	90	49.9%	(36.3–63.4)
Did (you/your child) sleep under a bednet last night? [B11]			
Yes	62	40.5%	(29.1–51.8)
No	112	58.2%	(46.7–69.7)
Do not know	3	1.3%	(0–3.3)

Footnotes for Table 35.

CI = confidence interval; NC = not calculated.

[a] These 33 patients gave the following 39 responses: trash (n=7), standing or dirty water (n=7), eat poorly or eat certain kinds of food (n=5), contact with rain (n=4), poor hygiene/live badly (n=4), walk barefoot/walk on cold ground (n= 4), and other (n=8) ["when I have fever", "cover head", "don't use mosquito net", "poorly treat child with medicine", "not allowed to jump in water or rain", and "lots of work"].

[b] These 45 patients gave the following 48 responses: keep clean or avoid trash (n=12), avoid dirty or standing water (n=12), medicines (n=5), mosquito control (close doors or windows, use a mosquito net) (n=4), go to hospital (n=4), good hygiene (n=5), healthy eating (n=3), other (n=3); 1 missing response.

Table 36. Design effects and intraclass correlations (ρ) of selected indicators (Table 36a) and a summary of findings (Table 36b).

Note that the no. of clusters for entire survey was 33 (and for all indicators with a denominator of 177). For AL indicators with a denominator of 62, there were 27 clusters; for the AL indicator with N=60 (2 missing values), there were 26 clusters.

Indicator [variable in the dataset]	n/N	Mean cluster size, m	Weighted			Unweighted		
			%	Design effect	Intraclass correlation, ρ[a]	%	Design effect	Intraclass correlation, ρ[a]
**Assessment indicators (N=10)**								
Determine history of fever [DET_FEV]	152/177	5.36	87.6	1.8843	0.2028	85.9	1.3225	0.0740
Determine headache [DET_HA]	76/177	5.36	39.2	1.8022	0.1840	42.9	1.2701	0.0619
Determine joint pain [DET_JP]	52/177	5.36	23.1	1.9811	0.2250	29.4	0.9806	-0.0044
Determine vomiting [DET_VOM]	47/177	5.36	24.1	2.4521	0.3331	26.6	1.3627	0.0832
Determine chills [DET_CHILLS]	5/177	5.36	3.7	1.3828	0.0878	2.8	1.2348	0.0539
Determine poor appetite [DET_ANOREX]	56/177	5.36	26.8	1.8790	0.2016	31.6	1.0991	0.0227
Determine fatigue [DET_FATIG]	5/177	5.36	1.8	1.0495	0.0114	2.8	1.2706	0.0621
Determine diarrhea [DET_DIAR]	61/177	5.36	37.5	1.5272	0.1209	34.5	0.8607	-0.0319
Measure patient's temperature [A9a]	122/177	5.36	74.1	3.1072	0.4833	68.9	1.9204	0.2111
Check for anemia [DET_ANEMIA]	23/177	5.36	16.2	2.7947	0.4116	13.0	1.0976	0.0224
**Diagnosis and treatment indicators (N=7)**								
Patient correctly selected for testing (old policy) [COR_TEST_OLD]	93/177	5.36	41.5	2.2160	0.2789	52.4	1.5943	0.1363
Patient correctly selected for testing (new policy) [COR_TEST_NEW]	99/177	5.36	56.3	3.3629	0.5419	55.9	1.4280	0.0982
Correct malaria diagnosis (old policy) [HW_MAL_DX_QUAL_OLD]	110/177	5.36	66.1	1.1378	0.0316	62.1	1.0662	0.0152
Correct malaria diagnosis (new policy) [HW_MAL_DX_QUAL_NEW]	105/177	5.36	61.8	1.5131	0.1177	59.3	0.8427	-0.0361
Correct malaria treatment (old policy) [TX_AMA5]	105/177	5.36	61.4	1.5696	0.1306	59.3	1.0168	0.0039
Correct malaria treatment (new policy) [TX_AMA5_NEW]	99/177	5.36	56.0	1.1276	0.0293	55.9	0.8121	-0.0431
Patient prescribed AL got first dose [GOT_FIRST_AL_DOSE, A12a]	9/62	2.30	10.7	1.3616	0.2782	14.5	1.3210	0.2469

Table 36a continued on next page.

Table 36, continued. Design effects and intraclass correlations of selected indicators (Table 36a) and summary of findings (Table 36b)

Indicator [variable in the dataset]	n/N	Mean cluster size, m	Weighted			Unweighted		
			%	Design effect	Intraclass correlation, ρ[a]	%	Design effect	Intraclass correlation, ρ[a]
**Counseling indicators for pts receiving AL (N=8)**								
HW gave complete dosing instructions [A13A5]	55/62	2.30	88.2	1.3749	0.2884	88.7	1.1357	0.1044
HW advised to take the medicine with food [A14a1]	17/62	2.30	31.3	2.4169	1.0899[b]	27.4	1.2498	0.1922
Patient could repeat all dosing instructions [UNDERSTOOD_COARTEM]	43/60	2.31	60.9	1.6292	0.4803	71.7	0.9815	-0.0141
HW advised to take AL with milk or fat-containing food [A14a2]	4/62	2.30	4.9	1.1093	0.0841	6.5	1.5167	0.3975
HW advised to return for a follow-up visit [A14f]	8/62	2.30	14.4	2.2787	0.9836	12.9	1.4605	0.3542
HW advised to sleep under a bed net to prevent malaria [A14d]	0/62	2.30	0	No DE	No ρ	0	No DE	No ρ
HW advised to return to HF if patient becomes seriously ill [A14e]	5/62	2.30	5.8	0.9713	-0.0221	8.1	0.9298	-0.0540
HW advised to complete the treatment (take all medicines) [A14g]	41/62	2.30	69.9	2.3413	1.0318[b]	66.1	1.9193	0.7072

Summary of 24 indicators (1 indicator excluded because value=0% and thus no DE or intraclass correlation coefficient)

Summary statistic	Weighted		Unweighted		Ratio of $DE_{weighted}$ / $DE_{unweighted}$
	Design effect	Intraclass correlation, ρ	Design effect	Intraclass correlation, ρ	
Mininum	0.9713	-0.0221	0.8121	-0.0540	0.73
Maximum	3.3629	1.0899[b]	1.9204	0.7072	2.55
Median	1.7157	0.2139	1.2423	0.0620	1.48
Mean	1.8446	0.3169	1.2372	0.1110	1.51

Footnotes for Table 36:

AL = Artemether-lumefantrine; DE = design effect; HF = health facility; HW = health worker.

[a] $\rho = (DE - 1) / (m - 1)$, from the formula: $DE = \rho (m - 1) + 1$, where m = mean cluster size.

[b] Logically, ρ cannot be >1; this result may be due to a rounding error or small imperfections in SAS's method for estimating DE.

Figure 1. Map of Huambo Province, Angola, with approximate locations of sampled health facilities (gray circles)

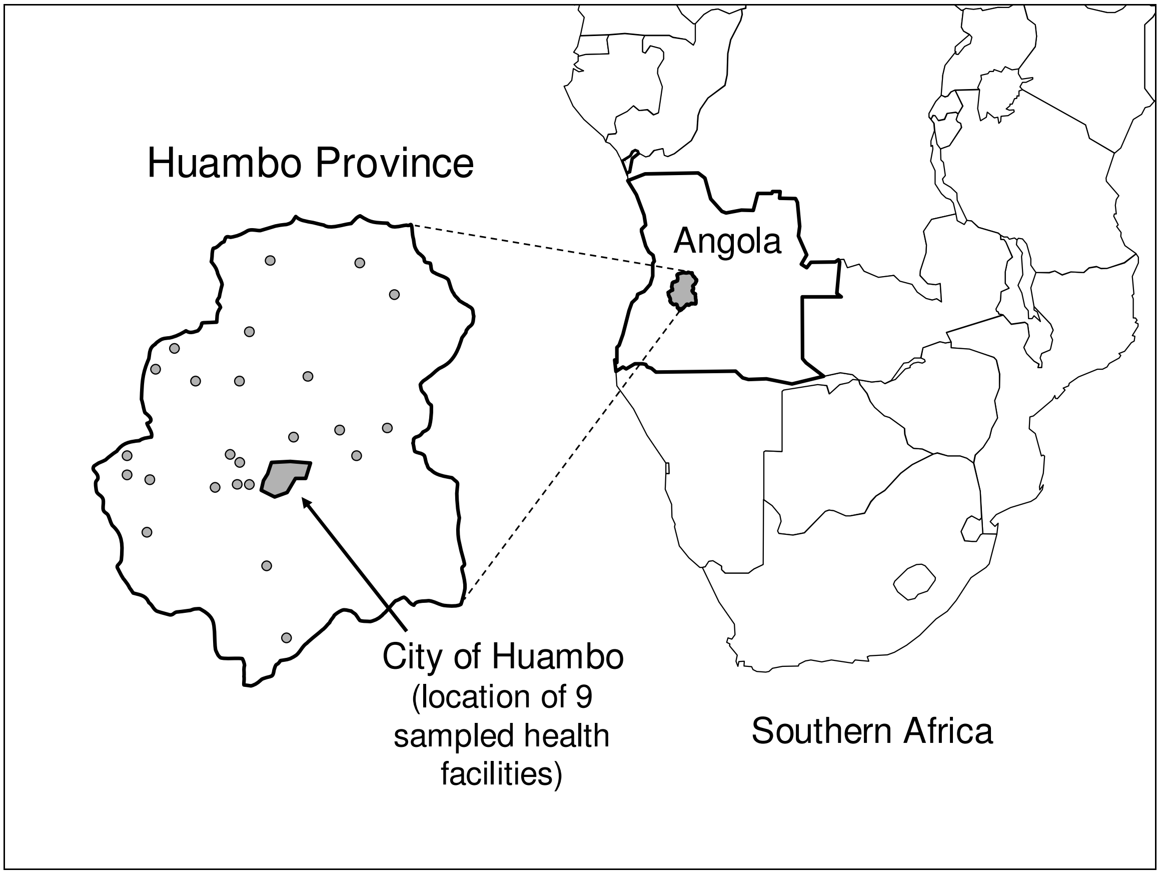

Figure 2. Case-management algorithm[a] in training materials used in Huambo, Angola, to teach the "old" (pre-September 2007) policy

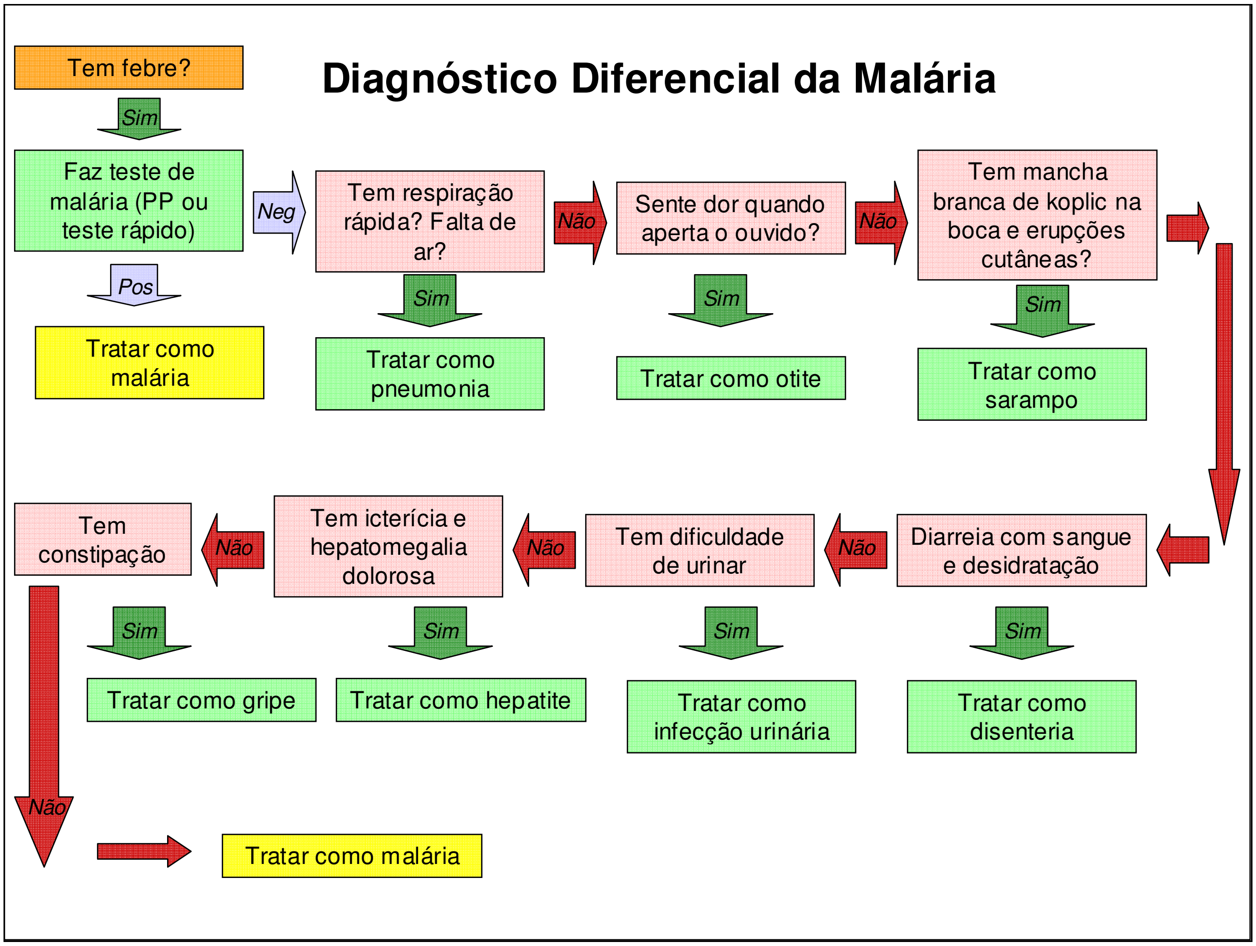

<u>Footnote for Figure 2.</u>

[a] The source for this figure is Slide 10 of a Powerpoint presentation used to train health workers, titled "*Formação de Técnicos sobre: Manuseamento de Casos*" (filename = "ManuseamentodeCasosv3 CORRIGIDO.ppt", date = August 15, 2007). Slide 1 of the presentation shows the logos of the MENTOR Initiative, the Angolan Ministry of Health, and Roll Back Malaria. This algorithm also appears in the Powerpoint presentation with the filename "Fluxograma modificado para formação Huambo.ppt" (date = August 15, 2007).

Figure 3. Algorithm used to analyze the quality of malaria diagnosis and treatment according to the "old" policy (pre-September 2007), as it was applied in Huambo Province, Angola

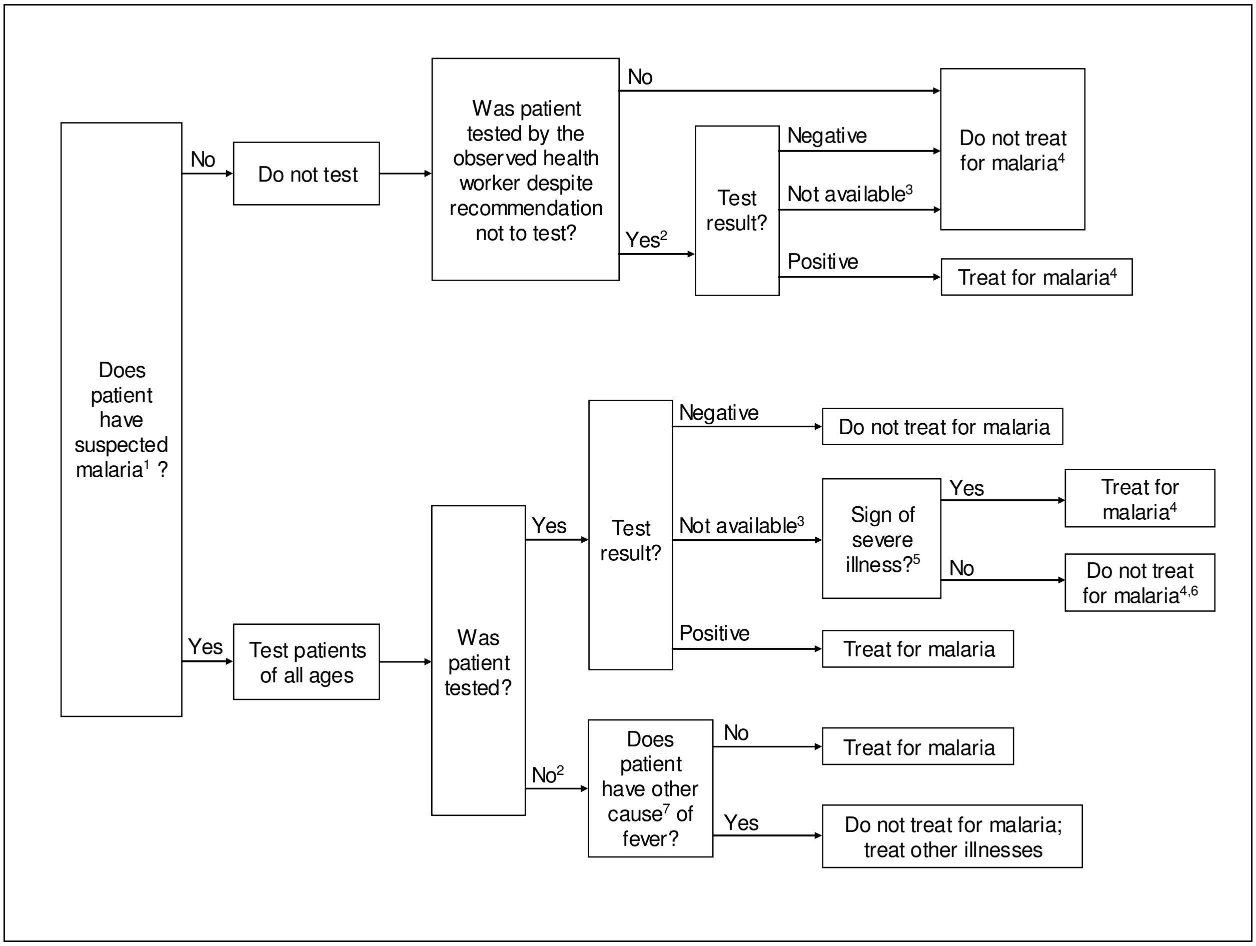

<u>Footnotes for Figure 3.</u>

1. Defined as either fever (history of fever <u>or</u> axillary temperature >37.5ºC), <u>or</u> at least 3 of the following: headache, joint pain, chills, sweating, anemia (palmor pallor), cough (applies to children only), anorexia, fatigue, vomiting, or diarrhea.

2. Error (health worker's decision did not follow policy documents and training materials).

3. Result not available on the day of the consultation, patient asked to return the next day (this only occurred for 2 patients).

4. This part of the algorithm was not explicitly included in policy documents or training materials; however, the decision could be logically inferred from policy documents or training materials.

5. Defined as cerebral dysfunction, cerebral malaria, disseminated intravascular coagulopathy, hemoglobinuria, hepatic dysfunction, hyperthermia, pulmonary edema, renal insufficiency, severe anemia, or shock. For details, see Box 1.

6. Do not treat for malaria now; wait until result is ready and treat only if test is positive.

7. Defined as dysentery, hepatitis, influenza-like illness, measles, otitis, pneumonia, or urinary tract infection. For details, see Box 2.

Figure 4. Malaria case management algorithm in health facilities that were able to perform laboratory testing for malaria according to the "new" policy (announced in September 2007) that is expected to be used in all parts of Angola with hyper- or meso-endemic malaria transmission

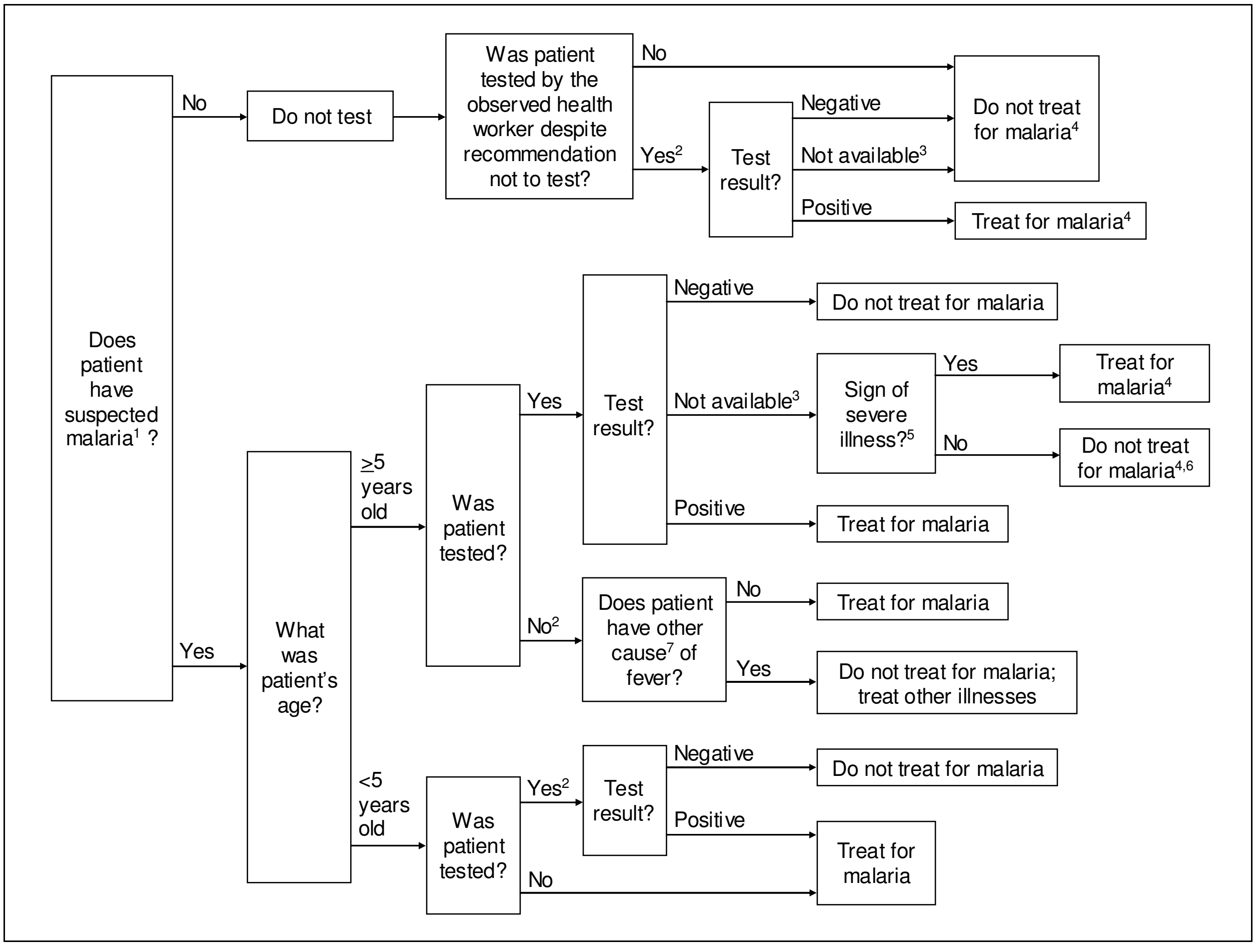

<u>Footnotes for Figure 4.</u>

1. Defined as either fever (history of fever <u>or</u> axillary temperature >37.5ºC), <u>or</u> at least 3 of the following: headache, joint pain, chills, sweating, anemia (palmor pallor), cough (applies to children only), anorexia, fatigue, vomiting, or diarrhea.

2. Error (health worker's decision did not follow policy documents and training materials).

3. Result not available on the day of the consultation, patient asked to return the next day (this only occurred for 2 patients).

4. This part of the algorithm was not explicitly included in policy documents or training materials; however, the decision could be logically inferred from policy documents or training materials.

5. Defined as cerebral dysfunction, cerebral malaria, disseminated intravascular coagulopathy, hemoglobinuria, hepatic dysfunction, hyperthermia, pulmonary edema, renal insufficiency, severe anemia, or shock. For details, see Box 1.

6. Do not treat for malaria now; wait until result is ready and treat only if test is positive.

7. Defined as dysentery, hepatitis, influenza-like illness, measles, otitis, pneumonia, or urinary tract infection. For details, see Box 2.

Figure 5. Graphical pathway analysis of the case-management process according to the "old" (pre-September 2007) policy for 40 patients without febrile illness/suspected malaria and therefore no malaria (Figure 5a), 78 patients with febrile illness/suspected malaria but no gold standard malaria diagnosis (Figure 5b), and 59 patients with a gold standard malaria diagnosis (Figure 5c), Huambo Province, Angola, October–November 2007

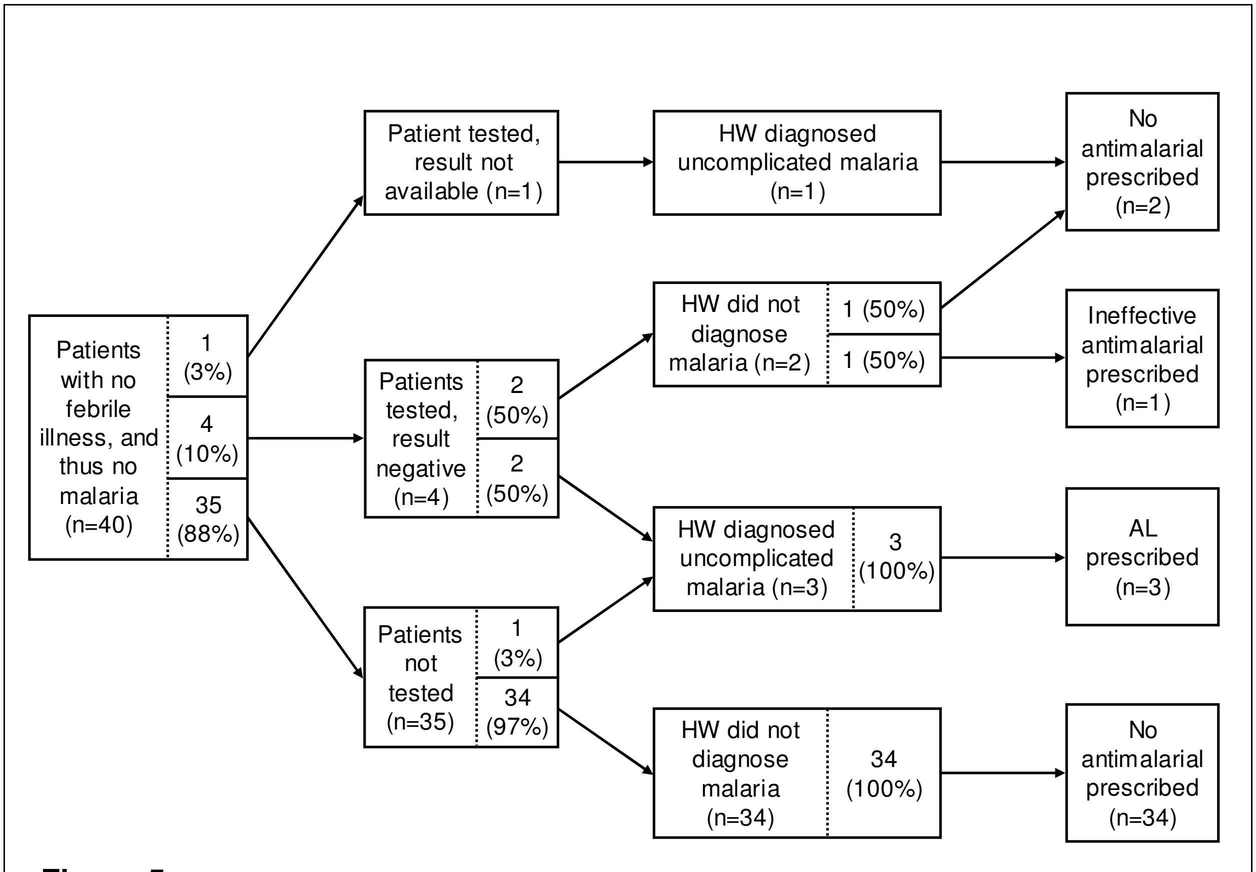

Figure 5a

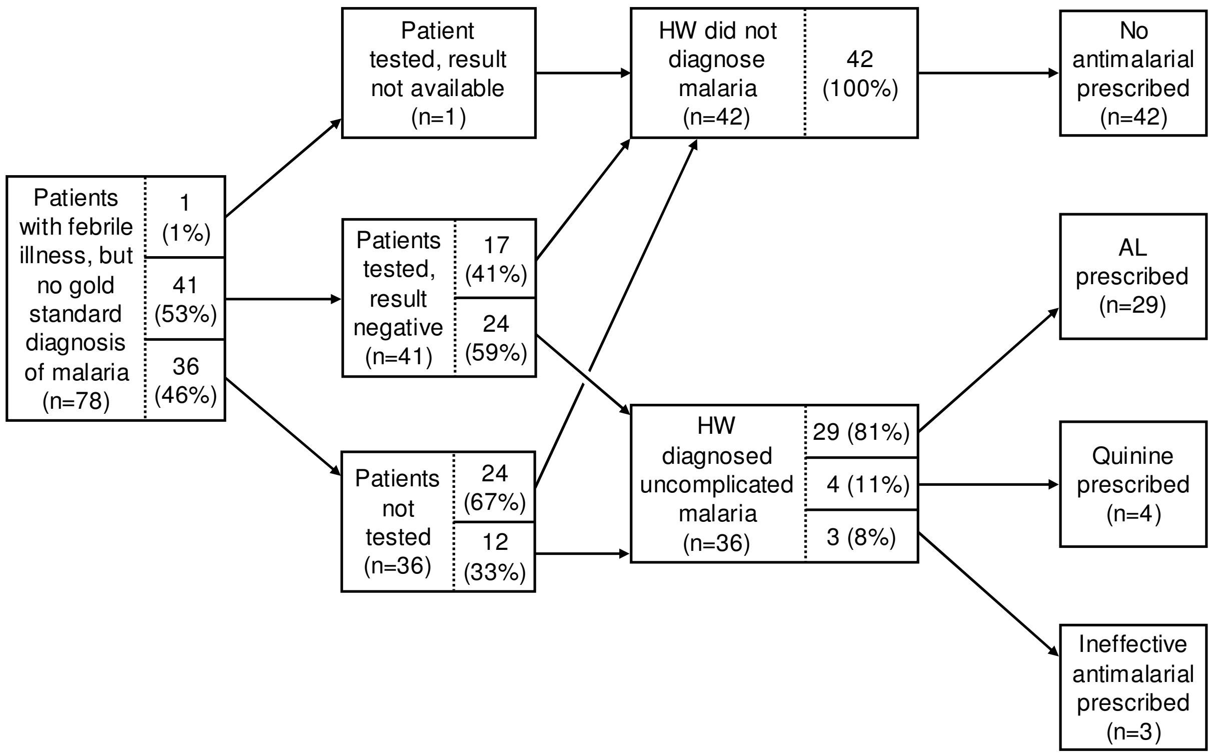
Patients with febrile illness, but no gold standard diagnosis of malaria (n=78)
1 (1%)
41 (53%)
36 (46%)
Patient tested, result not available (n=1)
Patients tested, result negative (n=41)
17 (41%)
24 (59%)
Patients not tested (n=36)
24 (67%)
12 (33%)
HW did not diagnose malaria (n=42)
42 (100%)
HW diagnosed uncomplicated malaria (n=36)
29 (81%)
4 (11%)
3 (8%)
No antimalarial prescribed (n=42)
AL prescribed (n=29)
Quinine prescribed (n=4)
Ineffective antimalarial prescribed (n=3)

Figure 5b

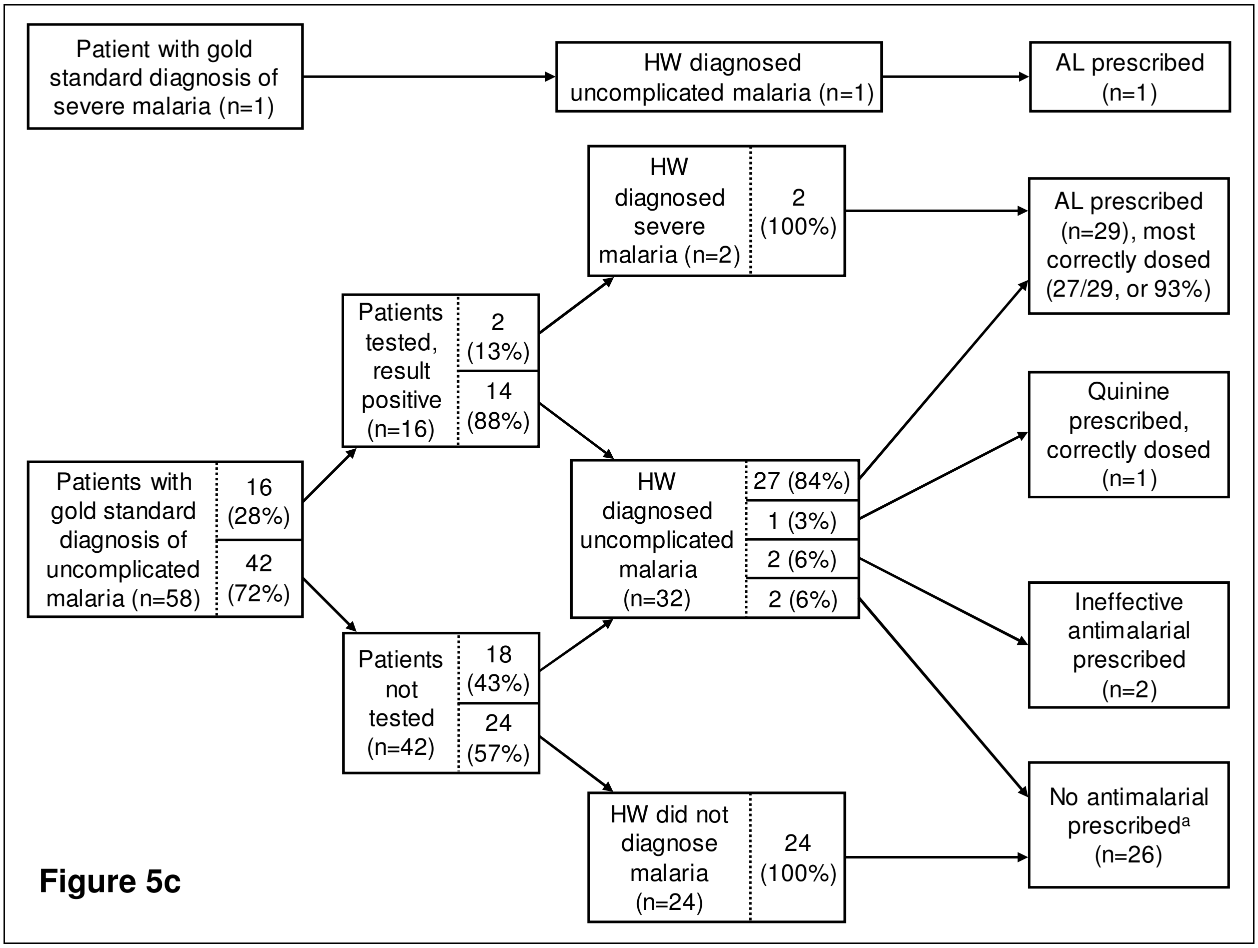

Figure 5c

Footnote for Figure 5.

[a] None of these 26 patients had been tested.

Figure 6. Causal diagram of the case-management process according to the "old" (pre-September 2007) policy, Huambo Province, Angola, October–November 2007

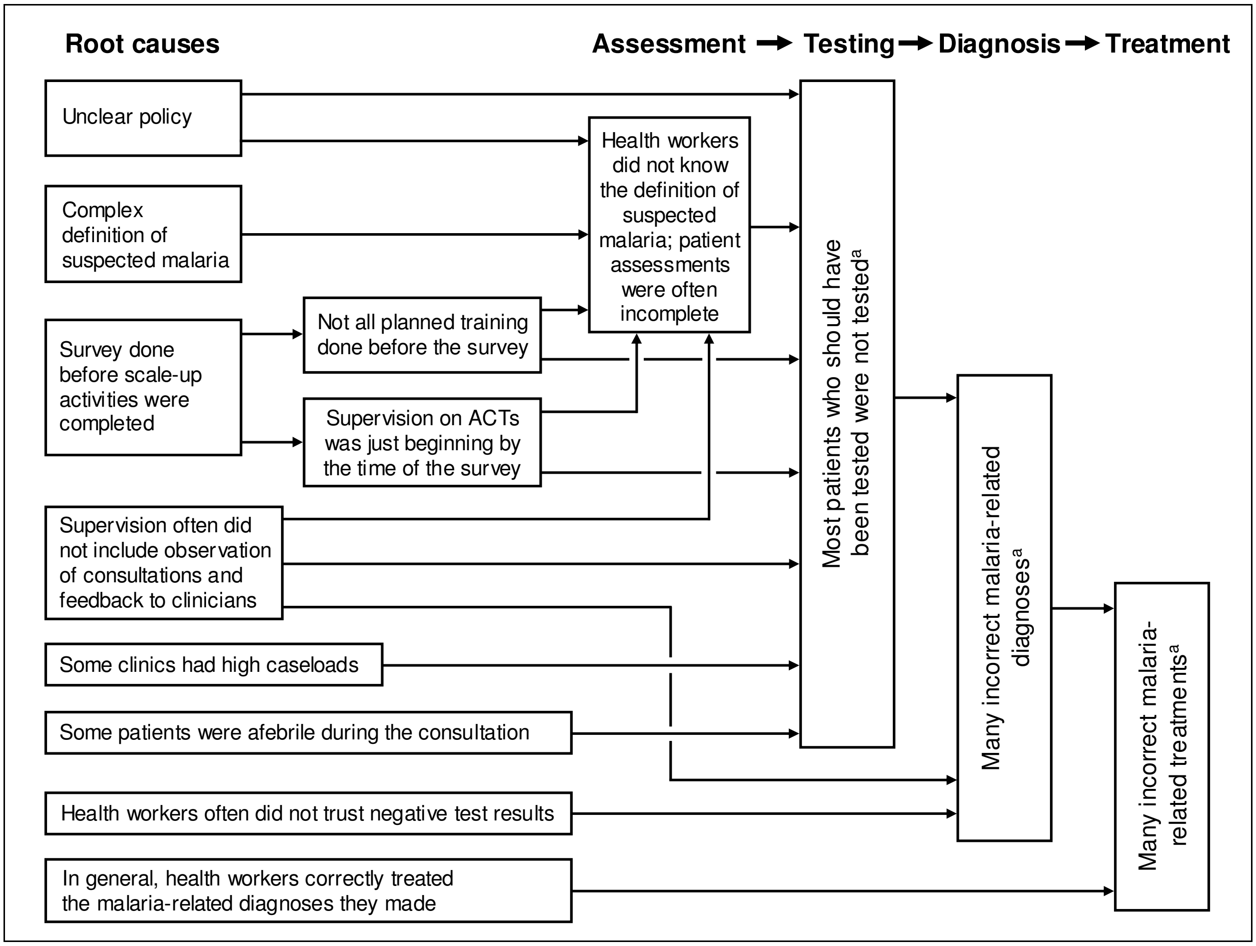

Footnotes for Figure 6.

[a] According to the "old" (pre-September 2007) policy.

Figure 7. Hypothetical relationship between the quality of health worker practices and the timing of scale-up efforts

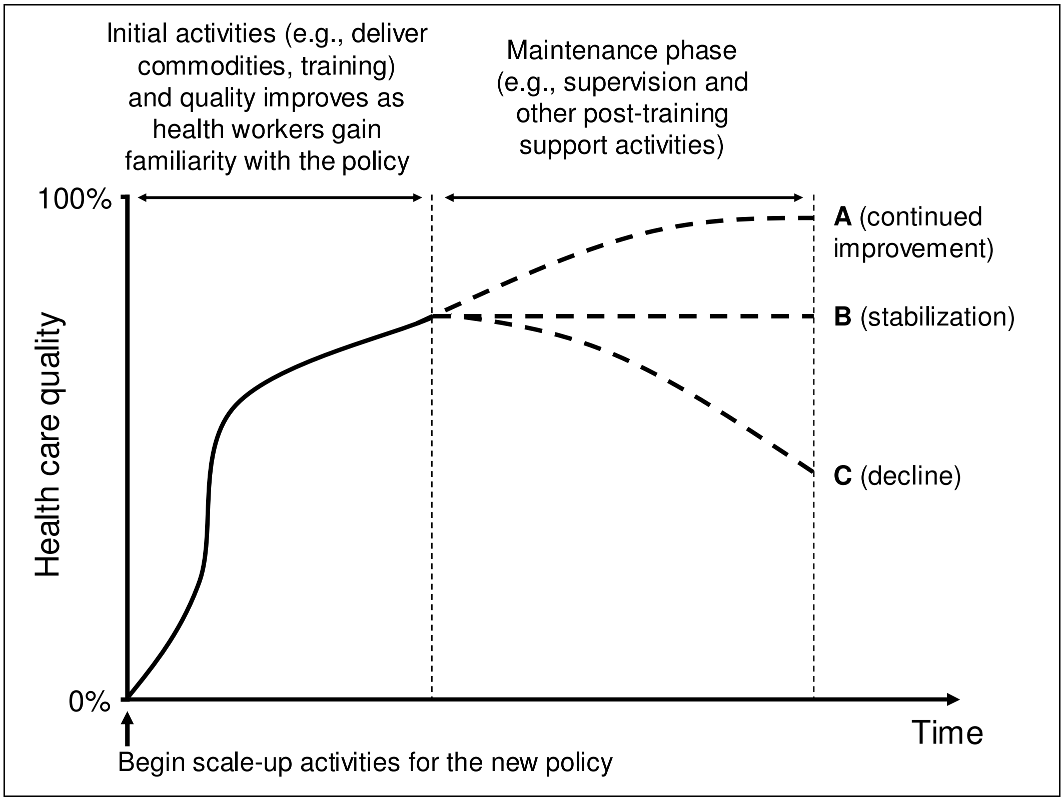

ANNEXES

Annex 1. Form for determining patient eligibility

Annex 2. Observation checklist

Annex 3. Questionnaire for patient interview

Annex 4a. Re-examination form for patients <5 years old

Annex 4b. Re-examination form for patients ≥5 years old

Annex 5. Health worker interview

Annex 6. Health facility assessment

Annex 7. Verbal consent for health workers

Annex 8. Verbal consent for patients

Annex 9. Assessing health worker knowledge

Annex 1. Form for determining patient eligibility

This form is to be used by the survey team driver. If there are any questions, ask the team supervisor. It is important to interview all people coming to the health facility. If you must leave your post, ask the supervisor to assign someone to replace you while you are away.

Health facility identification number: ___ ___ ___ Date: ____ / ____ / _______
(*day/month/year*)

Health facility name: ____________________________________

Read to person: Hello. My name is (name), and I am helping to conduct a survey on health issues. You might be eligible to be in the survey. I would like to ask you 3 questions. It will only take a moment.

Ask each person who comes to the facility the following questions:

1. Are you here to see a health worker?
2. *If yes to question 1, ask:* Are you here to see a health worker because you are sick (or your child is sick)?
3. *If yes to question 2, ask:* Is this your first (initial) visit to this health facility for this illness?

**Response to Question 1**	**Response to Question 2**	**Response to Question 3**	**Action**
**NO**	*Skip*	*Skip*	1. Thank person and continue with next person in line.
**YES**	**NO**	*Skip*	
**YES**	**YES**	**NO**	1. Circle number on tally sheet for **follow-up** visits.<br>2. Do not give an identification card to the person.<br>3. Thank person and continue with next person in line.
**YES**	**YES**	**YES**	1. Circle number on tally sheet for **initial** visits.<br>2. If the number on the tally sheet is a "yellow" number, then, give the patient an identification card to the person. Tell the person that a surveyor will come to speak with (him/her) shortly.<br>3. Thank person and continue with next person in line.

Tally sheet for **initial** consultations

1	2	3	4	5	6	7	8	9	10
11	12	13	14	15	16	17	18	19	20
21	22	23	24	25	26	27	28	29	30
31	32	33	34	35	36	37	38	39	40
41	42	43	44	45	46	47	48	49	50
51	52	53	54	55	56	57	58	59	60
61	62	63	64	65	66	67	68	69	70
71	72	73	74	75	76	77	78	79	80
81	82	83	84	85	86	87	88	89	90
91	92	93	94	95	96	97	98	99	100
101	102	103	104	105	106	107	108	109	110
111	112	113	114	115	116	117	118	119	120
121	122	123	124	125	126	127	128	129	130
131	132	133	134	135	136	137	138	139	140
141	142	143	144	145	146	147	148	149	150
151	152	153	154	155	156	157	158	159	160
161	162	163	164	165	166	167	168	169	170

Tally sheet for **follow-up** consultations.

1	2	3	4	5	6	7	8	9	10
11	12	13	14	15	16	17	18	19	20
21	22	23	24	25	26	27	28	29	30
31	32	33	34	35	36	37	38	39	40
41	42	43	44	45	46	47	48	49	50

Annex 2. Observation checklist

A1. Health facility identification number: ___ ___ ___

A2. Health worker's name: ____

A3. Health worker identification number: ___ ___

A4. Patient's name: ______________________________________

A4a. Patient's sex: [] male (1) [] female (2)

A5. Patient's identification number: ___ ___ ___

A6. Date: ____ / ____ / _______ (*day/month/year*)

Observations. *Observe silently, and do not interfere with the consultation. Record what you hear and see.*

A7. What reasons does the patient (or patient's caretaker) give for coming to the health facility? *(Check a box for each listed complaint, or if not listed, legibly write the complaint next to "Other".)*

[] A7a. Fever/malaria	[] A7b. Diarrhoea/vomiting
[] A7c. Respiratory problem/cough/influenza	[] A7d. Ear problem
A7e. Other symptom: ____	
A7f. Other symptom: ____	
A7g. Other symptom: ____	
A7h. Other symptom: ____	

Does the health worker ask about the following signs and symptoms?
Check only one box per item. Check "Yes" if health worker asked; if health worker did not ask, then check either "No" or "Info. avail."
<u>Note:</u> "Info. avail." means the information was already available. Either the patient (or caretaker) spontaneously offers the information, or the patient has already given the information in response to a previous question, or the patient very obviously has the sign (e.g., a seizure).

Sign or symptom	Check one box
A8a. Fever	[] Yes (1) [] No (2) [] Info. avail. (3)
A8b. Headache	[] Yes (1) [] No (2) [] Info. avail. (3)
A8c. Joint pain	[] Yes (1) [] No (2) [] Info. avail. (3)
A8d. Vomiting	[] Yes (1) [] No (2) [] Info. avail. (3)
A8e. Chills	[] Yes (1) [] No (2) [] Info. avail. (3)
A8f. Poor appetite	[] Yes (1) [] No (2) [] Info. avail. (3)
A8g. Fatigue	[] Yes (1) [] No (2) [] Info. avail. (3)
A8h. Diarrhea	[] Yes (1) [] No (2) [] Info. avail. (3)
A8i. Cough	[] Yes (1) [] No (2) [] Info. avail. (3)
A8j. Problems urinating	[] Yes (1) [] No (2) [] Info. avail. (3)
A8k. Dark urine	[] Yes (1) [] No (2) [] Info. avail. (3)
A8l. Bleeding or bruising	[] Yes (1) [] No (2) [] Info. avail. (3)
A8m. Bloody stools	[] Yes (1) [] No (2) [] Info. avail. (3)
A8n. Ear pain	[] Yes (1) [] No (2) [] Info. avail. (3)
A8o. Nasal or sinus congestion	[] Yes (1) [] No (2) [] Info. avail. (3)
A8p. Convulsions	[] Yes (1) [] No (2) [] Info. avail. (3)
A8q. Is patient pregnant	[] Yes (1) [] No (2) [] Info. avail. (3)

Does the health worker (or another staff) perform the following clinical tasks?

Clinical task	Check one box
A9a. Take the patient's temperature	[] Yes (1) [] No (2) [] Unsure (3)
A9b. Weigh the patient	[] Yes (1) [] No (2) [] Unsure (3)
A9c. Take blood pressure	[] Yes (1) [] No (2) [] Unsure (3)
A9d. Take pulse (≥15 seconds, with timer or watch with second hand)	[] Yes (1) [] No (2) [] Unsure (3)
A9e. Examine inside of mouth	[] Yes (1) [] No (2) [] Unsure (3)
A9f. Examine palms	[] Yes (1) [] No (2) [] Unsure (3)
A9g. Examine fingertips	[] Yes (1) [] No (2) [] Unsure (3)
A9h. Count respiratory rate (≥15 seconds, with watch or timer)	[] Yes (1) [] No (2) [] Unsure (3)
A9i. Skin pinch	[] Yes (1) [] No (2) [] Unsure (3)
A9j. Offer drink of water	[] Yes (1) [] No (2) [] Unsure (3)

Health facility ID no. ___ ___ ___	Health worker ID no. ___ ___	Patient ID no. ___ ___ ___

Record results of laboratory testing done at the health facility

Laboratory test	Check box or record test result *(write "ND" if test not done)*
A10a1. Microscopy (positive, negative, indeterminate)	[] Positive (1) [] Negative (2) [] Indeterminate (3) [] Results not available today (5) [] ND (4)
A10a2. Microscopy (asexual parasite count or other quantification)	*Result:* ____________________ (include units, e.g., parasites/μl)
A10a3. Microscopy (species)	*Result:* ____________________
A10b1. Rapid diagnostic test (positive, negative, indeterminate)	[] Positive (1) [] Negative (2) [] Indeterminate (3) [] ND (4)
A10b2. Rapid diagnostic test (species)	*Result:* ____________________
A10c. Hemoglobin (g/dl)	*Result:* ____________________
A10d. Hematocrit (%)	*Result:* ____________________
Other test. A10e1. *Test:* ____________________________	A10e2. *Result:* ______________
Other test. A10f1. *Test:* ____________________________	A10f2. *Result:* ______________
Other test. A10g1. *Test:* ____________________________	A10g2. *Result:* ______________

After the consultation, ask the patient to wait outside. Ask the health worker for his/her name and record in question A2, and then ask for the patient's diagnosis. Keep asking "Any other diagnoses?" until the health worker has stated all diagnoses. If the health worker says malaria or anemia, ask if the illness is non-severe or severe. Check a box for each listed diagnosis, or if not listed, legibly write the diagnosis next to "Other".

[] A11a. Uncomplicated malaria	[] A11b. Severe/complicated malaria
[] A11c. Mild or moderate anemia	[] A11d. Severe anemia
[] A11e. Cold/influenza/upper respiratory illness	[] A11f. Pneumonia
[] A11g. Diarrhea	[] A11h. Dysentery
[] A11i. Otitis	[] A11j. Measles
[] A11k. Urinary tract infection	[] A11l. Hepatitis
A11m. Other: __________________________	A11n. Other: ________________________
A11o. Other: ____________________________	A11p. Other: ________________________

Record drugs that were administered to the patient during the consultation. Verify drugs and dosages with the health worker after the consultation.

Drug	Dose administered during consultation
A12a. Coartem tablets (20mg art./120mg lum.)	_____ tablets administered
A12b. Artesunate tablets (50mg)	_____ tablets administered
A12c. Amodiaquine tablets (153mg)	_____ tablets administered
A12d. Quinine sulfate tablets	_____ mg of drug administered
A12e. Quinine injection	_____ mg of drug administered
A12f. Quinidine injection	_____ mg of drug administered
A12g. Artemether injection	_____ mg of drug administered
A12h. Artesunate injection	_____ mg of drug administered
A12i. Artesunate suppositories	_____ mg of drug administered
A12j. Chloroquine tablets	_____ mg of drug administered
A12k. Primaquine tablets	_____ mg of drug administered
A12l. Sulfadoxine-pyrimethamine tablets (500mg sulf/25mg pyr)	_____ tablets administered
12m. Tetracycline	_____ mg of drug administered
12n. Doxycycline	_____ mg of drug administered
12o. Cotrimoxazole	_____ mg of drug administered
12p. Ampicillin	_____ mg of drug administered
12q. Amoxicillin	_____ mg of drug administered
12r. Oral rehydration solution	[] Check box if any quantity was administered
12s. Paracetamol or aspirin	[] Check box if any quantity was administered
12t1. Other drug: ________________________	12t2. Amount (e.g., in mg) _______________
12u1. Other drug: _______________________	12u2. Amount (e.g., in mg) _______________
12v1. Other drug: ________________________	12v2. Amount (e.g., in mg) _______________
12w1. Other drug: _______________________	12w2. Amount (e.g., in mg) _______________
12x1. Other drug: ________________________	12x2. Amount (e.g., in mg) _______________
12y1. Other drug: ________________________	12y2. Amount (e.g., in mg) _______________
12z1. Other drug: ________________________	12z2. Amount (e.g., in mg) _______________

12zz. [] Check this box if no medicines were administered during the consultation.

Health facility ID no. ___ ___ ___	Health worker ID no. ___ ___	Patient ID no. ___ ___ ___

Record prescriptions (verify with HW after the consultation) and whether instructions were given

Drug and concentration (*check box for listed drug, or write drug name next to "Other"; write concentration on line*)	Form (e.g., tablet, syrup, or injection)	Definition of one dose (in mg, for injections)	(*If pre-referral dose, write: "1 PR"*)		Did the health worker give complete instructions*? (*check one box*)
			Number of doses per day	Total days of treatment	
Ex 1. [X] Coartem (20/120mg)	tablet	3 tablets	2	3	[X] Yes (1) [ ] No (2)
Ex 2. [X] Quinine (300mg)	injection	90mg	1 PR	1 PR	[ ] Yes (1) [X] No (2)
[ ] A13a. Coartem (20/120mg)	tablet				[ ] Yes (1) [ ] No (2)
[ ] A13b. Artesunate (50mg)	tablet				[ ] Yes (1) [ ] No (2)
[ ] A13c. Amodiaquine (153mg)	tablet				[ ] Yes (1) [ ] No (2)
[ ] A13d. Quinine ___________	tablet				[ ] Yes (1) [ ] No (2)
[ ] A13e. Quinine ___________	injection				[ ] Yes (1) [ ] No (2)
[ ] A13f. Quinidine ___________	injection				[ ] Yes (1) [ ] No (2)
[ ] A13g. Artemether __________	injection				[ ] Yes (1) [ ] No (2)
[ ] A13h. Artesunate __________	injection				[ ] Yes (1) [ ] No (2)
[ ] A13i. Artesunate __________	suppository				[ ] Yes (1) [ ] No (2)
[ ] A13j. Chloroquine _________	tablet				[ ] Yes (1) [ ] No (2)
[ ] A13k. Primaquine _________	tablet				[ ] Yes (1) [ ] No (2)
[ ] A13l. Sulfadox-pyr. (500/25mg)	tablet				[ ] Yes (1) [ ] No (2)
[ ] A13m. Tetracycline ________					[ ] Yes (1) [ ] No (2)
[ ] A13n. Doxycycline ________					[ ] Yes (1) [ ] No (2)
[ ] A13o. Cotrimoxazole _______					[ ] Yes (1) [ ] No (2)
[ ] A13p. Ampicillin __________					[ ] Yes (1) [ ] No (2)
[ ] A13q. Amoxicillin _________					[ ] Yes (1) [ ] No (2)
[ ] A13r. Oral rehydrat. solution					
[ ] A13s. Paracetamol or aspirin					
[ ] A13t. Other _______________					[ ] Yes (1) [ ] No (2)
[ ] A13u. Other ______________					[ ] Yes (1) [ ] No (2)
[ ] A13v. Other ______________					[ ] Yes (1) [ ] No (2)
[ ] A13w. Other ______________					[ ] Yes (1) [ ] No (2)
[ ] A13x Other _______________					[ ] Yes (1) [ ] No (2)
[ ] A13y. Other ______________					[ ] Yes (1) [ ] No (2)
[ ] A13z. Other ______________					[ ] Yes (1) [ ] No (2)

* Includes definition of a dose, doses per day, and total days of treatment (or "until drugs are done").

13zz. [] Check this box if no medicines were prescribed.

Record counseling messages (*check Yes or No for each message given*)

Counseling messasge	Message given?
A14a1. Take the medicine with food.	[] Yes(1) [] No (2)
A14a2. Take the medicine with milk or fat-containing food.	[] Yes(1) [] No (2)
A14b. (You/your child) needs to be hospitalized.	[] Yes(1) [] No (2)
A14c. (You/your child) needs to be hospitalized **urgently**.	[] Yes(1) [] No (2)
A14d. (You/your child) should sleep under a bed net to prevent malaria.	[] Yes(1) [] No (2)
A14e. If (you/your child) become seriously ill, come back to the health facility.	[] Yes(1) [] No (2)
A14f. Come back to the health facility for control.	[] Yes(1) [] No (2)
A14g. Complete all the treatment (take all your medicines).	[] Yes(1) [] No (2)

Annex 3. Questionnaire for patient interview

Health facility ID no. ___ ___ ___	Health worker ID no. ___ ___	Patient ID no. ___ ___ ___

Patient's age.

*Complete **only one** of the following, but try to obtain the complete birthday.*
B1a. Patient's birthday: ___ / ___ / ______ (*day/month/year*)
B1b. *For patients >5 years old.* Patient's age in years: _____
B1c. *For children <5 years old.* Patient's age in months: _____

B2. The patient's age is: [] Less than 60 months old (1) [] 60 months old or older (2)

B3. Has the patient's weight been recorded in the health card/other document today? *(check only one response)*

[] Yes (1), the weight is: ___ ___.___ kg
[] No weight recorded (2)
[] No health card or other document (3)

B4. Has the patient's temperature been recorded in the health card/other document today? *(check only one response)*

[] Yes (1), the temperature is: ___ ___.___ EC
[] No temperature recorded (2)
[] No health card or other document (3)

B5. Did the health worker say that (you/your child) needs to be hospitalized (or stay at the health facility) for treatment? *(check only one response)*

[] Yes (1)
[] No (2)
[] Unsure (3)

B6. What illness did the health worker say (you/your child) had? *(Do not read the choices. Check all spontaneously mentioned by the caretaker and/or write diagnoses next to "Other". Keep asking "Any other diagnoses?" until the patient has stated all diagnoses.)*

[] B6a. Patient does not remember	[] B6b. Health worker did not tell diagnosis
[] B6c. Malaria	[] B6d. Diarrhea
[] B6e. Pneumonia	[] B6f. Cough/cold
[] B6g. Other (B6g1) ____	
[] B6h. Other (B6h1) ____	

Patient/caretaker comprehension of instructions for giving drugs. *Ask the patient/caretaker to show you all dispensed drugs. Below, check the box for listed drugs, or write the drug name next to "Other"; and write the concentration on the line. For each drug, ask the patient/caretaker how it should be given. Allow use of any materials given by the health worker. Ask the questions for each drug and each column. Write "DK" in the column if the caretaker does not know the instruction.*

Drug and concentration	Definition of one dose	Number of doses per day	Total days of treatment (or "until done")
Ex 1. [X] Coartem (20/120mg)	3 tablets	2	3
Ex 1. [X] __Amoxicillin 250mg__	1 tablet	DK	Until done
[ ] B7a. Coartem (20/120mg)			
[ ] B7b. Artesunate (50mg)			
[ ] B7c. Amodiaquine (153mg)			
[ ] B7d. Quinine ______________________			
[ ] B7f. Quinidine ______________________			
[ ] B7g. Artemether _____________________			
[ ] B7h. Artesunate _____________________			
[ ] B7j. Chloroquine ____________________			
[ ] B7k. Primaquine _____________________			
[ ] B7l. Sulfadoxine-pyrimethamine (500/25mg)			
[ ] B7m. Tetracycline _____________________			
[ ] B7n. Doxycycline ____________________			
[ ] B7o. Cotrimoxazole ___________________			
[ ] B7p. Ampicillin ______________________			
[ ] B7q. Amoxicillin _____________________			
[ ] B7r. Oral rehydration solution			
[ ] B7s. Paracetamol or aspirin			
[ ] B7t. Other _[Write "No medicines" if no medicines]__			
[ ] B7u. Other __________________________			
[ ] B7v. Other __________________________			
[ ] B7w. Other __________________________			
[ ] B7x Other ___________________________			
[ ] B7y. Other __________________________			
[ ] B7z. Other __________________________			

Health facility ID no. ___ ___ ___	Health worker ID no. ___ ___	Patient ID no. ___ ___ ___

Patient satisfaction.
<u>Introduction (read the following):</u> *I would like to ask you several questions about your visit to the health facility and the care (you / your child) received. I am asking these questions to know what you liked and disliked about the services you have received. I am going to use the information to know how the health facility functions and how the services could be improved.*

B7. In general, which of the four statements best describes how you feel about the services you have received today at the health facility? *(Read all 4 choices and check only one)*

[] I was very satisfied (1)
[] I was somewhat satisfied (2)
[] I was somewhat dissatisfied (3)
[] I was very dissatisfied (4)

B8. Please tell me one thing that you think would improve the care at this health facility?

____

B9. What is the most important way that a person gets malaria? *(Do not read the list. Check all that apply, and/or write response next to "Other".)*

[] B9a. Mosquitos, or a mosquito bite
[] B9b. Insects, or an insect bite (but mosquito not mentioned)
[] B9c. The sun
[] B9d. Witchcraft, or sorcery, or magic
[] B9e. Other (B9e1) ____
[] B9f. Do not know

B10. How can you protect yourself from getting malaria? *(Do not read the list. Check all that apply, and/or write response next to "Other".)*

[] B10a. Sleep under an insecticide-treated bednet
[] B10b. Sleep under a bednet (insecticide not mentioned)
[] B10c. Take medicine
[] B10d. Use insect repellent
[] B10e. Other (B10e1) ____
[] B10f. Do not know

B11. Did (you / your child) sleep under a bednet last night? *(Check one)*

[] Yes (1)
[] No (2)
[] Do not know (3)

Annex 4a. Re-examination form for patients <5 years old

C1. What reasons does the patient's caretaker give for coming to the health facility? *(Check a box for each listed complaint, or if not listed, write the complaint next to "Other".)*

[] C1a. Fever/malaria	[] C1b. Diarrhea/vomiting
[] C1c. Respiratory problem/cough/influenza	[] C1d. Ear problem
C1e. Other symptom: ____	
C1f. Other symptom: ____	
C1g. Other symptom: ____	

C2. When did the illness (this illness episode) first begin? *(Check one)*

[] Today (1)　　[] More than 2 days ago (4)
[] Yesterday (2)　　[] Don't know (5)
[] Two days ago (3)

C3. Take patient's temperature and record temperature: ____ ____ **.** ____ EC

Ask the patient's caretaker about the following symptoms since the beginning of the illness, and check for anemia.

Sign or symptom	Check one	
C4a. **Ask:** Fever?	[] Yes (1) [] No (2)	
C4b. **Ask:** Headache?	[] Yes (1) [] No (2)	Of these 9 "non-fever" symptoms, count the number of "Yes" responses and record result: _______ **Do not count fever.**
C4c. **Ask:** Joint pain?	[] Yes (1) [] No (2)	
C4d. **Ask:** Vomiting?	[] Yes (1) [] No (2)	
C4e. **Ask:** Chills?	[] Yes (1) [] No (2)	
C4f. **Ask:** Poor appetite?	[] Yes (1) [] No (2)	
C4g. **Ask:** Fatigue?	[] Yes (1) [] No (2)	
C4h. **Ask:** Diarrhea?	[] Yes (1) [] No (2)	
C4i. **Ask:** Cough?	[] Yes (1) [] No (2)	
C4j. **Check for anemia:** Pale palms, or pale mucosa, or pale fingertips?	[] Yes (1) [] No (2)	

DETERMINE IF THE PATIENT HAS SUSPECTED MALARIA.

- If the patient has fever (history or temperature $\geq$37.5EC axillary) OR at least 3 non-fever symptoms, then the patient has suspected malaria. **Go to question C5.**
- If the patient has no fever AND less than 3 non-fever symptoms, then the patient does not have suspected malaria. **Thank the caretaker and end the interview. The patient may go.**

Health facility ID no. ___ ___ ___	Health worker ID no. ___ ___	Patient ID no. ___ ___ ___

Note: Do not take a blood sample.

C5. Measure 60-second respiratory rate while patient is calm: _______ breaths per minute
(Fast breathing is >50/min. for ages <12 months, and >40/min. for ages 1 up to 5 years)

C6. Measure the patient's pulse while patient is calm: _______ beats per minute

C8. Weigh patient and record weight: ____ ____ ____ . ____ kg

Ask the patient's caretaker about the following symptoms since the beginning of the illness.

Sign	Check one	Sign	Check one
C9a. Difficult or painful urination	[] Yes (1) [] No (2)	C9b. Ear pain	[] Yes (1) [] No (2)
C9c. Little or no urine in past 24 hours	**[] Yes** (1) [] No (2)	C9d. Nasal or sinus congestion	[] Yes (1) [] No (2)
C9e. Dark urine	**[] Yes** (1) [] No (2)	C9f. Convulsions	**[] Yes** (1) [] No (2)
C9g. Bloody stools (dysentery)	**[] Yes** (1) [] No (2)	C9h. Bleeding	**[] Yes** (1) [] No (2)

Examine the patient to search for the following signs.

Sign	Check one	Sign	Check one
C10a. Convulsions	**[] Yes** (1) [] No (2)	C10b. Sunken eyes	[] Yes (1) [] No (2)
C10c. Fast breathing (see C5)	**[] Yes** (1) [] No (2)	C10d. Irritability or restlessness	[] Yes (1) [] No (2)
C10e. Temperature >41EC (see C3)	**[] Yes** (1) [] No (2)	C10f. Offer drink—patient drinks eagerly	[] Yes (1) [] No (2)
C10g. Respiratory distress	**[] Yes** (1) [] No (2)	C10h. Offer drink—patient drinks poorly	[] Yes (1) [] No (2)
C10i. Koplic spots	[] Yes (1) [] No (2)	C10j. Slow skin pinch	[] Yes (1) [] No (2)
C10k. Measles rash	[] Yes (1) [] No (2)	C10l. Jaundice	**[] Yes** (1) [] No (2)
C10m. Lethargy or unconsciousness	**[] Yes** (1) [] No (2)	C10n. Capillary refill >2 seconds	**[] Yes** (1) [] No (2)
		C10p. Bruising or bleeding	**[] Yes** (1) [] No (2)
C10q. Weak pulse and >110 beats/min. (see C6)	**[] Yes** (1) [] No (2)	C10r. Severe palmar pallor	**[] Yes** (1) [] No (2)
C10s. Cyanosis	**[] Yes** (1) [] No (2)		

Review lab data.

Test	Result
C11a. Hematocrit (see A10d)	**[] <20%** (1) [] $\geq$20% (2) [] Test not done (3)
C11b. Hemoglobin (see A10c)	**[]** <5 g/dl (1) [] $\geq$5 g/dl (2) [] Test not done (3)
C11c. Parasitemia parasites/µl (see A10a2)	**[]>100,000** (1) [] $\leq$100,000 (2) [] Test not done (3) [] Unsure (4)

<u>DETERMINE IF THE PATIENT HAS SEVERE MALARIA.</u>

In questions C9, C10, and C11, signs of severe malaria are indicated with shading and bold font for the "Yes" choice (e.g., "**[] Yes** (1)").

- If the patient has at least one of these signs, then the patient has severe malaria. **Ensure the patient receives an adequate pre-referral dose of an antimalarial and counsel the patient's caretaker to seek inpatient care urgently. Thank the caretaker. The patient may go.**
- If the patient has none of these signs, then the patient has uncomplicated malaria. **Ensure the patient receives adequate antimalarial treatment. Thank the caretaker. The patient may go.**

Annex 4b. Re-examination form for patients >5 years old

D1. What reasons does the patient (or patient's caretaker) give for coming to the health facility? *(Check a box for each listed complaint, or if not listed, write the complaint next to "Other".)*

[] D1a. Fever/malaria	[] D1b. Diarrhea/vomiting
[] D1c. Respiratory problem/cough/influenza	[] D1d. Ear problem
D1e. Other symptom: ______________________	
D1f. Other symptom: ______________________	
D1g. Other symptom: ______________________	

D2. When did the illness (this illness episode) first begin? *(Check one)*

[] Today (1) [] More than 2 days ago (4)
[] Yesterday (2) [] Don't know (5)
[] Two days ago (3)

D3. Take patient's temperature and record temperature: ____ ____ . ____ EC

Ask the patient or patient's caretaker about the following symptoms since the beginning of the illness, and check for anemia.

Sign or symptom	Check one	
D4a. **Ask:** Fever?	[] Yes (1) [] No (2)	
D4b. **Ask:** Headache?	[] Yes (1) [] No (2)	Of these 8 "non-fever" symptoms, count the number of "Yes" responses and record result: _______ **Do not count fever.**
D4c. **Ask:** Joint pain?	[] Yes (1) [] No (2)	
D4d. **Ask:** Vomiting?	[] Yes (1) [] No (2)	
D4e. **Ask:** Chills?	[] Yes (1) [] No (2)	
D4f. **Ask:** Poor appetite?	[] Yes (1) [] No (2)	
D4g. **Ask:** Fatigue?	[] Yes (1) [] No (2)	
D4h. **Ask:** Diarrhea?	[] Yes (1) [] No (2)	
D4j. **Check for anemia:** Pale palms, or pale mucosa, or pale fingertips?	[] Yes (1) [] No (2)	

DETERMINE IF THE PATIENT HAS SUSPECTED MALARIA.

- If the patient has fever (history or temperature >37.5EC axillary) OR at least 3 non-fever symptoms, then the patient has suspected malaria. **Go to question DX1.**
- If the patient has no fever AND less than 3 non-fever symptoms, then the patient does not have suspected malaria. **Thank the patient and end the interview. The patient may go.**

Health facility ID no. ___ ___ ___	Health worker ID no. ___ ___	Patient ID no. ___ ___ ___

DX1. Is the patient pregnant? *(Check one)*
- [] Yes (1) → **Go to question D5.**
- [] No (2) → **Go to question DX2.**
- [] Unsure (3) → **Go to question DX2 (assume not pregnant).**

DX2. The laboratory technician should perform a finger stick, prepare a malaria smear, and perform an RDT. Wait 15 minutes for the RDT (time with watch, timer, or cell phone clock). While the RDT is developing, continue with question D5. When RDT is ready, record result below.
- [] Positive (1)
- [] Negative (2)
- [] Indeterminate (3) → **Repeat rapid diagnostic test.**

D5. Measure 60-second respiratory rate while patient is calm: _______ breaths per minute
(Fast breathing is ≥30/min. for ages 5 up to 13 years, and ≥20/min. for ages 13+ years)

D6. Measure the patient's pulse while patient is calm: _______ beats per minute

D7. *If the patient is ≥15 years old:*
Measure the patient's blood pressure while patient is calm: ______ / ______ mm Hg

D8. Weigh patient and record weight: ____ ____ ____ . ____ kg

Ask the patient or the patient's caretaker about the following symptoms since the beginning of the illness.

Sign	Check one	Sign	Check one
D9a. Difficult or painful urination	[] Yes (1) [] No (2)	D9b. Ear pain	[] Yes (1) [] No (2)
D9c. Little or no urine in past 24 hours	**[] Yes** (1) [] No (2)	D9d. Nasal or sinus Congestion	[] Yes (1) [] No (2)
D9e. Dark urine	**[] Yes** (1) [] No (2)	D9f. Convulsions	**[] Yes** (1) [] No (2)
D9g. Bloody stools(dysentery)	**[] Yes** (1) [] No (2)	D9h. Bleeding	**[] Yes** (1) [] No (2)

Examine the patient to search for the following signs.

Sign	Check one	Sign	Check one
D10a. Convulsions	**[] Yes** (1) [] No (2)	D10b. Sunken eyes	[] Yes (1) [] No (2)
D10c. Fast breathing (see D5)	**[] Yes** (1) [] No (2)	D10d. Irritability or restlessness	[] Yes (1) [] No (2)
D10e. Temperature >41EC (see D3)	**[] Yes** (1) [] No (2)	D10f. Offer drink— patient drinks eagerly	[] Yes (1) [] No (2)
D10g. Respiratory distress	**[] Yes** (1) [] No (2)	D10h. Offer drink— patient drinks poorly	[] Yes (1) [] No (2)
D10i. Koplic spots	[] Yes (1) [] No (2)	D10j. Slow skin pinch	[] Yes (1) [] No (2)
D10k. Measles rash	[] Yes (1) [] No (2)	D10l. Jaundice	**[] Yes** (1) [] No (2)
D10m. Lethargy or unconsciousness	**[] Yes** (1) [] No (2)	D10n. Capillary refill >2 sec.	**[] Yes** (1) [] No (2)
D10o. Systolic BP <80 (see D7)	**[] Yes** (1) [] No (2)	D10p. Bruising or bleeding	**[] Yes** (1) [] No (2)
D10q. Weak pulse and >110 beats/minute (see D6)	**[] Yes** (1) [] No (2)	D10r. Severe palmar pallor	**[] Yes** (1) [] No (2)
D10s. Cyanosis	**[] Yes** (1) [] No (2)		

Review lab data.

Test	Result
D11a. Hematocrit (see A10d)	**[] <20%** (1) [] ≥20% (2) [] Test not done (3)
D11b. Hemoglobin (see A10c)	**[] <5 g/dl** (1) [] ≥5 g/dl (2) [] Test not done (3)
D11c. Parasitemia parasites/µl (see A10a2)	**[]>100,000** (1) [] ≤100,000 (2) [] Test not done (3) [] Unsure (4)

DETERMINE IF THE PATIENT HAS MALARIA.

- If the patient is not pregnant AND the rapid diagnostic test is negative, then the patient does not have malaria. If the health worker prescribed an antimalarial, tell the patient to take it anyway. **Thank the patient. The patient may go.**
- If the patient is not pregnant AND the rapid diagnostic test is positive, then the patient has malaria. **Proceed to the next section "Determine if the patient has severe malaria."**
- If the patient is pregnant, then she must be treated for malaria. **Proceed to the next section "Determine if the patient has severe malaria."**

DETERMINE IF THE PATIENT HAS SEVERE MALARIA.

In questions D9, D10, and D11, signs of severe malaria are indicated with shading and bold font for the "Yes" choice (e.g., "**[] Yes** (1)").

- If the patient has at least one of these signs, then the patient has severe malaria. **Ensure the patient receives an adequate pre-referral dose of an antimalarial and counsel the patient (or patient's caretaker) to seek inpatient care urgently. Thank the caretaker (or patient's caretaker). The patient may go.**
- If the patient has none of these signs, then the patient has uncomplicated malaria. **Ensure the patient receives adequate antimalarial treatment. Thank the patient (or patient's caretaker). The patient may go.**

Annex 5. HW interview

Health facility ID no. ___ ___ ___ Health worker ID no. ___ ___

Read this to the health worker: *I would like to take a few minutes to ask you some questions about your training, supervision, and knowledge.*

E1. Date :_____/_____/________ (*day/month/year*)

E2. Health worker's name: ________________________________

E3. Health worker's age ? _________ years

E4. Sex ? (*Check one*) [] Male (1) [] Female (2)

E5. What is the health worker's qualification or pre-service training? (*Check only one response*)
- [] E5a. Physician
- [] E5b. Nurse (Enfermeiro Basico)
- [] E5c. Nurse (Enfermeiro Geral) [Implies "Enfermeiro superior"]
- [] E5d. Nurse (Enfermeiro Medio)
- [] E5e. Nurse (Enfermeiro Especializado) [Implies "Enfermeiro superior"]
- [] E5f. Other: ____

E6. How many years of medical training did you receive? _______ years

Read this to the health worker: *I will now ask some questions about in-service training courses that you have attended. This means training courses on health topics that you have received after you finished basic training and after you had begun working as a health worker. Do not count informal teaching when one health worker teaches another health worker something.*

E7. Record details on other in-service training courses—*except for training on Coartem.*

Topic of "formal" in-service training	Did the health worker participate?	Year of training (most recent year if >1 training)	Training duration, in days (most recent duration if >1 training)
E7a. Integrated Management of Childhood Illness (IMCI)	[] Yes (1) [] No (2)		______ days
E7b. Diarrhea case management	[] Yes (1) [] No (2)		______ days
E7c. Acute respiratory illness case management	[] Yes (1) [] No (2)		______ days
E7d. Immunizations	[] Yes (1) [] No (2)		______ days
E7e. Nutrition	[] Yes (1) [] No (2)		______ days
E7f. Other: ____________________	[] Yes (1) [] No (2)		______ days
E7g. Other: ____________________	[] Yes (1) [] No (2)		______ days
E7h. Other: ____________________	[] Yes (1) [] No (2)		______ days

E8a. Did you attend a "formal" in-service training course on malaria case management that taught how to use artemisinin-based combination therapy (or ACTs), such as Coartem?		[] Yes (1) [] No (2) [] Unsure (3)<br>*If "No" or "Unsure", go to question E8f.*
	E8b. *If yes to E8a, ask:* How many times did you receive this training?	______ times
	E8c. *If yes to E8a, ask:* Did the malaria course train you to use malaria rapid diagnostic tests, such as Paracheck?	[] Yes (1) [] No (2) [] Unsure (3)
	E8d. *If yes to E8a, ask:* Think about your last training. When was your last training?	_____ / ______ (month/year)
	E8e. *If yes to E8a, ask:* Think about your last training. How many days did the training last?	______ days

E8f. Sometimes when health workers receive in-service training, they are asked to go back to their health facility and informally teach other health workers who did not attend the in-service training course. Did you receive this kind of **informal** teaching on treating malaria cases with ACTs, such as Coartem?	*Check one box*<br>[] Yes (1)<br>[] No (2)<br>[] Unsure (3)

E9. How many times did you receive supervision in the last 6 months? …. _______

E10. How many times did you receive supervision in the last 6 months in which the supervisor observed you performing a consultation and provided feedback on your performance? ………………………………… _______
(Note: the number in E10 must be less than or equal to the number in E9)

E10a. How many times did you receive supervision in the past 6 months on the use of Coartem? …………………………………………….. ________

E11. *Ask the health worker:* In the consultation room today, did you have any ACT job aid (e.g., ACT policy document, or clinical algorithm, or dosage guide)? *(Check only one)*
[] Yes (1) [] No (2)

E12. How many patients (all ages) did you see in consultation today? ________

Read this to the health worker: I will now ask some questions about how you treat ill patients who might or might not have malaria. This is not a test. Feel free to look at any documents. However, it is important that you tell us how you actually treat real ill patients.

E13. Describe in as much detail as possible how you decide which patients should be tested for malaria with microscopy or with a rapid diagnostic test (such as Paracheck)? (*Write neatly*)

____

____

Health facility ID no. ___ ___ ___ Health worker ID no. ___ ___

<u>Read this to the health worker:</u> In the following scenarios, assume that all essential medicines are available and that a hospital is 5 kilometers from the health facility.

Scenario (*read to health worker*)	Diagnosis, treatment, and need for hospitalization (ask for specific drug names, but not dosages)
E14. A 30-year old man with fever (temperature is 39ºC), fatigue, and no other symptoms. A malaria RDT is negative.	a. Diagnosis(es)?
	b. Treatment(s)?
	c. Hospitalization needed? [] Yes (1) [] No (2) [] Unsure (3)
E15. A 25-year old pregnant woman with fever (temperature is 38ºC), headache, and no other symptoms. She has been pregnant for 2 months. A malaria RDT is negative.	a. Diagnosis(es)?
	b. Treatment(s)?
	c. Hospitalization needed? [] Yes (1) [] No (2) [] Unsure (3)
E17. A 41-year old man with fever (temperature is 39ºC), fatigue, and no other symptoms. Microscopy is negative for malaria.	a. Diagnosis(es)?
	b. Treatment(s)?
	c. Hospitalization needed? [] Yes (1) [] No (2) [] Unsure (3)
E18. A 32-year old woman with fever and fatigue. She had a convulsion in the morning, but is awake now. No other symptoms. Microscopy was positive for malaria.	a. Diagnosis(es)?
	b. Treatment(s)?
	c. Hospitalization needed? [] Yes (1) [] No (2) [] Unsure (3)

<u>Read to the health worker:</u> For the next questions, imagine you are working in the outpatient department of a hospital.

Case 1. A 22-year old man comes to see you with 2 days of headache, vomiting, fatigue, and joint pain. He states he does not have fever, and he has no other symptoms. Although he is tired, he does not appear critically ill. His temperature is normal (36.5ºC), and vital signs are normal.

E20a. Do you need to order any lab tests for this patient? Tell me what you really do in your practice.

[] Yes (1) → Go to the next question
[] No (2) → Go to question E21.
[] Unsure (3) → Go to question E21.

E20b. <u>*If yes to question E20a, ask:*</u> What tests you would order? (*Do not read list. Check all responses.*)

[] E20b1. Malaria rapid diagnostic test
[] E20b2. Blood smear/microscopy for malaria
[] E20b3. Other (specify): ____________________
[] E20b4. Other (specify): ____________________

Case 2. A 4-year old child comes to a consultation with fever.

E21a. Do you need to order any lab tests for this patient? Tell me what you really do in your practice.

[] Yes (1) → Go to the next question
[] No (2) → Go to question E22.
[] Unsure (3) → Go to question E22.

E21b. <u>*If yes to question E21a, ask:*</u> What tests you would order? (*Do not read list. Check all responses.*)

[] E21b1. Malaria rapid diagnostic test
[] E21b2. Blood smear/microscopy for malaria
[] E21b3. Other (specify): ____________________
[] E21b4. Other (specify): ____________________

Case 3. A 4-year old child comes to a consultation with diarrhea, chills, fatigue, and poor appetite. The mother states that the child did not have fever, and the temperature is normal (36.4ºC).

E22a. Do you need to order any lab tests for this patient? Tell me what you really do in your practice.

[] Yes (1) → Go to the next question
[] No (2) → End the interview.
[] Unsure (3) → End the interview.

E22b. <u>*If yes to question E22a, ask:*</u> What tests you would order? (*Do not read list. Check all responses.*)

[] E22b1. Malaria rapid diagnostic test
[] E22b2. Blood smear/microscopy for malaria
[] E22b3. Other (specify): ____________________
[] E22b4. Other (specify): ____________________

INSTRUCTIONS: After the last question, provide answers to the questions, and thank the health worker.

Answers to health worker knowledge assessment

Scenario (*read to health worker*)	Diagnosis, treatment, and need for hospitalization (ask for specific drug names, but not dosages)
E14. A 30-year old man with fever (temperature is 39ºC), fatigue, and no other symptoms. A malaria RDT is negative.	a. Diagnosis(es)? Unexplained fever (not malaria)
	b. Treatment(s)? Follow-up in 48 hours (give symptomatic treatment)
	c. Hospitalization needed? [] Yes (1) [X] No (2) [] Unsure (3)
E15. A 25-year old pregnant woman with fever (temperature is 38ºC), headache, and no other symptoms. She has been pregnant for 2 months. A malaria RDT is negative.	a. Diagnosis(es)? Unexplained fever (not malaria)
	b. Treatment(s)? Follow-up in 48 hours (give symptomatic treatment)
	c. Hospitalization needed? [] Yes (1) [X] No (2) [] Unsure (3)
E17. A 41-year old man with fever (temperature is 39ºC), fatigue, and no other symptoms. Microscopy is negative for malaria.	a. Diagnosis(es)? Unexplained fever (not malaria)
	b. Treatment(s)? Follow-up in 48 hours (give symptomatic treatment)
	c. Hospitalization needed? [] Yes (1) [X] No (2) [] Unsure (3)
E18. A 32-year old woman with fever and fatigue. She had a convulsion in the morning, but is awake now. No other symptoms. Microscopy was positive for malaria.	a. Diagnosis(es)? Severe malaria
	b. Treatment(s)? Injectable quinine
	c. Hospitalization needed? [X] Yes (1) [] No (2) [] Unsure (3)

Case 1. A 22-year old man comes to see you with 2 days of headache, vomiting, fatigue, and joint pain. He states he does not have fever, and he has no other symptoms. Although he is tired, he does not appear critically ill. His temperature is normal (36.5ºC), and vital signs are normal.

E20a. Do you need to order any lab tests for this patient? The answer is "Yes".

E20b. What tests you would order? The answer is "microscopy or RDT".

Case 2. A 4-year old child comes to a consultation with fever.

E21a. Do you need to order any lab tests for this patient? The answer is "No".

Case 3. A 4-year old child comes to a consultation with diarrhea, chills, fatigue, and poor appetite. The mother states that the child did not have fever, and the temperature is normal (36.4ºC).

E22a. Do you need to order any lab tests for this patient? The answer is "Yes".

E22b. What tests you would order? The answer is "microscopy or RDT".

Annex 6. HF assessment

F1. Health facility identification number: ____ ____ ____

F2. Health facility name: ____

F3. Municipality (*check one box*)

[] F3a. Bailundo	[] F3b. Caála	[] F3c. Ekunha
[] F3d. Huambo	[] F3e. Katchihungu	[] F3f. Londuimbali
[] F3g. Longonjo	[] F3h. Mungo	[] F3i. Tchikala Tchilohanga
[] F3j. Ukuma	[] F3k. Tchinjeje	

F4. Date: ____ / ____ / _______ (*day/month/year*)

F5. Health facility type (*Check one box*)

[] F5a. Provincial hospital
[] F5b. Municipal hospital
[] F5c. Hospital (other—neither provincial nor municipal)
[] F5d. Health center
[] F5e. Health post
[] F5f. Other (specify) [F5f1] ____

F6. Type of service (*Check only one response*)

[] F6a. Government-run/public health facility
[] F6b. For profit private (non-government) health facility (could be church-run)
[] F6c. Not for profit private (non-government) health facility (could be church-run)
[] F6d. Other (specify) [F6d1] ____

Caseload today

Check the health facility patient registration book. The following questions are for patients seen today at the health facility (whether or not the patient was enrolled in the survey) during normal working hours (8am to 3pm). Note that the numbers below should match information collected by the driver. If not, try to understand why there are differences. Record the most accurate numbers below.

F7. For patients **less than 5 years old**, how many consultations **(all types)**? _______

F8. For patients **less than 5 years old**, how many **initial** consultations? _______

F9. For patients **5 years old and older**, how many consultations **(all types)**? _______

F10. For patients **5 years old and older**, how many **initial** consultations? _______

F11. Staffing. *Complete the table with responses from the person in charge of the facility today.*

Health worker category	Number of health workers assigned to this facility	Number of health workers: *(formal training only)*			
		.... trained to use Coartem or other ACTs	.... trained to use RDTs	… who received IPTp training	... who received IMCI training
Physician	F11a1. ______	F11a2. ______	F11a3. _____	F11a4. ______	F11a5. ______
Nurse	F11b1. ______	F11b2. ______	F11b3. _____	F11b4. ______	F11b5. ______
Midwife	F11c1. ______	F11c2. ______	F11c3. _____	F11c4. ______	F11c5. ______
Health assistant or nursing aid	F11d1. ______	F11d2. ______	F11d3. _____	F11d4. ______	F11d5. ______
Lab workers	F11e1. ______	F11e2. ______	F11e3. _____	F11e4. ______	F11e5. ______
Other	F11f1. ______	F11f2. ______	F11f3. _____	F11f4. ______	F11f5. ______

F12. Equipment, supplies, and staffing for diagnostic testing

*Were the following **observed** at the facility, in or near at least one consultation room?*	
F12a. A thermometer (any type).	[] Yes (1) [] No (2)
F12b. A functional scale for weighing children.	[] Yes (1) [] No (2)
F12c. A copy of national guidelines on ACT use for children and adults.	[] Yes (1) [] No (2)
F12d. A booklet or chart with nationally recommended ACT treatment algorithms for children and adults.	[] Yes (1) [] No (2)
F12e. A watch with a second hand or a timer for counting respirations.	[] Yes (1) [] No (2)
*Were the following **observed** at the facility?*	
F12f. A staff person who can perform microscopy.	[] Yes (1) [] No (2)
F12g. A functional microscope (ask microscopist if it is functional).	[] Yes (1) [] No (2)
F12h. Glass slides and Giemsa stain for at least 25 malaria smears.	[] Yes (1) [] No (2)
F12i. A staff person who was trained perform rapid diagnostic tests.	[] Yes (1) [] No (2)
F12j. At least 25 rapid diagnostic tests (not expired).	[] Yes (1) [] No (2)

F13. Are mosquito bed nets available at the facility for distribution? *(Check all that apply and record the cost, when applicable)*

[] No bed nets for distribution
[] Yes, conventional bed nets (not long-lasting) available for free
[] Yes, conventional bed nets (not long-lasting) available for sale → Cost: _________
[] Yes, long-lasting bed nets available for free
[] Yes, long-lasting bed nets available for sale → Cost: _________

F13. Medicine stocks. *For columns 1 and 2, observe directly. For column 3, verify from log books or stock cards. For column 3, check DK (Don't Know) if it is not clear whether the medicine was in stock every day for the past 3 months. If "no" is checked in column 1, then "no" must be checked in columns 2 and 3.*

Name of medicine	In stock today? [column 1]	Are there at least 20 blister packs (not expired) in stock today? [column 2]	In stock every day (including today) for the past 3 months, according to log books or stock cards? [column 3]
Antimalarials			
Coartem blister [B6] (for 5–14 kg)	F13a1. [] Yes (1) [] No (2)	F13a2. [] Yes (1) [] No (2)	F13a3. [] Yes (1) [] No (2) [] DK (3)
Coartem blister [B12] (for 15–24 kg)	F13b1. [] Yes (1) [] No (2)	F13b2. [] Yes (1) [] No (2)	F13b3. [] Yes (1) [] No (2) [] DK (3)
Coartem blister [B18] (for 25–34 kg)	F13c1. [] Yes (1) [] No (2)	F13c2. [] Yes (1) [] No (2)	F13c3. [] Yes (1) [] No (2) [] DK (3)
Coartem blister [B24] (for ≥35 kg)	F13d1. [] Yes (1) [] No (2)	F13d2. [] Yes (1) [] No (2)	F13d3. [] Yes (1) [] No (2) [] DK (3)
Artesunate tablets	F13f1. [] Yes (1) [] No (2)		F13f3. [] Yes (1) [] No (2) [] DK (3)
Amodiaquine tablets	F13g1. [] Yes (1) [] No (2)		F13g3. [] Yes (1) [] No (2) [] DK (3)
Artemether (IM injectable)	F13h1. [] Yes (1) [] No (2)		F13h3. [] Yes (1) [] No (2) [] DK (3)
Artesunate (IV injectable)	F13i1. [] Yes (1) [] No (2)		F13i3. [] Yes (1) [] No (2) [] DK (3)
Artemisinin suppositories	F13j1. [] Yes (1) [] No (2)		F13j3. [] Yes (1) [] No (2) [] DK (3)
Artesunate suppositories	F13k1. [] Yes (1) [] No (2)		F13k3. [] Yes (1) [] No (2) [] DK (3)
Quinine (tablets)	F13l1. [] Yes (1) [] No (2)		F13l3. [] Yes (1) [] No (2) [] DK (3)
Quinine or quinidine (injectable)	F13m1. [] Yes (1) [] No (2)		F13m3. [] Yes (1) [] No (2) [] DK (3)
Other medicines			
Oral antibiotic (amoxicillin or ampicillin or cotrimoxazole or erythromycin)	F13n1. [] Yes (1) [] No (2)		F13n3. [] Yes (1) [] No (2) [] DK (3)
Iron	F13o1. [] Yes (1) [] No (2)		F13o3. [] Yes (1) [] No (2) [] DK (3)
Oral rehydration salts/solution	F13lp. [] Yes (1) [] No (2)		F13p3. [] Yes (1) [] No (2) [] DK (3)

F14. Details on patient sampling today.

Question	Response
F14a. Estimated number of patients expected today. *(Use this number to determine sampling fraction. Average of patients in the past 5 weekdays.)*	
F14b. Sampling fraction selected for today. *(Check sampling guide.)*	
F14c. Random starting number for today.	
F14d. Number of **ineligible** patients. *(Number of ill patients coming to see a health worker for a follow-up consultation. This is the last number circled in the follow-up table/box of the driver's form.)*	
F14e. Number of **eligible** patients. *(Number of ill patients coming to see a health worker for an initial consultation. This is the last number circled in the initial consultation table/box of the driver's form.)*	
F14f. Number of eligible patients that were **selected**. *(Number of eligible patients to whom the driver gave an identification card. Remember to include patients even if surveyors were not able to ask for consent. Number of yellow-highlighted boxes that have been circled on the driver's form.)*	
F14g. Number of eligible, selected patients that were met by a surveyor and asked to participate. *(Include all eligible, selected patients that were asked—regardless of whether or not they agreed to participate. Count the number of patients recorded in the verbal consent form for patients with the surveyors—both in column "Accept" and "Refuse".)*	
F14h. Number of eligible, selected patients that were met by a surveyor and **agreed** to participate. *(Number of patients that were **enrolled**. Count the number of patients recorded in the verbal consent form for patients with the surveyors—in column "Accept" only.)*<br><br>*Note: F14h should be the total number of patient questionnaires you have completed today. If a patient initially agrees to participate, but then withdraws before the survey is done, then record the number in F14j.*	
F14i. Number of eligible, selected patients that were met by a surveyor and **did not agree** to participate. *(Number of patients that **refused**. Count the number of patients recorded in the verbal consent form for patients with the surveyors—in column "Refuse" only.)*<br><br>*Note: F14g should equal F14h + F14i.*	
F14j. Number of enrolled patients who withdrew before the survey is done. *(Record "0" if there were none.)*	

Annex 7. Verbal consent for health workers (HWs)

Read the following to the HW.

The Ministry of Health of Angola is doing a survey of the care of ill patients at clinics in Huambo. The survey is being done with the Centers for Disease Control and Prevention in Atlanta, United States. We are doing the survey to find ways to improve the care ill patients receive. If you agree to be in the survey, we will watch you as you care for patients. At the end of the day, we will ask some questions. It will probably take less than 30 minutes to ask you the questions. The information we collect from these activities will be kept private, as much as the law allows. Participation is voluntary, which means you do not have to be in the survey. If you agree to be in the survey, at any time you may withdraw without penalty. If you choose not to be in the survey, there will be no penalty. Being in the survey is unlikely to involve any risks or discomforts.

Do you have any questions?

Answer any questions the HW asks.

Will you agree to be in the survey?

Annex 8. Verbal consent for patients

Verbal consent for patients <5 years old

Health facility name: ____

Health facility number: ____ ____ ____   Date of the visit: _____/_____/_____ (day/mon/yr)

Read the following to the patient's caretaker.

The Ministry of Health of Angola is doing a survey of the care of ill patients at clinics in Huambo. The survey is being done with the Centers for Disease Control and Prevention in Atlanta, United States. We are doing the survey to find ways to improve the care ill patients receive. If you agree to be in the survey, we will watch the health worker take care of your child. Then we will ask you some questions. It will take about 20 minutes to ask you the questions. Your answers will be kept private, as much as the law allows. Then a health worker from our team will examine your child in a room set aside for people in the survey. After the examination, we might change the medicines you got or give you additional medicines. We might make these changes because new medicines are recommended to treat malaria, and we are helping health workers to use these new medicines. Your child will receive the same care as patients not in the survey.

Being in the study is unlikely to bring any risks or discomforts. The information we collect might be useful for treating your child's current illness. Your participation is voluntary, which means you do not have to be in the survey. If you agree to be in the survey, at any time you may choose to withdraw without a problem. If you choose not to be in the survey, there will be no problem. For example, it will not affect the care your child will receive. Do you have any questions?

Answer any questions the patient asks.

Will you agree to be in the survey? (Record the patient ID number in the corresponding column. Separate ID numbers with commas.)

Accept	Refuse

If the patient agrees to participate, ask his/her name and record it in the observation form.

Verbal consent for patients 5 years of age and older

Health facility name: ____

Health facility number: ____ ____ ____ Date of the visit: _____/_____/_____ (day/mon/yr)

Read the following to the patient (or the patient's caretaker if the patient is a child).

The Ministry of Health of Angola is doing a survey of the care of ill patients at clinics in Huambo. The survey is being done with the Centers for Disease Control and Prevention in Atlanta, United States. We are doing the survey to find ways to improve the care ill patients receive. If you agree to be in the survey, we will watch the health worker take care of (you / your child). Then we will ask you some questions. It will take about 20 minutes to ask you the questions. Your answers will be kept private, as much as the law allows. Then a health worker from our team will examine (you / your child) in a room set aside for people in the survey. During the examination, the health worker might take a few drops of blood from (your / your child's) finger. We might do this even if blood was drawn during (your / your child's) consultation. We will use these drops to check for malaria. We will not use the blood to test for any other illnesses. After the examination, we might change the medicines you got or give you additional medicines. We might make these changes because new medicines are recommended to treat malaria, and we are helping health workers to use these new medicines. (You / your child) will receive the same care as patients not in the survey.

Being in the study is unlikely to bring any risks or discomforts. (You / your child) might feel a brief moment of pain or fear as (your / your child's) finger is pricked. There is rarely some bruising or infection at the site. The information we collect might be useful for treating your current illness. Your participation is voluntary, which means you do not have to be in the survey. If you agree to be in the survey, at any time you may choose to withdraw without a problem. If you choose not to be in the survey, there will be no problem. For example, it will not affect the care (you / your child) will receive. Do you have any questions?

Answer any questions the patient asks.

Will you agree to be in the survey? (Record the patient ID number in the corresponding column. Separate ID numbers with commas.)

Accept	Refuse

If the patient agrees to participate, ask his/her name and record it in the observation form.

Annex 9. Assessing health worker knowledge

Part 1. Coding open-ended question on how health workers select patients for malaria testing (microscopy or rapid diagnostic test) [question E13]

"E13. Describe in as much detail as possible how you decide which patients should be tested for malaria with microscopy or with a rapid diagnostic test (such as Paracheck)?"

Variable in dataset	Description	Coding
E13_VAR1	Did the health worker repeat the complete definition of suspected malaria cases to be tested according to the "old" policy in Huambo, Angola?<br><br>Complete definition: All patients with suspected malaria should be tested. Suspected malaria is defined as either fever (history of fever or axillary temperature >37.5ºC), or at least 3 of the following: headache, joint pain, chills, sweating, anemia (no explicit definition), cough (applies to children only), anorexia, fatigue, vomiting, or diarrhea.	Yes/No
E13_VAR2	What percentage of the criteria in the above definition (of the "old" Huambo policy) did the health worker repeat?<br><br>There are 14 criteria in the definition:<br>1) "History of fever" or "fever" or "elevated temperature"<br>2) Temperature >37.5°C<br>3) "At least 3 of the following"<br>4) Headache<br>5) Joint pain<br>6) Chills<br>7) Sweating<br>8) Anemia<br>9) Cough<br>10) Applies to children only (i.e., for cough)<br>11) Anorexia<br>12) Fatigue<br>13) Vomiting<br>14) Diarrhea	% (0– 100%)
E13_VAR3	Did the health worker repeat the complete definition of suspected malaria cases to be tested according to the "new" policy in Huambo, Angola?<br><br>Complete definition:<br><br>• Patients <5 years-old do not need testing.<br><br>• Patients ≥5 years-old: All patients with suspected malaria should be tested. Suspected malaria is defined as either fever (history of fever or axillary temperature >37.5ºC), or at least 3 of the following: headache, joint pain, chills, sweating, anemia (no explicit definition), anorexia, fatigue, vomiting, or diarrhea.	Yes/No

Coding of question E13, continued.

Variable in dataset	Description	Coding
E13_VAR4	What percentage of the criteria in the above definition (of the "new" Huambo policy) did the health worker repeat?<br><br>There are 14 criteria in the definition:<br>1) Patients <5 years old do not need testing<br>2) Patients ≥5 years old (age range mentioned)<br>3) "History of fever" or "fever" or "elevated temperature"<br>4) Temperature >37.5°C<br>5) "At least 3 of the following"<br>6) Headache<br>7) Joint pain<br>8) Chills<br>9) Sweating<br>10) Anemia<br>11) Anorexia<br>12) Fatigue<br>13) Vomiting<br>14) Diarrhea	% (0–100%)
E13_VAR5	Did the health worker mention fever in his/her response as criteria for suspected malaria case?	Yes/No
E13_VAR6	What age ranges are mentioned by the health worker in his/her response as criteria for suspected malaria case?	1) <5 or "child"<br>2) ≥5<br>3) Both <5 and ≥5<br>4) Other age mentioned<br>5) Age not mentioned
E13_VAR7	Does the health worker mention "≥ 3 or more (*non-fever*) symptoms" in his/her response as criteria for suspected malaria case?<br><br>The non-fever elements are: headache, joint pain, chills, sweats, anemia, cough (for <5's only), anorexia, fatigue, vomiting or diarrhea. The phrase "3 or more of the following" and any combination of the 3 non-fever elements stated above must be in the response.	Yes/No
E13_VAR8	Does the health worker mention signs, symptoms, or other elements that are not in the Huambo policy as criteria for a suspected malaria case in his/her response? (i.e., are "extra" elements added?)	Yes/No
E13_VAR9	Does the health worker mention < 5 year-olds not tested as criteria for a suspected malaria case in his/her response?	Yes/No
E13_VAR10	Does the health worker answer the question about criteria for suspected malaria in his/her response? Code this variable as "No" if the response clearly indicates that the health worker did not understand the question (e.g.., the health worker only describes how he/she would treat malaria).	Yes/No
E13_VAR11	Does the health worker mention extra elements that are not in the Huambo policy (see E13_VAR8), excluding the element "test patients who failed previous treatment", which is a reasonable response and not part of guidelines for managing a patient seen at an initial consultation?	Yes/No
